# Charting the circulating proteome in ME/CFS using cross-system profiling to uncover mechanistic insights

**DOI:** 10.1016/j.xcrm.2026.102647

**Published:** 2026-03-04

**Authors:** August Hoel, Fredrik Hoel, Sissel Elisabeth Dyrstad, Henrique Chapola, Ingrid Gurvin Rekeland, Kristin Risa, Kine Alme, Kari Sørland, Karl Albert Brokstad, Hans-Peter Marti, Olav Mella, Øystein Fluge, Karl Johan Tronstad

**Affiliations:** 1 Department of Biomedicine, University of Bergen, Bergen, Norway; 2 Department of Clinical Medicine, University of Bergen, Bergen, Norway; 3 Cancer Clinic, Haukeland University Hospital, Bergen, Norway; 4 Department of Safety, Chemistry and Biomedical Laboratory Sciences, Western Norway University of Applied Sciences, Bergen, Norway; 5 Department of Medicine, Haukeland University Hospital, Bergen, Norway; 6 Department of Clinical Science, University of Bergen, Bergen, Norway

**Keywords:** myalgic encephalomyelitis/chronic fatigue syndrome, ME/CFS, serum proteomics, pathomechanism, immune system, metabolism, vascular regulation

## Abstract

Myalgic encephalomyelitis/chronic fatigue syndrome (ME/CFS) is a debilitating condition often triggered by infections, with unclear mechanisms and no established biomarkers or treatments. We apply aptamer-based serum proteomics to 50 ME/CFS patients and 29 healthy controls, analyzing 7,326 protein targets. We identify 1,823 aptamers with significant differences between the groups (845 after false discovery rate [FDR] correction). Distinct patterns of tissue- and process-specific changes are seen. There is a broad increase in secreted proteins, while intracellular proteins, e.g., from skeletal muscle, particularly show reduction. Immune cell-associated signatures indicate immune reprogramming, including a distinct reduction in proteins secreted by activated neutrophils. Focused secretome analysis supports intensified regulatory interactions related to immune activity, inflammation, vasculature, and metabolism. Validation of measurements using antibody-based methods confirms findings for a selection of proteins. The uncovered serum proteome patterns in ME/CFS patients may contribute to understanding the pathophysiology and inform future biomarker research and therapeutic development.

## Highlights

• Serum proteomics reveals widespread protein changes in ME/CFS patients
• Tissue-linked shifts show reduced intracellular and increased secreted proteins
• Immune signatures show reprogramming with reduced neutrophil-derived proteins
• Regulatory networks link immune, vascular, and metabolic dysfunction

## Graphical abstract

**Figure:**
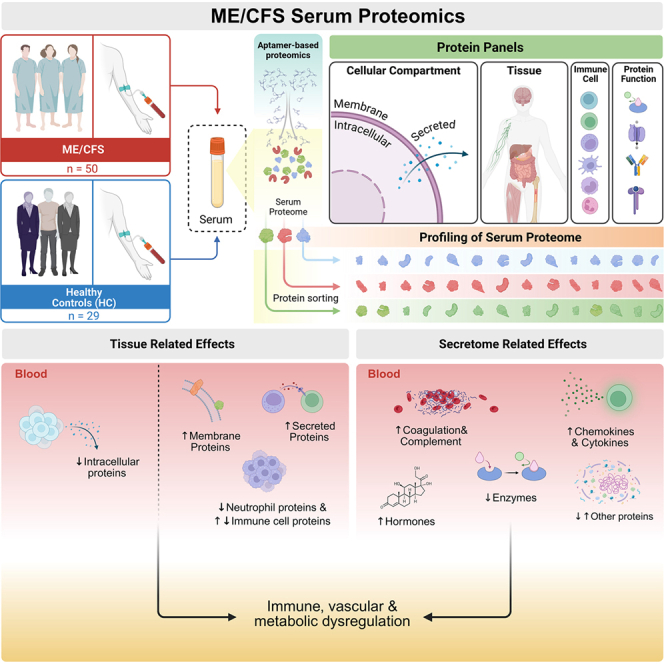

## Teaser

Hoel et al. characterize circulating proteomic signatures in ME/CFS and identify widespread tissue-linked alterations, including increased secreted proteins and reduced intracellular proteins. Protein changes affecting immune, metabolic, and vascular processes support immune reprogramming and define regulatory networks that provide pathobiological insight relevant to biomarker development and future therapeutic strategies.

## Introduction

Myalgic encephalomyelitis/chronic fatigue syndrome (ME/CFS) is a debilitating disease that often begins abruptly following an infection and is increasingly recognized as part of the broader category of post-acute infection syndromes.^1^^,^^2^^,^^3^^,^^4^^,^^5^ Using the Canadian consensus criteria,^6^ the pre-COVID-19 pandemic prevalence of ME/CFS was estimated to be between 0.2% and 0.8%.^7^^,^^8^^,^^9^ Key symptoms include profound fatigue, post-exertional malaise (PEM), sensory hypersensitivity, pain, unrefreshing sleep, and cognitive dysfunction.^10^^,^^11^ The consequences of ME/CFS are severe for both patients and their families, with high socio-economic costs.^9^^,^^12^^,^^13^^,^^14^ There is an urgent need for more knowledge about the biological mechanisms of ME/CFS to develop effective diagnostic markers and treatments.

Although the etiology of ME/CFS remains unclear, possible roles of immune system dysregulation, chronic inflammation, and impaired metabolism, have been suggested based on research findings.^1^^,^^15^ We are currently investigating the hypothesis that ME/CFS may involve an autoimmune mechanism leading to vascular dysregulation, causing tissue hypoperfusion and hypoxia, especially upon exertion, resulting in both short- and long-term effects on energy metabolism.^16^^,^^17^ This mechanism could explain key aspects of symptom generation and fatiguability in ME/CFS, potentially involving exertion-triggered muscle abnormalities, as recently reported in association with PEM in long COVID patients.^18^

Human cells express thousands of proteins in tissue-specific patterns,^19^^,^^20^ many of which enter the bloodstream either secreted as functional messengers or through tissue leakage.^21^ Disease processes alter both protein expression and release, shaping the circulating proteome and offering insights into tissue homeostasis, secretory regulation, and metabolism.^20^ Previous ME/CFS studies have reported variable abnormalities in cytokines and immune factors,^22^^,^^23^ vascular regulators,^24^ and metabolic or muscle-derived messengers,^17^^,^^25^ highlighting the need for deeper proteomic investigation.

Aptamer microarray proteomics enables high-throughput measurement of thousands of circulating proteins from small sample volumes using protein-specific DNA/RNA aptamers.^26^ This platform has been increasingly applied to studies of human disease, including aging, kidney disease, and diabetes,^27^^,^^28^ as well as in two previous ME/CFS studies involving relatively small cohorts.^29^^,^^30^ Here, we used this approach to map serum protein patterns in ME/CFS, aiming to reveal coordinated cellular, tissue-level, and systemic processes, with particular attention to immune-related mechanisms that may underlie vascular and metabolic dysfunction.

## Results

### Characterization of the subjects

**Figure 1:**
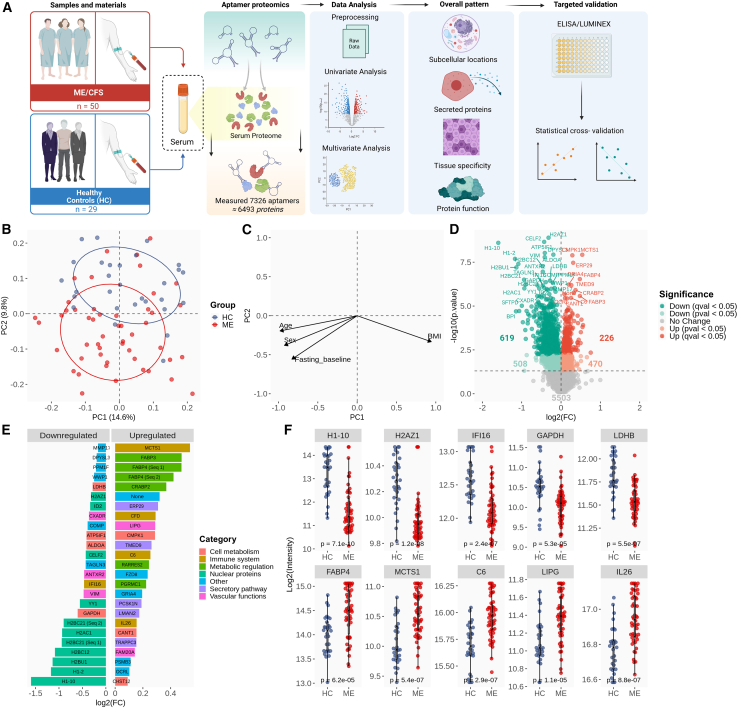
Serum proteomics comparing ME/CFS (ME) patients with HC (A) Study approach: Serum protein concentrations for 50 ME/CFS patients and 29 HCs were measured using aptamer-based technology (SomaScan v.4.1 7 k). Cross-system profiling was conducted to characterize cellular and tissue-specific impacts, effects on the secretome, and the associated molecular and biological functions involved. Antibody-based measurements were used to validate and expand on findings, with a focus on the immune system and energy metabolism. (B and C) (B) PCA with overlay of the ME/CFS and HC groups; (C) projections of associated eigenvalues reflecting the influence of age, sex, BMI, and fasting on the PC1 and PC2 dimensions. (D) Volcano plot displaying all the *p* and q significant features. The dotted line indicates −log10(0.05), i.e., the *p* value significance threshold. (E) Aptamers were grouped by regulation direction (logFC >0 or <0). The 25 most significant aptamers per group (ranked by *p* value) are shown, ordered by log_2_(FC) and color coded by the established biological roles of their protein targets. Seq1 and Seq2 refer to two different aptamers targeting the same protein (FABP4 in this case). “None” means that the protein has not yet been assigned with a gene symbol in the NCBI Entrez Gene database. (F) Group comparison of selected highly affected serum proteins (median ±1.5×IQR [interquartile range]). Nominal *p* values are displayed for each comparison (Welch’s *t* test). The statistical outcomes of the study included both uncorrected (*p* value) and multiple comparison-corrected (FDR, q value) univariate analyses, adjusted for sex, age, BMI, and overnight fasting.

The curated dataset included 50 serum samples from ME/CFS patients collected at baseline in two clinical intervention trials (RituxME, NCT02229942, 2014–2017,^31^ and CycloME, NCT02444091, 2015–2020^32^), along with 29 sex- and age-matched healthy controls (HCs) (STAR Methods; Table 1; Figure 1A). Eleven patients had fasted overnight prior to sampling. Blood glucose levels did not differ between groups (Table 1), but triacylglycerols and non-esterified fatty acids were higher in ME/CFS. This is consistent with our earlier metabolomics study of samples from the same biobank, which identified three metabolic phenotypes (metabotypes): M1 (more lipolytic/ketogenic), M2 (more lipid accumulation), and M3 (metabolically similar to controls).^17^ These metabotypes showed only minor influence from sex, BMI, age, medication, or fasting, but possible associations with physical impairment and disease severity. In the present study, we applied these metabotypes to explore links between serum proteomic and metabolomic alterations in ME/CFS.

**Table 1:** Group characteristics

Study groups	HC	ME	*p* value
Subjects, *n* (%)	29 (100)	50 (100)	–
Women, *n* (%)	21 (72)	40 (80)	–
Men, *n* (%)	8 (28)	10 (20)	–
RituxME, *n*	N/A	15	–
CycloME, response, *n*	N/A	21	–
CycloME, no response, *n*	N/A	14	–
Fasting, *n* (%)	0 (0)	11 (22)	–
BMI (mean ± SD)	24.0 ± 2.6^b^	24.4 ± 4.2	0.6495
Age	38.4 ± 9.3	40.4 ± 10.1	0.3615
Infection, *n* (%)	N/A	38 (76)	–
Mean steps 24 h (mean ± SD)	N/A	3,021 ± 2,001	–
SF-36PF (mean ± SD)	N/A	31.2 ± 19.3	–
Self-reported PF (%, mean ± SD)	N/A	16.6 ± 7.7	–
Glucose (mM, mean ± SD)	5.04 ± 0.40	5.24 ± 0.82	0.3828
TAG (mM, mean ± SD)	0.87 ± 0.31	1.35 ± 0.89	0.0254^a^
NEFA (mM, mean ± SD)	0.21 ± 0.13	0.40 ± 0.28	0.0262^a^

Overview of the two groups of HC subjects and ME/CFS patients. The ME/CFS samples were obtained from participants in the RituxME and CycloME trials. Measurements of glucose, TAG, and NEFA were performed in the clinic.

N/A, not applicable; NEFA, non-esterified fatty acid; self-reported PF, self-reported physical function; SF-36PF, SF-36 physical functioning; TAG, triacylglycerol.

a *p* < 0.05, Welch’s test.

b HC BMI *n* = 24.

### Comprehensive differences between the ME/CFS group and the HC group

The dataset comprised 7,326 aptamers that collectively recognized 6,493 protein *targets* listed by “TargetFullName,” with some aptamers binding to the same target (Supplemental Data S1). These corresponded to 6,408 *proteins* identified by their “EntrezGeneSymbol,” because several targets represented different isoforms, fragments, or other proteoforms of the same gene product (Supplemental Data S1). For the 786 proteins recognized by two or more aptamers, we examined both signal consistency (Pearson correlation) and group-level directional concordance (Supplemental Data S1; Document S1: Methods S1B, Table S2). Overall, 41.6% of proteins showed moderate to strong correlations (|R| > 0.3), while 58.4% showed weak or none (|R| < 0.3), consistent with proteoform- or domain-specific binding effects. Despite these molecular variations, 97.8% of the aptamer pairs showing statistical significance (*p* < 0.05) displayed directional concordance for the differences between ME/CFS and HC groups, supporting the robustness of the group-level findings.

Although the PCA (principal-component [PC] analysis) plot showed overlap between the ME/CFS and HC groups, PERMANOVA confirmed a global group-level separation (F = 14.0, R^2^ = 0.15, *p* = 0.001), which was primarily due to differences along the PC2 dimension (*y* axis, 9.6% explained variance) (Figures 1B and 1C; Supplemental Data S2). Thus, the overlap along the PC1 dimension (*x* axis; 14.5% explained variance) primarily reflected intragroup protein variation shared between ME/CFS and control samples. This makes sense, as PC1 was more influenced by the covariate factors, age, sex, BMI, and fasting state, as indicated by the respective loadings, compared to PC2 (Figure 1C; variable, PC1, PC2, *p* of the correlation; BMI, 0.943, −0.333, 0.106; age, −0.981, −0.195, 0.381; sex, −0.925, −0.380, 0.091; fasting, −0.830, −0.557, 0.046). Therefore, we concluded that group separation along PC2 was not primarily driven by the covariates age, sex, BMI, and fasting state, but rather may reflect disease-specific changes. Accordingly, the major PC2-driving proteins (i.e., with high loadings) showed more statistically significant differences between the two groups, compared with major PC1-driving proteins. Additional analyses aimed at distinguishing disease-specific effects from covariate influences were included in the subsequent investigations.

A total of 1,823 aptamers differed significantly between ME/CFS and HC groups (*p* < 0.05), with 845 remaining significant after false discovery rate (FDR) correction (q < 0.05) (Supplemental Data S3). After merging duplicated targets according to a consistent rule set (Document S1: Methods S1B), 1,723 unique proteins were identified as altered, including 811 significant after FDR correction (Document S1: Data S1A, Table S3). More than 60% of the altered aptamers showed lower abundance of their protein targets in ME/CFS (61.8% at *p* < 0.05; 73.3% at q < 0.05), while the remainder exhibited higher levels (38.2% using *p* < 0.05; 26.7% at q < 0.05) (Figure 1D). To avoid the risk of missing biologically meaningful patterns, given the small effect sizes and intragroup heterogeneity, *p* values were used for exploratory hypothesis-generating investigations, whereas q values were used to indicate robust findings.

### Initial key observations related to the possible pathomechanism

The 25 most altered proteins in the ME/CFS group revealed some patterns that were further supported by the subsequent analyses: intracellular proteins, including histones and metabolic enzymes, were predominantly reduced, whereas secretory proteins involved in immune regulation, systemic metabolism, and vascular function were frequently elevated (Figure 1E). Individual examples of highly affected proteins are shown in Figure 1F to illustrate both the magnitude of heterogeneity and the degree of overlap between groups.

Among the proteins showing the lowest levels in ME/CFS were nuclear histones (H1x, H2.1, and several H2B and H2A types) (Figures 1E and 1F), which can be released by activated neutrophils, e.g., as part of neutrophil extracellular traps.^33^ Other proteins showing pronounced lower levels included enzymes related to cellular energy metabolism, such as glyceraldehyde-3-phosphate dehydrogenase (GAPDH), fructose-bisphosphate aldolase A (ALDOA), and L-lactate dehydrogenase B chain (LDHB), and proteins linked to vascular function and hypoxia responses, including vimentin (VIM),^34^ anthrax toxin receptor 2 ([ANTXR2], also known as capillary morphogenesis gene 2),^35^ coxsackievirus and adenovirus receptor (CXADR),^36^ and ATP synthase inhibitory factor subunit 1 (ATP5IF1).^37^ Additional decreases were observed for the neuronal protein transgelin-3 (TAGLN3) and cartilage oligomeric matrix protein (COMP).

Conversely, several immune-related proteins were among the most highly elevated, including malignant T-cell-amplified sequence 1 (MCTS1),^38^^,^^39^ complement factor D (CFD), complement component C6 (C6), and the cytokine interleukin (IL)-26 (Figures 1E and 1F). There were also increases in metabolic stress-associated proteins, such as fatty acid-binding proteins 3 and 4 (FABP3 and FABP4).^40^ Furthermore, increases were seen for cellular retinoic acid-binding protein 2 (CRABP2), a member of the fatty acid-binding protein family; the adipokine and immune cell chemoattractant retinoic acid responder protein 2 ([RARRES2]; chemerin)^41^; and endothelial cell-derived lipase (LIPG), which is involved in lipoprotein metabolism and vascular biology^42^ as well as immune activation.^43^ There were also higher levels of endoplasmic reticulum resident protein 29 (ERP29); transmembrane emp24 domain-containing protein 9 (TMED9); pseudokinase FAM20A, which may function in hematopoiesis; and soluble calcium-activated nucleotidase 1 (CANT1).

Univariate logistic regression analyses highlighted aptamers such as ALDOA, DPYSL3, H1-10, and ATP5IF1 with areas under the curve around 0.87 and balanced sensitivity (∼0.78–0.88) and specificity (∼0.79–0.90) (Supplemental Data S3), although these remain exploratory without external validation. Although the proteomic profiles of the 21 cyclophosphamide responders versus 14 non-responders overlapped by PCA (Supplemental Data S3; Document S1: Data S1B, Figure S2; Tables S4 and S5), several immune-related proteins (e.g., IGHE, KIT, CD80, and IL-34) showed nominally significant differences (*p* < 0.05), none of which remained significant after FDR correction.

### Influence by covariates, metabotype, and physical function/activity

**Figure 2:**
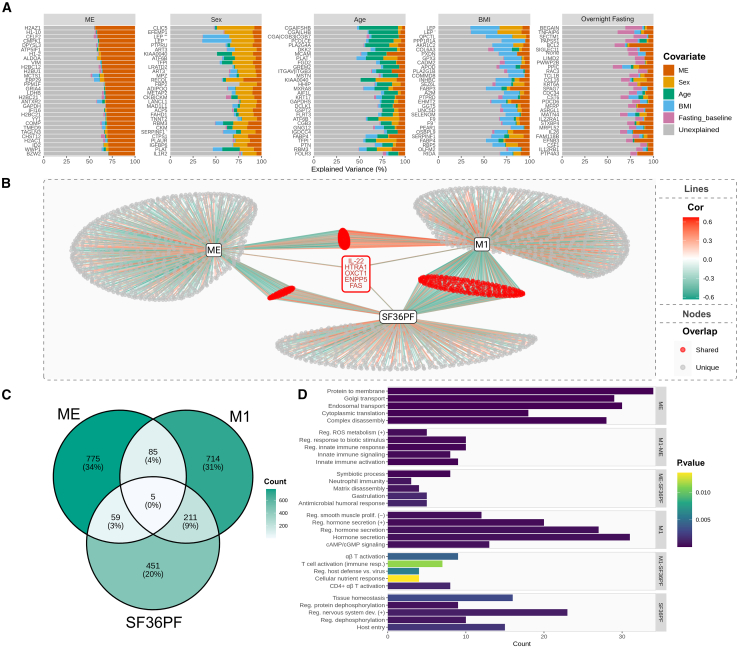
Analysis of influence by covariate factors (A) The proportion of variance explained by each covariate (ME, sex, age, BMI, and fasting state), across the top 30 most explained features per covariate (different colors). (B) Semi-partial Pearson correlation network displaying associations between aptamers and ME/CFS diagnosis (ME), metabotype (represented by metabotype M1), and SF-36PF, adjusted for sex, BMI, overnight fasting, and age. Color of lines (Cor) indicates direction (red, positive; green, negative), and intensity shows the strength of the correlation. (C) Venn diagram summarizing the data in (B). (D) Gene Ontology enrichment analysis of biological processes within network-derived clusters. The *x* axis indicates aptamer counts within category, and bare color shows significance level (*p* value).

We assessed the influence of sex, age, BMI, and fasting on protein levels by comparing the variance of each covariate explained relative to ME/CFS diagnosis (Supplemental Data S2). Figure 2A displays the 30 aptamers showing the highest variance for each covariate. Overall, the aptamers highly influenced by ME/CFS showed relatively small additional influence from the tested covariates, suggesting that these changes largely reflect disease-specific alterations. Several of the aforementioned affected proteins showed such specificity to ME/CFS, such as histones (e.g., H2AZ1 and H1-10), VIM, MCTS1, and LDHB. By contrast, sex, age, BMI, and fasting associated with other, partially overlapping, sets of proteins, e.g., metabolic hormones such as leptin, FABP3, and FABP4, which showed mixed influence patterns.

To further assess potential associations with the metabolic context and physical function, we correlated ME/CFS diagnosis, SF-36 Physical Function (SF-36PF), and metabotype (using metabotype M1 as reference^17^), adjusting for sex, age, BMI, and fasting (Figures 2B–2D; Supplemental Data S2). Of 924 aptamers associated with ME/CFS, 775 were unique to the diagnosis and enriched for intracellular transport/translation. Metabotype and SF-36PF influenced 1,015 and 726 aptamers, respectively, with distinct enrichments: metabotype-linked proteins involved hormone secretion and smooth-muscle cell regulation, while SF-36PF-linked proteins related to tissue homeostasis and nervous system development. A subset of 149 aptamers overlapped with both ME/CFS and either metabotype or SF-36PF, highlighting shared immune-metabolic and extracellular matrix pathways, which may link disease status to metabolic context and clinical severity. Only five proteins showed joint influence by ME/CFS status, SF-36PF, and metabotype (IL-22, HTRA1, OXCT1, ENPP5, and FAS).

Furthermore, a large fraction of aptamers within the SF-36PF cluster also correlated with mean 24-h steps count, a direct measure of physical activity (Supplemental Data S2; Document S1: Data S1C, Figure S3; Table S6). In contrast, very few aptamers within the cluster distinguishing ME/CFS from controls showed such correlations, suggesting that these proteomic patterns are not primarily driven by the lack of activity due to impaired physical function.

Overall, these results indicate that a substantial proportion of the broad proteomic alterations in ME/CFS cannot be explained by demographic factors or deconditioning, but instead may point to intrinsic disease-associated molecular changes.

### Cellular proteins in circulation: Release patterns

Cellular proteins enter the bloodstream through both active secretion and passive release from cell turnover or damage, making biological context essential for interpreting altered protein levels. We applied resources such as the Human Protein Atlas (HPA) and a curated human secretome database^44^ to contextualize the circulating proteome patterns in terms of tissue and cell origin, secretion status, and biological function.

**Figure 3:**
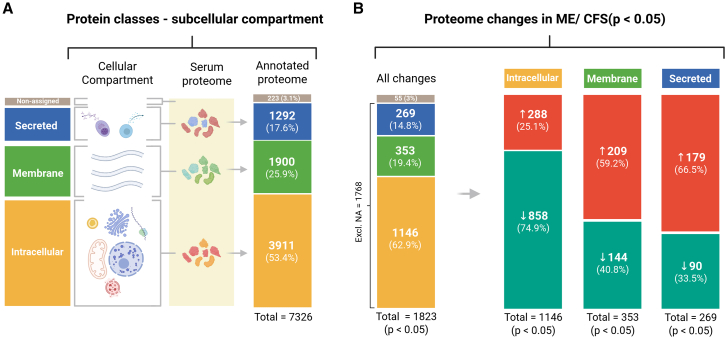
The subcellular origin of the affected proteins The aptamers were classified into four categories based on the annotated subcellular localization of the targeted proteins (HPA): “intracellular,” “membrane” (membrane + membrane-associated), “secreted” proteins, and the remaining unannotated (“non-assigned” [NA], gray bars). The numbers indicate aptamer counts and corresponding percentages of (A) all the aptamers and (B) only the altered aptamers. The percentages in the three subcellular categories (shown on the right) are relative to the total number of assigned aptamers in each class. The red and green bars illustrate the proportions of increases and decreases, respectively.

Based on subcellular compartment annotations for all 7,326 aptamers, 53.4% (*n* = 3,911) targeted intracellular proteins, 25.9% (*n* = 1,900) membrane-associated proteins (membrane + membrane & secreted), and 17.6% (*n* = 1,292) secreted proteins, while 3.0% (*n* = 223) were unannotated (Figure 3A, Supplemental Data S3). Among the 1,823 aptamers showing significant group differences (*p* < 0.05), 1,146 targeted intracellular proteins (Figure 3B), of which 74.9% showed lower serum concentration in ME/CFS, indicating a disproportionate effect on this protein class. Conversely, only 40.8% of altered membrane proteins and 33.5% of altered secreted proteins were reduced, with the remainder showing increased levels. Together, these data indicate a distinct ME/CFS serum pattern characterized by reduced abundance of intracellular proteins (Kolmogorov-Smirnov [KS] test: D = 0.080, *p* = 1.6 × 10^−14^; Hodges-Lehmann estimator [HL] = −0.18 [−0.22 to −0.14]) and increased levels of membrane (KS test: D = 0.089, *p* = 5.3 × 10^−10^, HL = +0.14 [0.10–0.19]) and secreted proteins (KS test: D = 0.123, *p* = 7.7 × 10^−15^; HL = +0.27 [0.22–0.33]), suggesting altered cellular turnover and secretory processes. These relationships were consistent when the comparison was aggregated at the protein level rather than the aptamer level (Document S1: Data S1A, Table S3).

Gene set enrichment analysis of ranked univariate statistics (ME/CFS vs. HC) using GO Biological Process and Reactome terms indicated a broad downregulation of intracellular pathways (Supplemental Data S3). Although this pattern is consistent with the widespread reduction of intracellular protein levels observed in ME/CFS serum, such interpretation may be limited, as these proteins lose their original cellular context once released into the circulation.

Using the MitoCarta 3.0 library,^45^ we identified 425 mitochondrial proteins targeted by 445 aptamers in the dataset (Supplemental Data S3). Of these, 20.7% (92 aptamers) showed lower blood concentrations in ME/CFS compared to HC, whereas only 6.7% (30 aptamers) showed higher levels. This indicates a change toward reduced release of mitochondrial proteins into the blood, mirroring the overall pattern observed for intracellular proteins.

### Organ and immune cell signatures in the serum proteome

**Figure 4:**
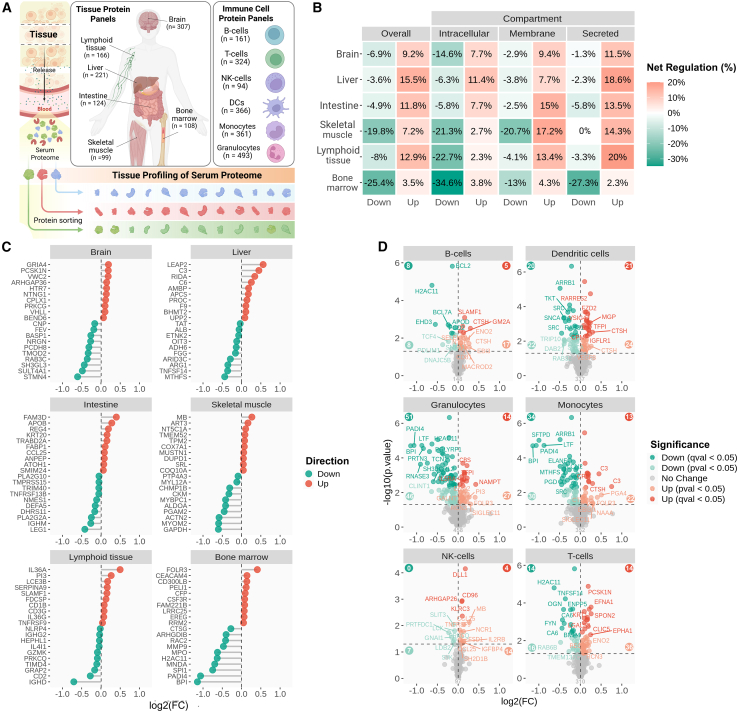
Tissue- and immune cell-associated footprints (A) Schematic illustration showing the approach of using panels of tissue- and immune cell-associated proteins (HPA) to characterize organ-level contributions to the altered serum proteome. The number of proteins in each panel is shown. (B) For each protein panel, the proportion of affected proteins (up/down) was calculated relative (%) to the total number of proteins in the panel, and after further division into cellular compartments. (C) Plots showing the most affected proteins per tissue type, ranked by fold change (log2(FC)). (D) Volcano plots of immune cell type-associated protein panels. The dotted line indicates −log10(0.05), i.e., the *p* value significance threshold.

To assess organ contributions to the circulating proteome (Figure 4A), we applied tissue-associated protein panels (HPA), covering 1,670 aptamers targeting 1,428 proteins identified by their “UniProt,” of which 20.9% (*n* = 299; *p* < 0.05) differed between ME/CFS and HC groups (Supplemental Data S4). Although these panels are not strictly tissue specific, they may still provide meaningful insights into organ-level contributions to the altered serum proteome. Because some proteins were represented in multiple panels, and some were recognized by more than one aptamer, the total number of panel entries exceeded the number of unique proteins. Figure 4B illustrates changes across the protein panels for brain, liver, intestine, skeletal muscle, lymphoid tissue, and bone marrow, which were the most prominent contributors to the altered serum proteome (additional tissues are shown in Supplemental Data S4). In the brain panel, slightly more proteins showed higher rather than lower serum levels in ME/CFS, driven mainly by membrane and secreted proteins, whereas intracellular proteins tended to be reduced. In the liver and intestine panels, a similar pattern was observed, but with less reduction of intracellular proteins. In the skeletal muscle panel, intracellular proteins showed marked reductions, membrane proteins displayed mixed effects, and all affected secreted proteins were increased. In the lymphoid tissue panel, intracellular proteins were reduced, while membrane and secreted proteins were mainly elevated. The bone marrow panel showed a distinct profile, with few increases and widespread reductions, particularly in intracellular and secreted proteins. For the most affected individual proteins, reductions were most pronounced in the panels for brain, skeletal muscle, and bone marrow, whereas liver, intestine, and lymphoid tissues showed comparatively modest, bidirectional alterations (Figure 4C). Overall, these tissue-specific profiles indicate a pattern of reduced intracellular protein release, particularly from skeletal muscle, brain, and bone marrow, accompanied by broadly increased secretion across tissues, suggesting coordinated multi-organ involvement in the pathophysiology of ME/CFS.

Furthermore, to evaluate effects across immune cell types, six immune-cell-associated protein panels were used (HPA: B cells, dendritic cells, granulocytes, monocytes, natural killer [NK] cells, and T cells). The panels included a total of 1,334 aptamers targeting 1,107 proteins identified by “UniProt” (Figure 4A), and also here, some proteins were represented in more than one panel (Figure 4D; Supplemental Data S4). Across the six panels, the overall fraction of affected aptamers ranged from 20.4% to 23.2% (*p* < 0.05; Document S1: Data S1D, Table S7). Notably, granulocytes and monocytes showed the highest proportion of lowered aptamers (70.3% and 64.6% of changes, respectively), whereas NK cells and T cells showed a predominance of elevated aptamers (72.0% and 62.5% of changes, respectively). These findings suggest a primarily innate-immune-skewed reduction in granulocyte- and monocyte-associated signatures, contrasting with more mixed or upward changes in lymphoid cell panels.

To further characterize the observed granulocyte/neutrophil-associated signature, we compared our dataset with a published list of proteins released by phorbol 12-myristate 13-acetate (PMA)-activated neutrophils^33^ (Supplemental Data S3). Of the 254 listed proteins, 146 were detected in our dataset, represented by 172 aptamers, of which 52.3% showed differences between ME/CFS and HC groups (90 aptamers at *p* < 0.05; 62 aptamers, 36.0% at q < 0.05). Notably, more than 85% of the affected aptamers (79 aptamers at *p* < 0.05; 57 aptamers at q < 0.05) showed lower levels in ME/CFS, indicating a broad and pronounced reduction in circulating neutrophil-derived proteins, including several hallmark granule components such as BPI, PADI4, MMP9, ELANE, PRTN3, AZU1, and LTF. This suppressed neutrophil signature was not accompanied by abnormal granulocyte/neutrophil counts in patients (3.38 ± 1.03 × 10^9^/L; normal range 1.5–7.3 × 10^9^/L), and other blood cell counts were also within normal limits (Document S1: Data S1E, Table S8).

### Secretory processes and functional changes

**Figure 5:**
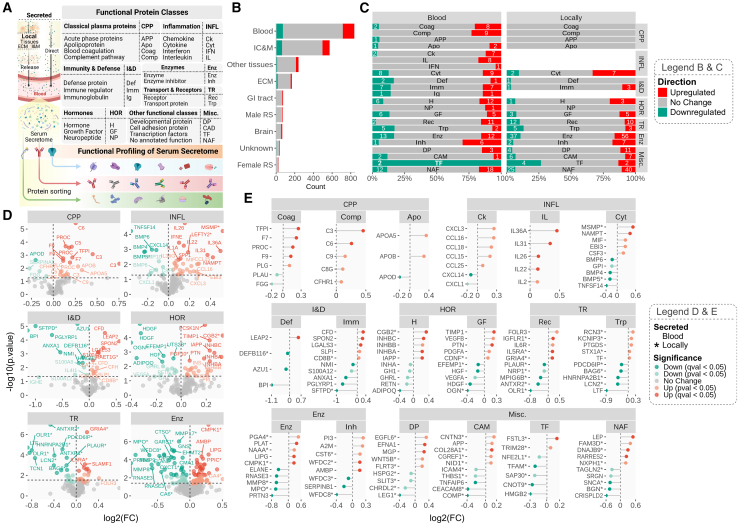
Comparison between the ME/CFS and HC secretome and the related biological processes (A) The framework for secretome analyses. Secreted proteins were classified according to secretion site (blood vs. locally, tissue-specific) and biological function (abbreviations are shown).^44^ (B) The total and significantly affected aptamer counts across proteins secreted to blood or locally within eight tissue compartments (intracellular and membrane proteins, IC&M; extracellular matrix, ECM; male reproductive system, Male RS; female reproductive system, Female RS; unknown origin, Unknown). (C) Distribution and direction of change (up, down, unchanged) across functional protein classes, shown as the percentage of all measured proteins within each class (*x* axis). (D and E) (D) Volcano plots showing all secreted proteins grouped by biological function; colors indicate up- or downregulation and statistical significance. The dotted line indicates −log10(0.05), i.e., the *p* value significance threshold. (E) Plots highlighting the most affected proteins within each biological function, ranked by fold change (log_2_FC).

To further investigate the apparent stimulation of secretory processes in ME/CFS, we utilized an optimized protein-centric database of the predicted human secretome,^44^ which annotates both tissue-specific expression and functional roles of secreted proteins (Figure 5A; Supplemental Data S5). Based on 2,100 aptamers recognizing 1,657 proteins (“UniProt”) of the secretome, 21.6% of the aptamers showed difference between ME/CFS and HC groups (*n* = 454 at *p* < 0.05; *n* = 185, 8.8% at q < 0.05), with the majority showing increased levels (61.2% of changes at *p* < 0.05; 54.6% of changes at q < 0.05). The annotation categorized secreted proteins into nine functional groups, including blood proteins (Blood) and proteins secreted locally in eight compartments: intracellular and membrane proteins (IC&M), other tissues, extracellular matrix (ECM), gastrointestinal tract (GI tract), male reproductive system (Male RS), brain, unknown origin (Unknown), and female reproductive system (Female RS). Figure 5B shows a widespread pattern of increased levels across these secretome categories, supporting elevated activity involving multiple organ systems and physiological processes.

Within the largest class, blood proteins, 197 of the 838 aptamers showed significant changes, with 63% (*n* = 124) at higher levels and 37% (*n* = 73) at lower levels. Many of these circulating proteins originate from the liver, while others are produced by blood cells or more broadly across tissues.^44^ Functional annotation indicated mixed impacts across classical plasma proteins (CPP), enzymes (Enz), hormones (HOR), immunity and defense (I&D), inflammation (INFL), and transport and receptors (TR), as well as their subcategories, which reflects distinct biological processes (Figure 5C). Figure 5D shows all the affected aptamers across secretome categories according to function, whereas Figure 5E provides the most affected proteins per functional (sub)category. These data show a pattern of increased secretion of proteins involved in coagulation, complement pathways, and inflammation (chemokines and ILs). In contrast, the enzyme class predominantly showed lower serum levels, which partly relate to the overall decreased release of intracellular proteins in general (Figure 3) and the observed neutrophil signature (Figure 4). The other secretome classes, including hormones, immunity and defense, transport and receptors, and others, expressed mixed effects.

We performed a ligand-receptor interaction analysis to explore potential signaling relationships reflected in the proteomic data (Document S1: Data S1F, Figure S4). This approach identifies pairs of ligands and receptors whose levels change in a coordinated manner, which may indicate altered intercellular communication. The analysis revealed an over-representation of interactions within the cell adhesion and cytokine-cytokine receptor categories. Among these, four members of the Ephrin subfamily A receptors (EPHA1, EPHA2, EPHA4, and EPHA7) and their ligands (EFNA proteins) showed concordant changes, consistent with previously suggested involvement of EPHA-EFNA signaling in ME/CFS.^29^ Additional examples included concordance of FGFR3 with EFNA ligands, IL6R with CNTF and MPZ, and FLRT3 with UNC5B/UNC5D, whereas FAP and PAM showed negative concordance. While these findings do not establish causality, they highlight signaling pathways that may contribute to metabolic regulation, tissue development and repair, inflammation, or angiogenesis in ME/CFS.

### Validation and expansion of serum proteome findings using antibody-based methods

**Figure 6:**
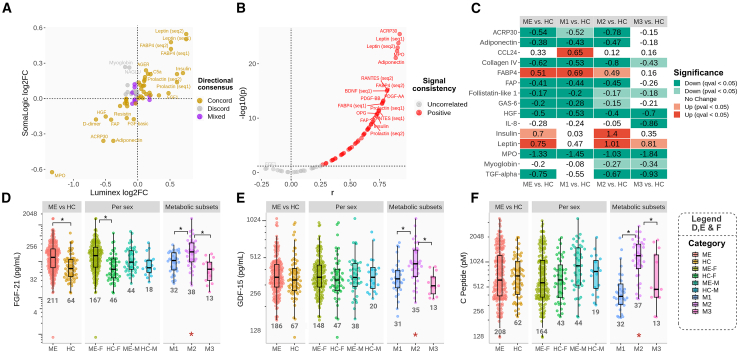
Targeted validation and expansion of key findings Antibody-based measurements (Luminex and ELISA) were used to validate and expand on key outcomes from aptamer-based serum proteomics. Luminex data are shown for a panel of 54 serum proteins related to immunity and metabolism, in 83 ME/CFS patients and 29 HC subjects. Samples were analyzed in single assay wells, with intra- and inter-plate technical replicates included for designated samples. (A and B) (A) The directional concordance, and (B) signal consistency (Pearson correlation), between antibody-based and the aptamer-based measurements, per protein. (C–F) (C) The heatmap displays selected affected proteins, including comparison between the three ME/CFS metabotypes (M1, M2, and M3). Expanded ELISA analysis of (D) FGF-21, (E) GDF-15, and (F) C-peptide was performed in larger cohorts (*n* = 212 ME/CFS, *n* = 66 HC). Each sample was assayed in technical duplicate. Boxplot indicates median and IQR (25^th^–75^th^ percentiles), and the whiskers indicate ±1.5×IQR. Statistical comparisons were performed between groups (Welch’s *t* test with FDR correction, q < 0.05, black asterisks) and stratified by sex and metabotype (one-way ANOVA, *p* < 0.05, red asterisks, followed by post hoc Welch’s *t* test with FDR correction, q < 0.05, black asterisks).

To validate and extend the aptamer-based findings, we measured a panel of serum proteins related to immunity, inflammation, coagulation, and energy-stress metabolism using antibody-based assays (ELISA and Luminex). Samples from 83 ME/CFS patients and 29 HCs (Document S1: Data S1G, Table S9) were analyzed for 77 proteins using the Luminex platform. After excluding 23 proteins with low abundance (defined as >50% missing data), 54 proteins were retained for statistical analysis (Supplemental Data S6). Group-level concordance analysis compared the ME/CFS-associated changes in these 54 Luminex proteins with the 68 corresponding aptamers on the SomaScan platform (Figure 6A; Supplemental Data S6; Document S1, Data S1H). The majority of proteins (70.4%) showed concordant effects across the two platforms; 7.4% showed mixed results because different aptamers targeting the same protein reported divergent changes (notably BDNF, COL1A1, OSM, and osteoactivin) and 22.2% showed discordant results. For signal-consistency analysis, 69.1% of platform comparisons demonstrated a significant positive correlation (*p* < 0.05) and none showed negative correlation (Figure 6B; Supplemental Data S6). In summary, most significant group-level differences between ME/CFS and HC groups were reproduced using antibody-based measurements, thereby supporting the overall findings obtained with aptamer-based detection.

A selection of significant differences between ME/CFS and HC groups based on the Luminex data is shown in the heatmap in Figure 6C, which also compares the serum protein levels across metabotypes. Corresponding data for all the measured proteins are provided in Supplemental Data S6. Metabotype-specific differences were shown particularly for metabolic hormones, such as the high FABP4 level seen in both metabotypes 1 and 2 and the high insulin and leptin levels in metabotype 2. The group-level effects on immune system-related proteins generally remained relatively similar between the metabotype subgroups, such as reduced MPO and TGFα. Two exceptions were CCL24, which expressed a specific increase in the metabotype 1 subgroup, and IL-8, which specifically showed a lower level in the metabotype 3 subgroup.

We performed additional conventional ELISA measurements of two metabolic stress hormones, fibroblast growth factor (FGF)-21 and growth differentiation factor (GDF)-15, in addition to C-peptide, a marker of insulin production, using an extended cohort (*n* = 212 ME/CFS, *n* = 66 HC) (Figures 6D–6F). FGF-21 and C-peptide were not included in the previous aptamer-based analysis. For FGF-21, the serum concentration was higher in the ME/CFS group compared to HC (Figure 6D). When stratified by sex, this difference was statistically significant in women but not in men. The FGF-21 level differed across the three ME/CFS metabotype subsets, with metabotype 2 showing the highest concentration. For GDF-15 and C-peptide, the ELISA analysis showed no overall difference between ME/CFS and HC groups, irrespective of sex (Figures 6E and 6F); however, both markers were higher in metabotype 2 compared to metabotypes 1 and 3.

## Discussion

**Figure 7:**
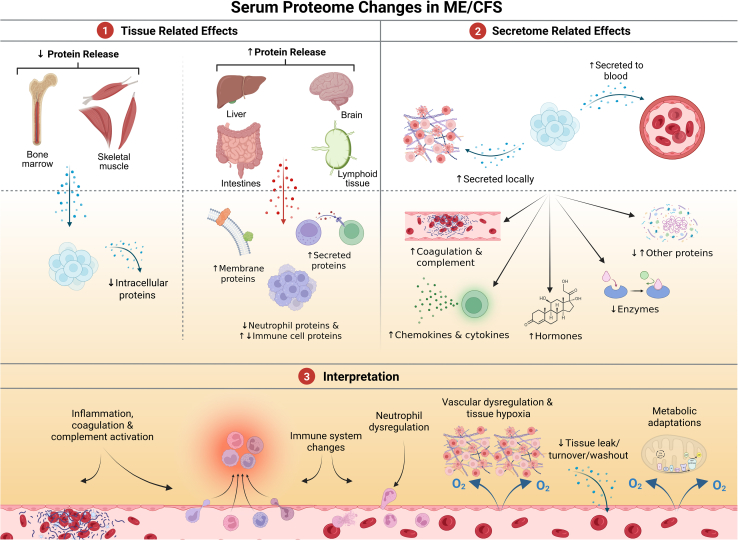
Impacts of serum proteome changes in ME/CFS This schematic summarizes key findings: (1) *Tissue-related effects*: ME/CFS patients showed reduced blood levels of intracellular proteins, most pronounced for skeletal muscle and bone marrow, alongside increased levels of membrane-associated and secreted proteins from brain, intestine, liver, skeletal muscle, and lymphoid tissues. A substantial reduction of proteins released by activated neutrophils contrasted with more variable or elevated lymphoid-cell patterns (2) *Secretome effects*: Overall secretory activity was elevated, with increased levels of coagulation proteins, complement factors, chemokines, cytokines, and hormones, while enzyme levels were reduced. (3) *Interpretation*: These patterns suggest underlying immune dysregulation, possibly involving autoimmune mechanisms, with downstream roles of low-grade inflammation, tissue hypoperfusion, and metabolic disturbance.

This serum proteomics study identified contextual differences in circulatory protein concentrations between ME/CFS patients and healthy individuals. Through the investigations, we aimed to position the observed patterns within the mechanistic landscape, as summarized in Figure 7.

We applied two complementary strategies to gain multiscale insights into ME/CFS pathology. First, we interrogated the full serum proteome to reveal systemic, tissue-associated, and immune-cell-related alterations. Second, we focused specifically on secreted proteins to examine the effects on functional and regulatory physiological processes. Analyses confirmed that sex, age, BMI, and fasting status did not account for the broad proteomic differences between ME/CFS and HC groups. Moreover, the extensive changes found to be unrelated to physical function and activity (SF-36PF, mean steps) argue against deconditioning as the primary driver of the ME/CFS proteome. Rather, it may be relevant to elucidate proteins linking exertion-triggered effects to immunological and metabolic dysregulation, which may potentially track with hallmark symptoms of ME/CFS, such as fatigue and PEM.^46^

### Comparison with other proteomic studies

To compare our results with previous work using the same SomaScan platform, we reanalyzed data from Germain et al.^29^^,^ and Walitt et al.^29^^,^^30^ using a similar analytical pipeline adapted to the available data (STAR Methods; Supplemental Data S7; Document S1: Data S1I, Figures S5 and S6; Tables S10–S13). In the Germain dataset, 391 aptamers differed between ME/CFS and controls (*p* < 0.05), of which 122 overlapped with our findings. In the Walitt dataset, 41 aptamers were altered, with seven overlapping with our data. While neither dataset demonstrated the broad reduction in intracellular proteins observed here, the Germain data showed a relative increase in membrane and secreted proteins, consistent with our results. Despite some inter-study variation, likely reflecting differences in ME/CFS case definitions and cohort size, all three studies consistently pointed to immune-related dysregulation. Our results also align with other independent proteomic investigations showing immune-vascular dysregulation and metabolic involvement, including coagulation and complement pathway changes,^47^ immune-related differences in plasma and extracellular vesicles,^48^ mitochondrial and metabolic pathway changes in immune cells,^49^^,^^50^ and aberrant innate/adaptive immune regulation revealed by single-cell and multi-omics analyses.^51^ Together, these cross-platform findings converge on immune, vascular, and metabolic dysregulation in ME/CFS and strengthen our interpretation of the serum proteome results.

### Reduced protein release from muscle

The reduced release of intracellular skeletal muscle proteins appears to represent a distinct ME/CFS phenotype that is supported by our data. This is unlikely to reflect potential dilution effects from altered blood volume,^52^ given the tissue- and pathway-specific nature of the overall changes. Although decreased leakage of muscle proteins could theoretically result from low physical activity or reduced muscle mass, this explanation is not well supported, as the analyses were adjusted for age, BMI, and sex, which partly account for such effects. While the underlying mechanisms remain unclear, it seems likely that muscle tissue homeostasis and turnover are affected, potentially linked to previously reported muscle abnormalities and contributing to hallmark ME/CFS symptoms such as fatigue and PEM.^18^^,^^53^^,^^54^^,^^55^

### Secretome effects and functional impact

We observed an overall increase of secretory activity in ME/CFS patients, with 62% of affected secreted proteins showing elevated levels. In particular, the affected proteins indicate changes related to coagulation, complement activation, chemokines, ILs, hormones, enzyme inhibitors, and receptors, consistent with previous reports of elevated inflammatory cytokines in ME/CFS, although some variation exists across studies.^22^^,^^56^^,^^57^ We also noted increases in tissue-derived stress messengers such as FABP types, which function intracellularly as fatty acid transporters but act in serum as markers of metabolic stress.^58^ Taken together, the pattern of heightened systemic secretory activity aligns with a chronic state of immune, vascular, and metabolic dysregulation.

### Immune system changes and impaired neutrophil function

Our findings indicate a primarily innate-immune-skewed reduction in granulocyte- and monocyte-associated signatures, contrasting with more variable or elevated lymphoid-cell patterns. This imbalance may reflect a disturbed immune homeostasis that could contribute to or parallel autoimmune-like mechanisms, although the present data do not directly demonstrate autoimmunity. Increased pro-inflammatory cytokines associated with T cell dysregulation, together with mixed changes reflecting disturbed immune balance, are hallmarks of many autoimmune conditions.^59^ The increased inflammatory, coagulation, and complement factors align with previous observations in ME/CFS and long COVID.^1^^,^^60^^,^^61^ The substantial reduction of neutrophil-associated proteins may indicate impaired neutrophil maturation or activation.^62^^,^^63^ Elevated complement factors CFD and C6 indicate alternative complement pathway activation, implicated in vasculitis, lupus, dermatomyositis, and autoimmune nephritis.^64^ Reduced MPO and extracellular histones, which are autoantigens in antineutrophil cytoplasmic antibodies (ANCA)-associated vasculitis, further suggest aberrant neutrophil-complement interplay, although ANCA is not typically reported in ME/CFS.^65^ Additional evidence includes the presence of autoantibodies and promising studies of antibody-targeting therapies such as immune adsorption and plasma-cell-directed treatments.^16^^,^^66^ Collectively, these findings support a possible autoimmune contribution to ME/CFS pathology.

### Vascular dysregulation and hypoxia response

Several proteins linked to microcirculatory and hypoxia responses were altered in ME/CFS. Reduced levels of VIM, ANTXR2, CXADR, and ATP5IF1 may indicate impaired endothelial regulation and mitochondrial stress protection during oxygen deprivation.^34^^,^^35^^,^^36^^,^^37^ In contrast, the endothelial lipase LIPG, known to be induced by immune activation,^42^^,^^43^ and the hematopoietic signaling factor FAM20A^67^ were increased. Endothelial dysfunction, repeatedly reported in ME/CFS,^68^^,^^69^^,^^70^ can impair vascular tone, oxygen delivery, immune interactions, and metabolism through disrupted microcirculatory signaling.^71^^,^^72^^,^^73^ Similar capillary alterations and hypoxia-related changes are described in long COVID,^74^^,^^75^^,^^76^ and ME/CFS patients showed increased WASF3 expression, a target of the hypoxia-regulated transcription factor HIF1A.^77^ Together, these findings support a model in which endothelial dysfunction, impaired vascular flow, and tissue hypoxia contribute to the pathophysiology of ME/CFS.

### Metabolic stress and energy metabolism

Our results support prior evidence of metabolic stress signaling in context of ME/CFS, including elevated GDF-15,^78^ FGF-21, resistin, leptin, and hepatocyte growth factor (HGF).^22^^,^^56^ FGF-21 and GDF-15, which are stress hormones also elevated in mitochondrial myopathies, type 2 diabetes, and hypoxia, were particularly increased in the previously defined metabotype 2 subgroup, alongside higher C-peptide and leptin, while FABP4 was elevated in both metabotypes 1 and 2.^17^ These so-called exerkines (e.g., FGF-21 and GDF-15) normally rise transiently during exercise to coordinate systemic energy adaptation.^79^ Their chronic elevation in ME/CFS suggests persistent energy strain, which could reflect or exacerbate impaired oxygen delivery and utilization,^80^ as supported by findings in ME/CFS,^81^ long COVID,^82^ and metabolic myopathies,^83^ and may contribute to PEM and reduced exercise tolerance.

### Perspectives

There are several ways in which we believe this work could support new research directions. First, it identifies specific cells, organs, and processes affected in ME/CFS, which may guide the development of diagnostic markers and therapeutic strategies. These insights may also be relevant to related syndromes, such as long COVID and fibromyalgia. Second, it provides a mechanistic landscape that can be used to contextualize previous and future findings within a broader (patho)physiological framework, e.g., in relation to potential risk factors identified through multi-omics analyses.^84^^,^^85^ Third, it may inform the development of relevant laboratory models for ME/CFS, including through systemic comparisons^86^ and *in vitro* recapitulation of key mechanistic features.^87^

In conclusion, the serum proteome in ME/CFS reveals a distinct pattern characterized by reduced release of intracellular proteins and increased secretion of extracellular proteins, indicating widespread tissue involvement and disturbed systemic homeostasis. These changes point to altered metabolic and inflammatory regulation across multiple organs, including skeletal muscle, brain, and bone marrow. The accompanying immune dysregulation and attenuated neutrophil function, further supports the notion of a sustained, multisystem imbalance involving immune, vascular, and metabolic processes. Together, these findings offer molecular insight into ME/CFS pathophysiology and a foundation for future biomarker and therapeutic development.

### Limitations of the study

Aptamer-based proteomics offers broad, sensitive, and highly multiplex profiling that is relatively robust to protein degradation and pre-analytical variation,^88^ although it remains semi-quantitative and provides lower resolution than mass spectrometry or antibody-based platforms for detecting isoforms, proteoforms, or post-translational modifications.^89^ Therefore, findings of interest should be verified using additional high-confidence methods. The sample size was primarily limited by the availability of well-characterized biobanked serum samples from two clinical trials and the high cost of large-scale SomaScan 7k profiling. While modest in size, this study is comparable in size to other studies in the field, providing a basis for interpretation. Future studies in larger, independent cohorts will be important to replicate and extend these findings. When using protein annotations to track functions and tissue locations, it is important to be aware that few proteins are exclusively specific to a given process or organ and may play different context-dependent roles. Therefore, alternative interpretations of the findings may be relevant. We have mitigated some of these limitations by primarily focusing our interpretations on patterns of changes related to different tissues and processes, rather than single protein effects.

## Resource availability

### Lead contact

Further information and requests for resources and reagents should be directed to and will be fulfilled by the lead contact, Karl Johan Tronstad (karl.tronstad@uib.no).

### Materials availability

This study did not generate new unique reagents.

### Data and code availability

• The analyzed proteomics data are included as supplemental information. The original dataset is deposited in the PRIDE^90^ repository (PRIDE: PAD000026).
• Any additional information required to reanalyze the data reported in this paper is available from the lead contact upon request.
• This paper does not generate original code.

## Acknowledgments

The authors thank the study personnel at each center of the “RituxME” and “CycloME” clinical trials for their efforts in patient follow-up and biobank sampling. This work received financial support from 10.13039/100019647The Kavli Trust, the 10.13039/501100005416Research Council of Norway (KJT, projects 272680 and 343168), the 10.13039/501100004257Western Norway Regional Health Authority (Helse Vest), and the 10.13039/501100005036University of Bergen (PhD grants to F.H. and A.H.). The clinical trials that recruited patients for the biobank and the laboratory analyses in this study were financially supported by the 10.13039/501100005416Research Council of Norway, the Norwegian Regional Health Trusts, the MEandYou Foundation, the Norwegian ME Association, and the legacy of Torstein Hereid. Additionally, we are grateful for the support from private donors and the Norwegian ME Association for laboratory research on 10.13039/100006598ME/10.13039/100015101CFS.

## Author contributions

A.H., F.H., H.C., Ø.F., O.M., and K.J.T. designed the analytical approach. Ø.F., O.M., I.G.R., K.S., K.R., and K.A. included patients in clinical studies and provided biobank samples and data. F.H., S.E.D., and A.H. performed laboratory measurements. K.A.B. and H.-P.M. provided scientific and technical advice. A.H., F.H., H.C., K.J.T., and Ø.F. conducted data analyses. A.H., F.H., H.C., and K.J.T. wrote the first version of manuscript. All authors approved the final version of the manuscript.

## Declaration of interests

The authors have declared that no conflict of interest exists related to this work.

## Declaration of generative AI and AI-assisted technologies in the writing process

During the preparation of this manuscript, the authors used Microsoft Copilot (UiB institutional license) and ChatGPT5 to assist with translation and ensure readability. After using this tool/service, the authors reviewed and edited the content as needed and take full responsibility for the content of the published article.

## STAR★Methods

### Key resources table

**Table:** 

REAGENT or RESOURCE	SOURCE	IDENTIFIER
**Critical commerical assays**		

Custom Luminex Human Discovery assay	R&D Systems	Cat# LXSAHM; RRID:AB_2924693
Quantikine Ready-To-Use ELISA kit (FGF-21)	R&D Systems	Cat# DF2100; RRID:AB_2783729
Quantikine Ready-To-Use ELISA kit (GDF-15)	R&D Systems	Cat# DGD150; RRID:AB_2877710
Quantikine Ready-To-Use ELISA kit (C-peptide)	R&D Systems	Cat# DICP00
SomaScan Discovery, 7 K	SomaLogic Inc	v. 4.1

**Biological samples**		

Human serum samples	ME/CFS Biobank, Haukeland University Hospital, Norway	RituxME: NCT02229942 CycloME: NCT02444091

**Deposited data**		

Proteomics dataset	Deposited in the PRIDE^90^ repository (PRIDE: PAD000026)	N/A
Metabolomics data	Hoel et al.^17^; DOI: https://doi.org/10.1172/jci.insight.149217	N/A

**Software and algorithms**		

GraphPad Prism 9	GraphPad Software	N/A
R (4.5.1)	https://www.r-project.org	N/A
Rstudio (2025.09.1 + 401)	Posit Software	N/A
R package: Vegan (2.7.0)	DOI: https://doi.org/10.32614/CRAN.package.vegan	N/A
R package: limma (3.64.3)	DOI: https://doi.org/10.18129/B9.bioc.limma	N/A
R package: variancePartition (1.38.1)	DOI: https://doi.org/10.18129/B9.bioc.variancePartition	N/A
R package: ppcor (1.1)	DOI: https://doi.org/10.32614/CRAN.package.ppcor	N/A
R package: clusterProfiler (4.16.0)	DOI: https://doi.org/10.18129/B9.bioc.clusterProfiler	N/A
R package: ComplexHeatmap (2.24.1)	DOI: https://doi.org/10.18129/B9.bioc.ComplexHeatmap	N/A
R package: ggplot2 (4.0.0)	DOI: https://doi.org/10.32614/CRAN.package.ggplot2	N/A
R package: tidygraph (1.3.1)	DOI: https://doi.org/10.32614/CRAN.package.tidygraph	N/A
R package: igraph (2.2.0)	DOI: https://doi.org/10.32614/CRAN.package.igraph	N/A
R package: ggpubr (0.6.2)	DOI: https://doi.org/10.32614/CRAN.package.ggpubr	N/A
Cellinker	DOI: https://doi.org/10.1093/bioinformatics/btab036	N/A
BioRender	BioRender	N/A
Microsoft Office Excel	Microsoft	N/A

Other		

Human protein atlas (HPA)	https://www.proteinatlas.org/	N/A

### Experimental model and study participant details

#### Human participants

This study analyzed serum samples from participants enrolled in the RituxME^31^ and CycloME^32^ clinical trials, as well as healthy control volunteers. Participants were allocated to ME/CFS or healthy control (HC) groups based on clinical status (ME/CFS diagnosis using Canadian criteria^6^ vs. HC). Healthy controls were age- and sex-matched. Within the eligible pool of samples, individuals were randomly selected to minimize selection bias. Serum samples were stored at −80°C.

In total, 54 ME/CFS and 29 HC participants were analyzed on the aptamer-platform. Age, sex, BMI, and fasting status were recorded for all participants. Sex was included as a covariate in statistical models, and sex-stratified analyses were performed for selected targets where feasible; however, the study was not powered to systematically evaluate sex-specific effects across all proteomic features. Eleven ME/CFS subjects who performed overnight fasting before the sampling were included to facilitate evaluation of the impact of fasting as a covariate factor. After preprocessing and outlier removal, 50 ME/CFS and 29 HC samples remained for analysis (see below, and DocumentS1: Methods S1A, Table S1). For covariate-adjusted univariate testing, 50 ME/CFS and 24 HC samples were retained; for metabotype^17^ analyses, 40 ME/CFS subjects, all but one non-fasting, and 24 non-fasting HC subjects were included.

In the validation measurements (Luminex and ELISA), samples were randomly picked from the ME/CFS biobank, including a total of 212 ME/CFS patients and 66 healthy individuals.

#### Ethics statement

All studies were conducted in accordance with the Declaration of Helsinki and approved by the Regional Committee for Medical and Health Research Ethics, Western Norway (REK Vest; Tromsø, Norway; no. 2010/1318-4, no. 2014/365, and no. 2014/1672). All participants provided written informed consent prior to sample collection and analysis. Data were de-identified prior to proteomic and statistical analysis.

### Method details

#### SomaLogic proteomic profiling

Serum proteins were quantified using the SomaScan v4.1 platform (SomaLogic Inc., Boulder, CO), measuring 7326 aptamer-based targets. Data was received as normalized relative fluorescence units (RFU). SomaLogic normalization and hybridization controls were applied by the vendor, followed by hybridization signal calibration and median signal normalization across runs. The affinity proteomics data have been deposited to the PRIDE^90^ repository (PRIDE: PAD000026).

Four samples were identified as outliers using PCA on Mahalanobis distances computed from log_10_-transformed intensities (see DocumentS1: Methods S1A, Figure S1), with statistical inference based on a chi-square test (*p* < 0.1). High-leverage aptamers were identified using Z-scores (±1.8, corresponding to the 2.5th and 97.5th percentiles) on raw intensities and assigned N/A, after which missing values were imputed using the variable’s minimum and maximum post-outlier removal. Further downstream analyses were conducted using log2-transformed raw intensities, as required for linear-model frameworks such as the R packages, *limma* and *variancePartition*.

#### Alignment of aptamer pairs

To assess intra-assay consistency, we compared directional concordance and signal correlation among multiple aptamers targeting the same protein (*n* = 786 proteins, 1,683 aptamers). Directional consensus was defined as ≥ 80% of aptamers showing the same direction of change (ME/CFS vs. HC). Pearson correlations were categorized as weak (|r| < 0.3), moderate (0.3–0.5), strong (0.5–0.8), or very strong (|r| > 0.8). Discordant aptamers were resolved by a predefined decision tree prioritizing significance (*p* < 0.05) and directionality (see DocumentS1: Methods S1B, Table S2).

#### Subcellular and immune annotation

Proteins were annotated according to subcellular localization (intracellular, membrane, secreted, membrane-secreted) and immune-cell association using curated panels from HPA.^44^^,^^91^ Directional changes per class were summarized at the protein level (DocumentS1: Data S1A, Table S3).

#### CycloME responder analysis

Within the CycloME trial subset, proteomic profiles of responders (*n* = 21) and non-responders (*n* = 14) were compared using *limma* with adjustment for age, sex, BMI, and fasting status (SuppData3: 8_CycloME_vs._RvsNR). The PCA, Volcano plot, and top up- and down-regulated proteins are shown in DocumentS1: Data S1B, Figure S2, Tables S4 and S5.

#### Activity and clinical correlates

Associations between protein abundance and physical activity were examined using both SF-36 Physical Functioning (SF-36PF) scores and mean daily step counts derived from accelerometer recordings.^31^^,^^32^ Pearson correlations were computed within aptamer communities (|r| > 0.3, *p* < 0.05).

#### Luminex analysis

Seventy-seven target proteins (Data S6) were quantified using the Luminex Human Discovery Assay (Cat. #LXSAHM; R&D Systems), following the manufacturer’s instructions.

Five custom assays were designed to cover the analytical targets, and two 96-well plates for each assay were used. The majority of the samples were analyzed in single reaction wells; however, some samples were analyzed as duplicates or included on multiple plates for inter-and intra-plate quality control. Each plate included fresh target standard dilutions in duplicates. Plates with identical assays were run on the same day. Measurements were performed using the Luminex 200 instrument (Luminex Corp.) and the accompanying analysis software to generate the standard curves and sample concentrations. Measurements that were out the range of the standard curve were excluded.

#### ELISA

ELISA was performed using Quantikine Ready-to-Use ELISA kits (R&D Systems) for FGF-21 (Cat#: DF2100), C-peptide (Cat#: DICP00), and GDF-15 (Cat#: DG0150). The serum samples were diluted and measured in duplicates, according to the manufacturer’s recommendations. Measurements were performed using the Spark microplate reader (Tecan Trading AG, Switzerland). Standard curve generation was conducted in Excel or GraphPad Prism, with further data analysis done in GraphPad Prism and R programming language.

#### Interstudy comparison

Findings were compared with two published SomaLogic ME/CFS datasets, Germain et al., 2021,^29^ and Walitt et al., 2024.^30^ Both datasets were processed and analyzed using comparable quality-control and *limma* pipelines, although an identical linear regression model was not possible due to missing covariates for the Walitt and Germain datasets. Variable outliers were assessed and handled using identical protocol. Shared up- and down-regulated proteins were identified by gene symbol overlap (DocumentS1: Data S1I; Data S7)).

### Quantification and statistical analysis

For SomaScan data, a principal component analysis (PCA) with the first two principal components used for sample projections, and clinical variables correlated with sample coordinates via the *envfit* function (Vegan v2.7.0). Univariate analysis of SomaScan and Luminex data was performed using linear regression (adjusting for age, sex, BMI, and fasting baseline) with *limma*.^92^ Explained variance was further assessed on variables with statistical significance (*p* < 0.05) using the function *fitExtractVarPartModel* in the *VariancePartition* library (GitHub - DiseaseNeuroGenomics/variancePartition). Semi-partial Pearson correlation coefficients were calculated for relevant subsets, adjusted for the same covariates, were calculated using *ppcor*,^93^ filtered by a standard cutoff (*p* < 0.05, absolute r > 0.3) visualized in a clinical-protein network, where protein communities were defined by shared clinical interactions. Functional enrichment was conducted using Gene Ontology^94^ with *ClusterProfiler* (GitHub - YuLab-SMU/clusterProfiler), while ligand-receptor interactions were annotated via *CellLinker*,^95^ and functional/spatial annotations were retrieved from the Human Protein Atlas.^44^^,^^91^ All visualizations were generated using *ggplot2*, *ComplexHeatmap, tidygraph*, *igraph*, *ggnet*, and *ggpubr*. All statistical analyses for aptamer-based proteomics and Luminex were performed using the R programming language^96^ within the RStudio environment.^97^ BioRender was used for composition of figures. For cross-platform evaluation (SomaScan vs. Luminex), Pearson correlation coefficients were calculated using base R tools, and concordance patterns were compared for shared targets. Significance thresholds were two-sided *p* < 0.05 unless otherwise stated. Comparisons of ELISA data were performed between groups (Welch’s *t* test with FDR correction, q < 0.05), and stratified by sex and metabotype (one-way ANOVA, *p* < 0.05; followed by post-hoc Welch’s *t* test with FDR correction, q < 0.05).

### Additional resources

The clinical trials providing samples for aptamer-based proteomics were RituxME (ClinicalTrials.gov: NCT02229942) and CycloME (ClinicalTrials.gov: NCT02444091).

The supplemental information and Excel files (Data S1, S2, S3, S4, S5, S6, and S7) provide full SomaScan and Luminex datasets, metadata, and statistical outputs.

## Supplemental information

Document S1. Figures S1–S6, Tables S1–S13, and Methods S1

Supplemental data S1. Processed SomaScan dataset with metadata

Supplemental data S2. Multivariate and covariate analyses

Supplemental data S3. Univariate statistics

Supplemental data S4. Tissue-, location-, and immune cell-associated protein panels

Supplemental data S5. Secreted proteins (secretome) and associated functions

Supplemental data S6. Luminex dataset and validation analyses

Supplemental data S7. Comparison with other SomaLogic ME/CFS cohorts

Document S2. Article plus supplemental information
