# Supplementary material for: Charting the circulating proteome in ME/CFS using cross-system profiling to uncover mechanistic insights
Source: Cell Rep Med. 2026 Mar 4;7(3):102647. doi: 10.1016/j.xcrm.2026.102647 (PMC13006441; doi:10.1016/j.xcrm.2026.102647)
Supplement: Document S1. Figures S1–S6, Tables S1–S13, and Methods S1 [file mmc1.pdf]

Cell Reports Medicine, Volume 7

Supplemental information

Charting the circulating proteome in ME/CFS using cross-system profiling to uncover mechanistic insights

August Hoel, Fredrik Hoel, Sissel Elisabeth Dyrstad, Henrique Chapola, Ingrid Gurvin Rekeland, Kristin Risa, Kine Alme, Kari Sørland, Karl Albert Brokstad, Hans-Peter Marti, Olav Mella, Øystein Fluge, and Karl Johan Tronstad

Document S1: Supplementary information

Content:

DocumentS1.pdf (this file)

Methods S1A:	Data and analytical steps	Table S1, Figure S1
Methods S1B:	Alignment between aptamer pairs	Table S2
Data S1A:	Protein-level summary statistics	Table S3
Data S1B:	CycloME responders vs. non-responders	Tables S4- S5, Figure S2
Data S1C:	Correlation with mean number of steps	Table S6, Figure S3
Data S1D:	Immune cell profiles	Table S7
Data S1E:	Blood cell counts	Table S8
Data S1F:	Ligand - receptor alignment	Figure S4
Data S1G:	Luminex cohort	Table S9
Data S1H:	Cross-platform concordance results (SomaScan vs Luminex)	
Data S1I:	Interstudy comparison	Tables S10-S13, Figures S5-S6

Methods S1A: Data and analytical steps

Cohort size:

Table S1: Summary of subjects included in key analytical steps
Related to cohort described in STAR Methods.

Step	ME/CFS	HC
Initial population	54	29
After preprocessing (Outlier removal)	50	29
For univariate analysis using limma (adjusting for BMI, age, sex and fasting)	50	24
Analysis of metabotypes	40	24

Univariate analysis was performed using a linear regression model including BMI, age, sex, and overnight fasting as covariates, as covariate adjustment was required; consequently, five HC subjects lacking these data were omitted (Table S1).

Removal of outliers:

SomaLogic provides normalized data (RFU) and recommends a log10 transformation, as individual outliers can disproportionately influence analyses. The statistical impact of such outliers is well-documented using this platform, notably Germain et. al. 2021 (PMID: 33572894) addressed this as a major challenge for downstream analysis in their aptamer-based ME/CFS study.

Four samples were identified as outliers using PCA on Mahalanobis distances computed from $\log_{10}$-transformed intensities (Figure S1, STAR Methods)

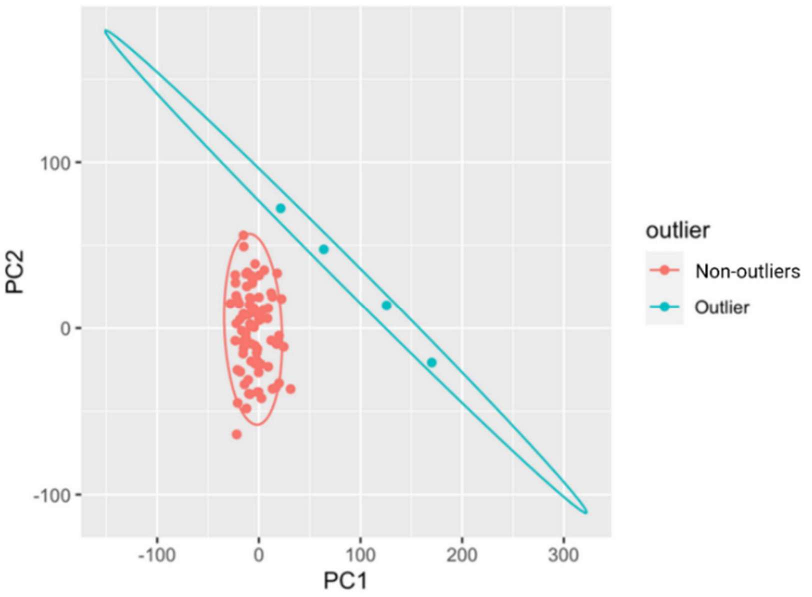

Figure S1. PCA on Mahalanobis distances detecting four outliers.
Related to Somalogic data processing as described in STAR Methods.

Methods S1B: Alignment between aptamer pairs

Aptamer pair alignment analyses

We performed directional concordance and signal consistency analysis between different aptamers targeting the same protein (n = 786 proteins, 1683 aptamers) (SuppData1: 4_Aptamer_pairs_alignment). Overall, moderate to very strong correlation (|R| > 0.3) was shown for 41.6% of the proteins, while 58.4% showed weak correlations (|R| < 0.3) (Table S2). This is most likely explained by isoform- or domain-specific binding effects (e.g., splice variants), since aptamers often recognize different domains of the same protein, and circulating proteins may exist in multiple isoforms, proteolytic fragments, or post-translationally modified forms. Importantly, despite these variations at the molecular level, almost all the significant aptamers reported concordant directional changes (p<0.05) between ME/CFS and controls. As shown by the low numbers in the "Mixed (<80% same dir)" row in Table S2, few proteins reported conflicting effects, supporting the robustness of the group-level findings.

Table S2: Alignment between aptamer pairs. The table shows the number of proteins targeted by multiple aptamers, stratified by directional consensus at group-level (p<0.05 ME/CFS vs HC, rows), across different consistency categories determined by correlation strength (Pearson, |r|) between aptamer-aptamer pairs (columns). Percentages are relative to the total number of proteins targeted by multiple aptamers (786 proteins,1683 aptamers in total). The "Mixed (<80% same dir)" shows the number of proteins targeted by multiple aptamers, where less than 80% of the aptamers reported the same directional change. Related to Figure 1 and SuppData1: 4_Aptamer_pairs_alignment.

Directional Consensus	***Weak (n, %) |R|<0.3***	***Moderate (n, %) |R|=0.3-0.5***	***Strong (n, %) |R|=0.5-0.8***	***Very Strong (n, %) |R|>0.8***	***Total***
(No p<0.05 hits)	*303 (38.8%)*	*54 (6.9%)*	*54 (6.9%)*	*82 (10.5%)*	***493 (62.7%)***
Down (all sig)	*81 (10.4%)*	*17 (2.2%)*	*25 (3.2%)*	*30 (3.8%)*	***153 (19.5%)***
Mixed (<80% same	*7 (0.9%)*	*3 (0.4%)*	–	–	***10 (1.3%)***
Up (all sig)	*68 (8.7%)*	*22 (2.8%)*	*15 (1.9%)*	*25 (3.2%)*	***130 (16.5%)***
Total	***459 (58.4%)***	***96 (12.2%)***	***94 (12.0%)***	***137 (17.4%)***	***786 (100%)***

Method for counting proteins targeted by multiple aptamers

Multiple aptamers can target the same protein; however, these may exhibit discordant statistical significance and opposite directions of change. When counting significant proteins, duplicates are removed. This process, however, depends on the sorted order of the dataset, which may lead to inconsistent results.

To avoid overestimation of significant proteins, duplicated targets were merged using a consistent rule set based on statistical significance and direction of change (logFC):

1. Discordant significance (n = 2):
   If one aptamer had $p < 0.05$ and the other $p \geq 0.05$, the aptamer with $p < 0.05$ was retained.
2. Opposite direction (n = 2):
   If both aptamers had $p < 0.05$ but opposite directions of change, the aptamer with the lowest p value was retained.
3. Multiple aptamers (n > 2) with concordant direction:
   - If the number of significant aptamers (s) < the number of non-significant aptamers (ns), one non-significant aptamer was retained.
   - If $s > ns$, one significant aptamer (the one with the lowest p) was retained.
4. Multiple aptamers (n > 2) with mixed direction:
   The direction (Δ) with the largest number of significant aptamers was retained:
   $s = \max(s_{+1}, s_{-1})$.
5. Equal number of up- and downregulated significant aptamers (n > 2):
   If $s_{+1} = s_{-1}$, the aptamer with the lowest p value was retained, regardless of direction.

Data S1A: Protein-level summary statistics

Table S3: Protein-level changes across subcellular classes. Summary of protein changes within subcellular classes in ME/CFS relative to healthy controls (HC), aggregated at the protein level rather than the aptamer level. The number of proteins and corresponding percentage in each category are shown. "Down" and "Up" indicate lower or higher protein levels in ME/CFS, respectively. To estimate the total number of significant changes, the counts for $p < 0.05$ and $q < 0.05$ can be summed. Related to Figures 1 and 3.

Category	Intracellular	Secreted	Membrane	Membrane & Secreted	All (Excl. NA)	Non-assigned (NA)	All (Incl. NA)
**Down q**	468 (12.7%)	43 (4.1%)	60 (4.2%)	3 (2.1%)	574 (9.1%)	17 (8.7%)	591 (9.1%)
**Down p**	349 (9.5%)	41 (3.9%)	68 (4.8%)	7 (5.0%)	465 (7.4%)	12 (6.1%)	477 (7.3%)
**Total Down**	817 (22.2%)	84 (8.0%)	128 (9.0%)	10 (7.1%)	1,039 (16.5%)	29 (14.8%)	1,068 (16.5%)
**Up q**	92 (2.5%)	65 (6.2%)	53 (3.7%)	5 (3.6%)	215 (3.4%)	5 (2.6%)	220 (3.4%)
**Up p**	190 (5.2%)	99 (9.4%)	124 (8.7%)	8 (5.7%)	421 (6.7%)	14 (7.1%)	435 (6.7%)
**Total Up**	282 (7.7%)	164 (15.5%)	177 (12.4%)	13 (9.3%)	636 (10.1%)	19 (9.7%)	655 (10.1%)
**No Change**	2,573 (70.1%)	808 (76.5%)	1,124 (78.7%)	117 (83.6%)	4,622 (73.4%)	148 (75.5%)	4,770 (73.5%)
**Total excl. NA**	3,672 (100%)	1,056 (100%)	1,429 (100%)	140 (100%)	6,297 (100%)	–	–
**Total incl. NA**	–	–	–	–	–	196 (100%)	6,493 (100%)

Data S1B: CycloME responders vs. non-responders

A) **B)**

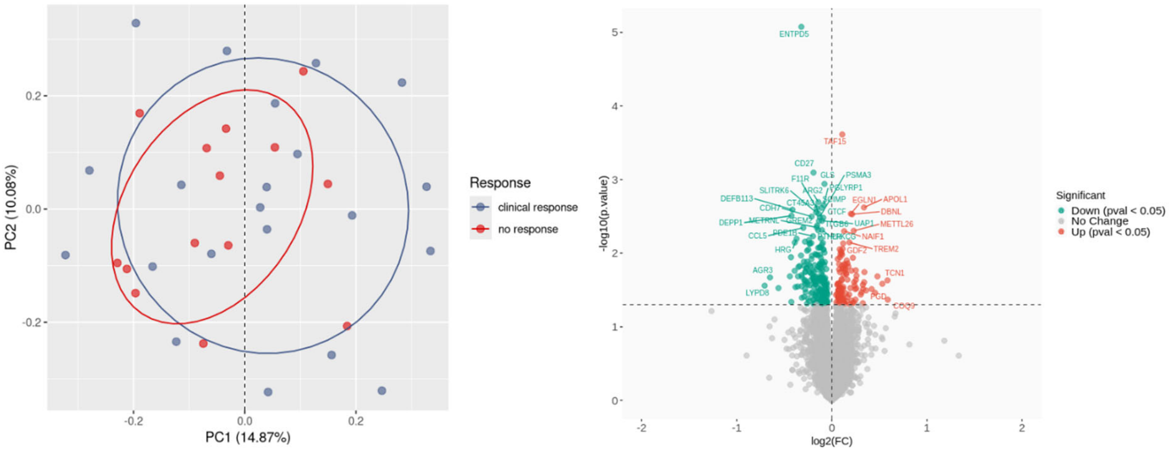

Figure S2. CycloME responders (n=21) and non-responders (n=14). A) PCA, B) Volcano Plot. Related to Figure 1.

Table S4. Univariate Statistics (Limma Regression Model); CycloME responders (n=21) *vs.* non-responders (n=14). Related to Figure 1 and SuppData3: 8_CycloME_vs_RvsNR.

Down(q < 0.05)	Down (p <0.05)	No Change	Up (p < 0.05)	Up (p <0.05)
0	210	7003	113	0

Table S5. Top 25 up- and downregulated proteins, sorted by significance; CycloME responders (n=21) *vs.* non-responders (n=14). Related to Figure 1 SuppData3: 8_CycloME_vs_RvsNR.

Direction	EntrezGene-Symbol	log2FC	P.Value		Direction	EntrezGene-Symbol	log2FC	P.Value
↑Up	TAF15	0.1096	0.00024		↓Down	ENTPD5	-0.3205	0.00001
↑Up	APOL1	0.3381	0.00239		↓Down	CD27	-0.1924	0.00081
↑Up	EGLN1	0.2003	0.00292		↓Down	GLS	-0.0771	0.00115
↑Up	DBNL	0.2139	0.00296		↓Down	ARG2	-0.1409	0.00203
↑Up	METTL26	0.23	0.00496		↓Down	PGLYRP1	-0.0852	0.00221
↑Up	NAIF1	0.1297	0.00502		↓Down	SCIMP	-0.1087	0.00243
↑Up	TREM2	0.1847	0.00715		↓Down	F11R	-0.1583	0.00245
↑Up	GDF2	0.1193	0.00738		↓Down	DEFB113	-0.4139	0.00257
↑Up	ARPC2	0.1135	0.00873		↓Down	SLITRK6	-0.165	0.00279
↑Up	LRTM2	0.0831	0.00887		↓Down	CT45A3	-0.1395	0.00291
↑Up	NDRG4	0.0957	0.00943		↓Down	DEPP1	-0.4238	0.0031
↑Up	LRCH4	0.103	0.00994		↓Down	CDH7	-0.2128	0.00318
↑Up	GK5	0.0908	0.01034		↓Down	PSMA3	-0.1265	0.00324
↑Up	MTHFSD	0.1073	0.01057		↓Down	CTCF	-0.0905	0.00331
↑Up	CDC34	0.085	0.01141		↓Down	UAP1	-0.1028	0.00363
↑Up	MVD	0.2214	0.01157		↓Down	ITGB6	-0.135	0.00365
↑Up	TMEM59L	0.0617	0.012		↓Down	GREM2	-0.1545	0.00422
↑Up	HMGB1	0.1565	0.01254		↓Down	METRNL	-0.1597	0.00447
↑Up	GMPPA	0.1652	0.01383		↓Down	CCL5	-0.3001	0.00453
↑Up	RPS19	0.1305	0.01406		↓Down	PRKCG	-0.1041	0.00486
↑Up	CPD	0.0768	0.01407		↓Down	PTHLH	-0.1436	0.00488
↑Up	SAE1|UBA2	0.144	0.01431		↓Down	PDE1B	-0.1977	0.00588
↑Up	EXTL2	0.1072	0.01486		↓Down	CREBL2	-0.1146	0.00634
↑Up	IGF2R	0.1733	0.01513		↓Down	HRG	-0.3707	0.00638
↑Up	LGALS1	0.1223	0.01596		↓Down	MATN4	-0.2411	0.00667

Data S1C: Correlation with mean number of steps

In our main analysis, we used SF-36 Physical Functioning (SF-36PF) as a proxy for physical activity, reasoning that lower SF-36PF scores would likely reflect reduced activity and thus potential deconditioning. The broad proteomic changes observed in ME/CFS that were unrelated to SF-36PF therefore suggested that these changes were not driven by reduced activity or deconditioning.

To further address this point, we performed an additional analysis using mean daily step count (per 24h), which provides a more direct measure of physical activity. We found that a large fraction of aptamers correlating with SF-36PF also correlated with mean step count, confirming that SF-36PF is a good surrogate for activity level. Importantly, the main proteomic changes distinguishing ME/CFS from controls remained unrelated to either SF-36PF or step count, supporting that these changes are not primarily driven by deconditioning.

We acknowledge that reduced activity can have negative physiological effects; however, our findings suggest that these effects are largely separate from the disease-specific serum proteome alterations observed in ME/CFS.

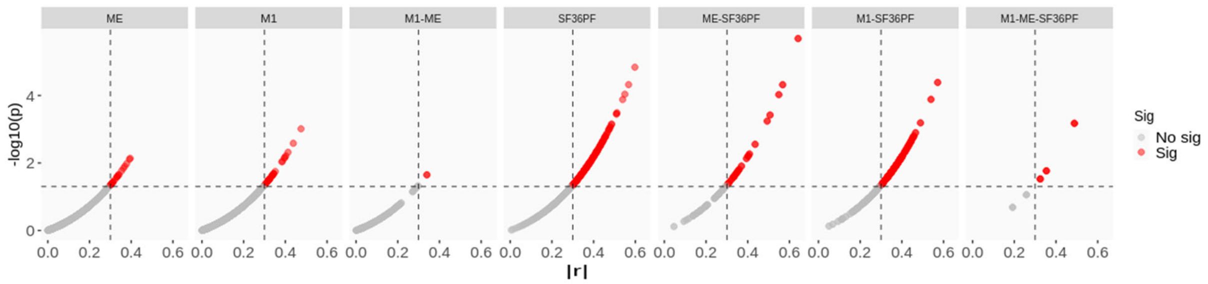

Figure S3. Correlation with mean steps. The figure displays how aptamers correlated to mean steps within each aptamer community. Related to Figure 2 and SuppData2: 5_Correlation_steps.

Table S6. Aptamers correlated to Mean Steps. Threshold for correlation |r| > 3 & p < 0.05. Related to Figure 2 and SuppData2: 5_Correlation_steps.

Community	Correlated	Not Correlated	% Correlated
ME	19	756	2.5%
ME-SF36PF	46	72	39.0%
M1	30	684	4.2%
M1-ME	2	168	1.2%
M1-ME-SF36PF	9	6	60.0%
M1-SF36PF	238	184	56.4%
SF36PF	191	260	42.3%

Data S1D: Immune cell profiles

Table S7. Immune cell profiles. Summary of protein changes within immune cell-associated panels in ME/CFS relative to healthy controls (HC). For each cell type, the number and corresponding percentage of aptamers targeting proteins in each category are shown. "Down" and "Up" indicate lower or higher protein levels in ME/CFS, respectively. To estimate the total number of significant changes, the counts for $p < 0.05$ and $q < 0.05$ can be summed. Related to Figure 4D and SuppData4, 4_ImmuneCells.

**Cell Type**	Down (qval < 0.05)	Down (pval < 0.05)	No Change	Up (pval < 0.05)	Up (qval < 0.05)
**B-cells**	8 (4.3%)	8 (4.3%)	148 (79.6%)	17 (9.1%)	5 (2.7%)
**Dendritic**	20 (4.7%)	22 (5.2%)	337 (79.5%)	24 (5.7%)	21 (5.0%)
**Granulocytes**	51 (8.6%)	46 (7.7%)	458 (76.8%)	27 (4.5%)	14 (2.3%)
**Monocytes**	34 (7.5%)	30 (6.7%)	352 (78.0%)	22 (4.9%)	13 (2.9%)
**NK-cells**	0 (0.0%)	7 (5.7%)	97 (79.5%)	14 (11.5%)	4 (3.3%)
**T-cells**	14 (3.6%)	16 (4.1%)	310 (79.5%)	36 (9.2%)	14 (3.6%)

Data S1E: Blood cell counts

Table S8. Blood cell counts for the ME/CFS group. Summary of blood cell counts in ME/CFS patients participating in the RituxME and CycloME trials. Blood cell data were not available for healthy controls; however, comparison with normal ranges indicated no abnormalities in blood cell counts within the ME/CFS group. Related to Figure 4D and Table S7.

**Measurement**	**Mean**	**SD**	**n**	**Normal**	**Unit**
**Hemoglobin**	14.26	1.16	41	*11.7 - 15.3*	g/dL
**MCV**	88.54	2.78	41	*82 - 98*	fL
**Leucocytes**	6.23	1.35	41	*4 - 10*	10^9/L
**Neutrophile granulocytes**	3.38	1.03	41	*1.5 - 7.3*	10^9/L
**Lymphocytes**	2.20	0.80	41	*1.1 - 3.3*	10^9/L
**Monocytes**	0.45	0.12	41	*0.2 - 0.8*	10^9/L
**Eosinophiles**	0.16	0.11	41	*< 0.4*	10^9/L
**Basophils**	0.05	0.05	41	*< 0.2*	10^9/L
**Thrombocytes**	273.49	64.39	41	*145 - 350*	10^9/L

Data S1F: Ligand – receptor alignment

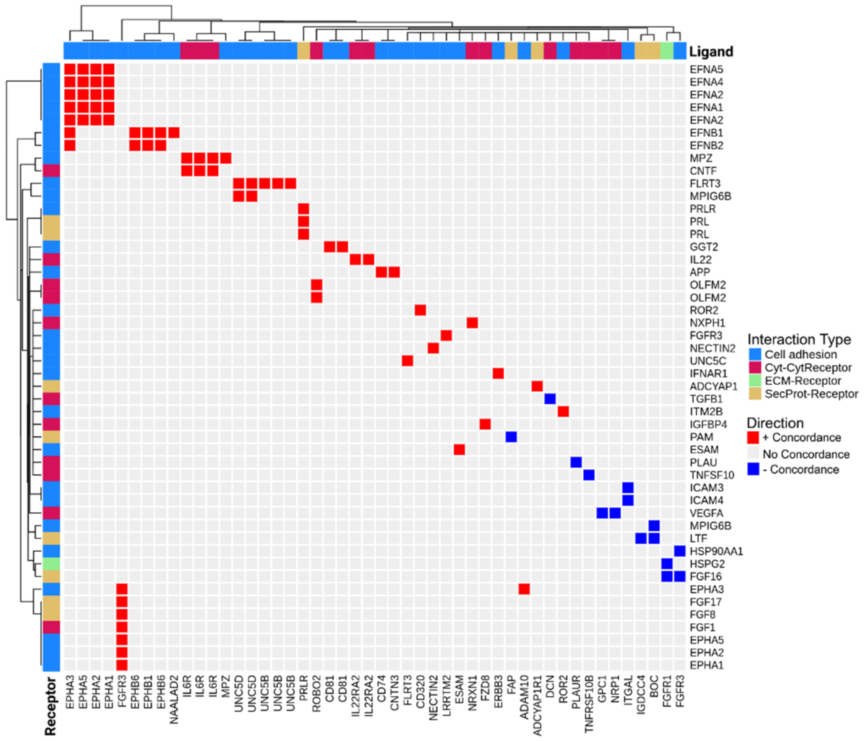

Figure S4: Ligand – receptor alignment. We performed a ligand - receptor alignment analysis to identify pairs of ligands and receptors whose levels change in a coordinated manner. Related to Figure 5.

Data S1G: Luminex cohort

Table S9: Luminex cohort characteristics. The table summarizes group sizes, the percentage (%) of original SomaLogic samples included, and key group characteristics. Related to Figure 6

**Group**	**N (% ovelap *vs.* Somalogic)**	**Age (mean ± SD)**	**Sex (F/M)**	**Female %**	**BMI (kg/m², mean ± SD)**	**Fasting n (%)**
**ME/CFS**	83 (48.2%)	35.0 ± 11.3	64 / 19	77.1%	24.4 ± 4.5	12 (14.5%)
**HC**	29 (20.7%)	36.2 ± 9.6	18 / 11	62.1%	24.0 ± 2.3	0 (0.0%)
**Metabotype 1**	32 (40.62%)	35.2 ± 12.5	24 / 8	75.0%	23.1 ± 4.0	5 (15.6%)
**Metabotype 2**	38 (52.6%)	33.8 ± 11.4	29 / 9	76.3%	25.7 ± 5.0	6 (15.8%)
**Metabotype 3**	13 (53.8%)	37.9 ± 7.8	11 / 2	84.6%	23.8 ± 2.8	1 (7.7%)
**Total**	112	35.3 ± 10.9	82 / 30	73.2%	24.3 ± 4.0	12 10.7%)

Data S1H: Cross-platform concordance results (SomaScan vs Luminex)

Extended description of findings: The examination of directional concordance compared the 54 Luminex proteins with the 68 corresponding aptamers on the SomaScan platform (Fig 6A; SuppData6: 5_Directional_consensus). Here is a summary of the data:

- 38 proteins (70.4 %) / 44 aptamers (64.7 %) showed concordant results (either no significant change or significant change in the same direction).
- 4 proteins (7.4 %) / 10 aptamers (13.5 %) had mixed results because different aptamers targeting the same protein gave differing changes (notably BDNF, COL1A1, OSM, Osteoactivin).
- 12 proteins (22.2 %) / 14 aptamers (18.9 %) were discordant (opposite direction of change between platforms)

Most discordant proteins had low logFC2, except Myoglobin and NAGLU, which showed larger effects on SomaScan than on Luminex.

Signal consistency analysis (Fig. 6B; SuppData6: 6_Signal_correlation), 48 of 68 comparisons (69.1%) showed significant positive correlations ($p < 0.05$), and none showed negative correlations, supporting the robustness of the analysis.

Data S1I: Interstudy comparison

ME/CFS datasets using the aptamer-based platform (SomaLogic):

- Hoel 2025: this study
- Germain 2021: PMID 33572894
- Walitt 2024: PMID 38383456

The compared data are provided in SuppData7.

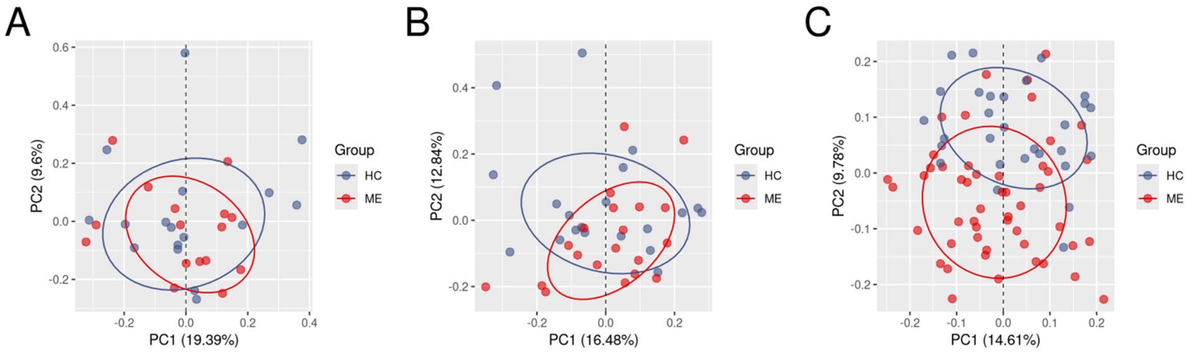

Figure S5. Principal Component Analysis of each Dataset. A) Walitt: PMID 33572894, B) Germain: PMID 38383456, C) Hoel: This study. Related to the Discussion and SuppData7.

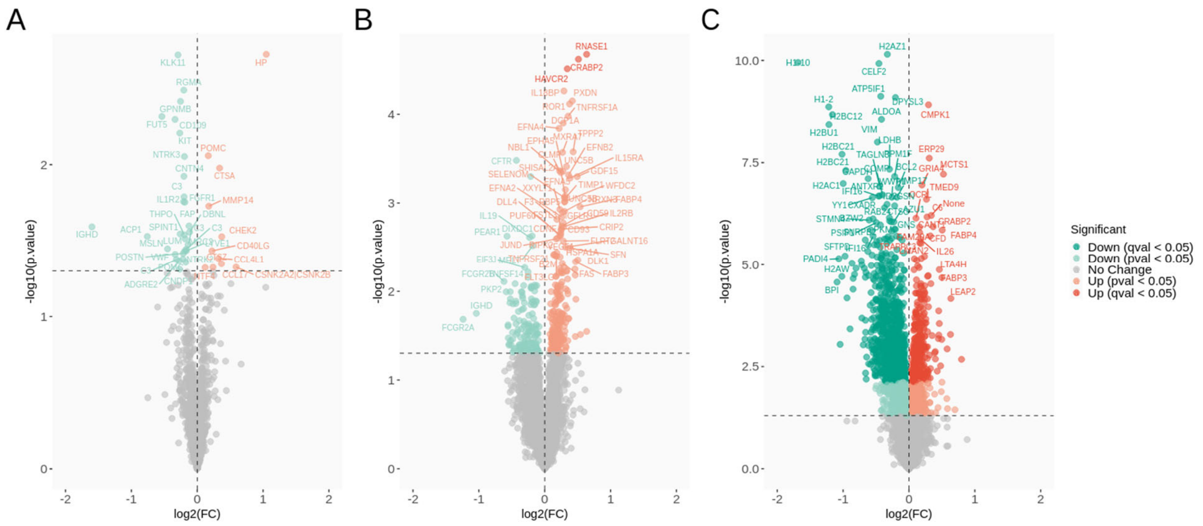

Figure S6. Volcano Plots. A) Walitt: PMID 33572894, B) Germain: PMID 38383456, C) Hoel: This study. Related to the Discussion and SuppData7.

Table S10. Shared Changes. Related to the Discussion and SuppData7.

**Overlaps**	**Up**	**Down**
Germain *vs.* Hoel	*67*	*55*
Germain *vs.* Walitt	*1*	*3*
Hoel *vs.* Walitt	*2*	*5*

Table S11. Changes across compartment | Walitt. Related to the Discussion and SuppData7.

**Compartment**	**Down**	**No change**	**Up**
Intracellular	3 (0.8%)	385 (98.7%)	2 (0.5%)
Membrane	11 (3.7%)	287 (95.7%)	2 (0.7%)
Membrane and secreted isoforms	2 (3.0%)	64 (95.5%)	1 (1.5%)
Secreted	13 (2.9%)	423 (95.9%)	5 (1.1%)
Unannotated	1 (1.4%)	69 (97.2%)	1 (1.4%)

Table S12. Changes across compartments | Germain. Related to the Discussion and SuppData7.

**Compartment**	**Down**	**No change**	**Up**
Intracellular	71 (3.7%)	1757 (92.8%)	66 (3.5%)
Membrane	36 (3.1%)	1062 (90.8%)	71 (6.1%)
Membrane and secreted isoforms	5 (4.0%)	111 (88.1%)	10 (7.9%)
Secreted	15 (1.6%)	854 (91.2%)	67 (7.2%)
Unannotated	13 (9.4%)	120 (87.0%)	5 (3.6%)

Table S13. Changes across compartments | Hoel. Related to the Discussion and SuppData7.

**Compartment**	**Down**	**No change**	**Up**
Intracellular	910 (23.3%)	2669 (68.2%)	332 (8.5%)
Membrane	160 (9.3%)	1339 (78.0%)	217 (12.6%)
Membrane and secreted isoforms	13 (7.1%)	152 (82.6%)	19 (10.3%)
Secreted	104 (8.0%)	986 (76.3%)	202 (15.6%)
Unannotated	35 (15.7%)	165 (74.0%)	23 (10.3%)
