# Supplementary material for: Charting the circulating proteome in ME/CFS using cross-system profiling to uncover mechanistic insights
Source: Cell Rep Med. 2026 Mar 4;7(3):102647. doi: 10.1016/j.xcrm.2026.102647 (PMC13006441; doi:10.1016/j.xcrm.2026.102647)
Supplement: Document S2. Article plus supplemental information [file mmc9.pdf]

Cell Reports
Medicine

Article

Charting the circulating proteome in ME/CFS using cross-system profiling to uncover mechanistic insights

Graphical abstract

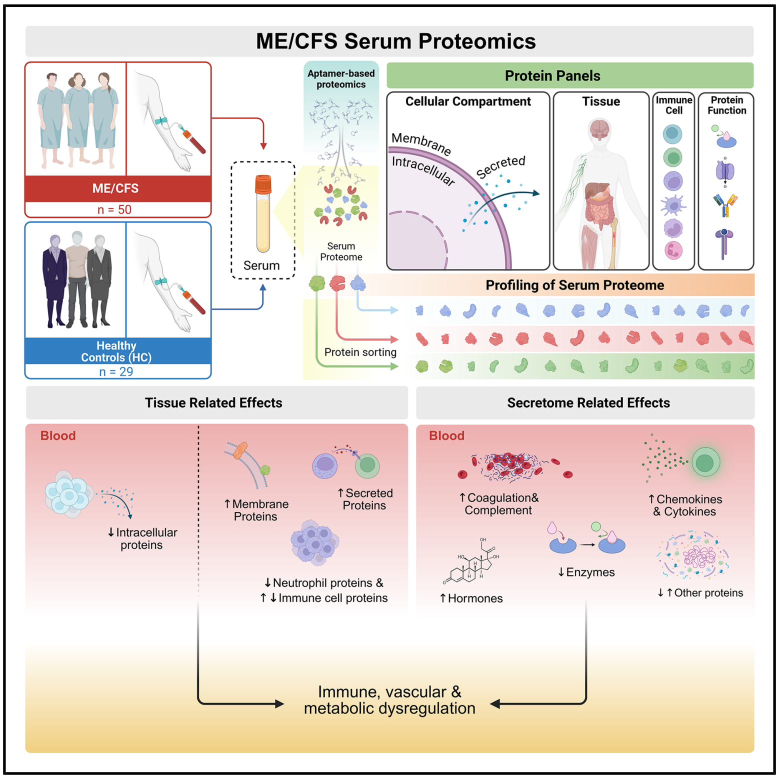

Highlights

- Serum proteomics reveals widespread protein changes in ME/CFS patients
- Tissue-linked shifts show reduced intracellular and increased secreted proteins
- Immune signatures show reprogramming with reduced neutrophil-derived proteins
- Regulatory networks link immune, vascular, and metabolic dysfunction

Authors

August Hoel, Fredrik Hoel,
Sissel Elisabeth Dyrstad, ..., Olav Mella,
Øystein Fluge, Karl Johan Tronstad

Correspondence

karl.tronstad@uib.no

In brief

Hoel et al. characterize circulating proteomic signatures in ME/CFS and identify widespread tissue-linked alterations, including increased secreted proteins and reduced intracellular proteins. Protein changes affecting immune, metabolic, and vascular processes support immune reprogramming and define regulatory networks that provide pathobiological insight relevant to biomarker development and future therapeutic strategies.

Hoel et al., 2026, Cell Reports Medicine 7, 102647
March 17, 2026 © 2026 The Author(s). Published by Elsevier Inc.
https://doi.org/10.1016/j.xcrm.2026.102647

CellPress

Cell Reports Medicine

CellPress
OPEN ACCESS

Article

Charting the circulating proteome in ME/CFS using cross-system profiling to uncover mechanistic insights

August Hoel,[1,2,7] Fredrik Hoel,[1,7] Sissel Elisabeth Dyrstad,[1] Henrique Chapola,[1] Ingrid Gurvin Rekeland,[3] Kristin Risa,[3] Kine Alme,[3] Kari Sørland,[3] Karl Albert Brokstad,[4] Hans-Peter Marti,[2,5] Olav Mella,[3,6] Øystein Fluge,[3,6] and Karl Johan Tronstad[1,8,*]
[1]Department of Biomedicine, University of Bergen, Bergen, Norway
[2]Department of Clinical Medicine, University of Bergen, Bergen, Norway
[3]Cancer Clinic, Haukeland University Hospital, Bergen, Norway
[4]Department of Safety, Chemistry and Biomedical Laboratory Sciences, Western Norway University of Applied Sciences, Bergen, Norway
[5]Department of Medicine, Haukeland University Hospital, Bergen, Norway
[6]Department of Clinical Science, University of Bergen, Bergen, Norway
[7]These authors contributed equally
[8]Lead contact
*Correspondence: karl.tronstad@uib.no
https://doi.org/10.1016/j.xcrm.2026.102647

SUMMARY

Myalgic encephalomyelitis/chronic fatigue syndrome (ME/CFS) is a debilitating condition often triggered by infections, with unclear mechanisms and no established biomarkers or treatments. We apply aptamer-based serum proteomics to 50 ME/CFS patients and 29 healthy controls, analyzing 7,326 protein targets. We identify 1,823 aptamers with significant differences between the groups (845 after false discovery rate [FDR] correction). Distinct patterns of tissue- and process-specific changes are seen. There is a broad increase in secreted proteins, while intracellular proteins, e.g., from skeletal muscle, particularly show reduction. Immune cell-associated signatures indicate immune reprogramming, including a distinct reduction in proteins secreted by activated neutrophils. Focused secretome analysis supports intensified regulatory interactions related to immune activity, inflammation, vasculature, and metabolism. Validation of measurements using antibody-based methods confirms findings for a selection of proteins. The uncovered serum proteome patterns in ME/CFS patients may contribute to understanding the pathophysiology and inform future biomarker research and therapeutic development.

INTRODUCTION

Myalgic encephalomyelitis/chronic fatigue syndrome (ME/CFS) is a debilitating disease that often begins abruptly following an infection and is increasingly recognized as part of the broader category of post-acute infection syndromes.[1–5] Using the Canadian consensus criteria,[6] the pre-COVID-19 pandemic prevalence of ME/CFS was estimated to be between 0.2% and 0.8%.[7–9] Key symptoms include profound fatigue, post-exertional malaise (PEM), sensory hypersensitivity, pain, unrefreshing sleep, and cognitive dysfunction.[10,11] The consequences of ME/CFS are severe for both patients and their families, with high socio-economic costs.[9,12–14] There is an urgent need for more knowledge about the biological mechanisms of ME/CFS to develop effective diagnostic markers and treatments.

Although the etiology of ME/CFS remains unclear, possible roles of immune system dysregulation, chronic inflammation, and impaired metabolism, have been suggested based on research findings.[1,15] We are currently investigating the hypothesis that ME/CFS may involve an autoimmune mechanism leading to vascular dysregulation, causing tissue hypoperfusion and hypoxia, especially upon exertion, resulting in both short- and long-term effects on energy metabolism.[16,17] This mechanism could explain key aspects of symptom generation and fatiguability in ME/CFS, potentially involving exertion-triggered muscle abnormalities, as recently reported in association with PEM in long COVID patients.[18]

Human cells express thousands of proteins in tissue-specific patterns,[19,20] many of which enter the bloodstream either secreted as functional messengers or through tissue leakage.[21] Disease processes alter both protein expression and release, shaping the circulating proteome and offering insights into tissue homeostasis, secretory regulation, and metabolism.[20] Previous ME/CFS studies have reported variable abnormalities in cytokines and immune factors,[22,23] vascular regulators,[24] and metabolic or muscle-derived messengers,[17,25] highlighting the need for deeper proteomic investigation.

Aptamer microarray proteomics enables high-throughput measurement of thousands of circulating proteins from small sample volumes using protein-specific DNA/RNA aptamers.[26]

© 2026 The Author(s). Published by Elsevier Inc.
This is an open access article under the CC BY license (http://creativecommons.org/licenses/by/4.0/).

Table 1. Group characteristics

Study groups	HC	ME	*p* value
Subjects, *n* (%)	29 (100)	50 (100)	–
Women, *n* (%)	21 (72)	40 (80)	–
Men, *n* (%)	8 (28)	10 (20)	–
RituxME, *n*	N/A	15	–
CycloME, response, *n*	N/A	21	–
CycloME, no response, *n*	N/A	14	–
Fasting, *n* (%)	0 (0)	11 (22)	–
BMI (mean ± SD)	24.0 ± 2.6[b]	24.4 ± 4.2	0.6495
Age	38.4 ± 9.3	40.4 ± 10.1	0.3615
Infection, *n* (%)	N/A	38 (76)	–
Mean steps 24 h (mean ± SD)	N/A	3,021 ± 2,001	–
SF-36PF (mean ± SD)	N/A	31.2 ± 19.3	–
Self-reported PF (%, mean ± SD)	N/A	16.6 ± 7.7	–
Glucose (mM, mean ± SD)	5.04 ± 0.40	5.24 ± 0.82	0.3828
TAG (mM, mean ± SD)	0.87 ± 0.31	1.35 ± 0.89	0.0254[a]
NEFA (mM, mean ± SD)	0.21 ± 0.13	0.40 ± 0.28	0.0262[a]

Overview of the two groups of HC subjects and ME/CFS patients. The ME/CFS samples were obtained from participants in the RituxME and CycloME trials. Measurements of glucose, TAG, and NEFA were performed in the clinic.
N/A, not applicable; NEFA, non-esterified fatty acid; self-reported PF, self-reported physical function; SF-36PF, SF-36 physical functioning; TAG, triacylglycerol.
[a]$p < 0.05$, Welch's test.
[b]HC BMI $n = 24$.

This platform has been increasingly applied to studies of human disease, including aging, kidney disease, and diabetes,[27,28] as well as in two previous ME/CFS studies involving relatively small cohorts.[29,30] Here, we used this approach to map serum protein patterns in ME/CFS, aiming to reveal coordinated cellular, tissue-level, and systemic processes, with particular attention to immune-related mechanisms that may underlie vascular and metabolic dysfunction.

RESULTS

Characterization of the subjects

The curated dataset included 50 serum samples from ME/CFS patients collected at baseline in two clinical intervention trials (RituxME, NCT02229942, 2014–2017,[31] and CycloME, NCT02444091, 2015–2020[32]), along with 29 sex- and age-matched healthy controls (HCs) (STAR Methods; Table 1; Figure 1A). Eleven patients had fasted overnight prior to sampling. Blood glucose levels did not differ between groups (Table 1), but triacylglycerols and non-esterified fatty acids were higher in ME/CFS. This is consistent with our earlier metabolomics study of samples from the same biobank, which identified three metabolic phenotypes (metabotypes): M1 (more lipolytic/ketogenic), M2 (more lipid accumulation), and M3 (metabolically similar to controls).[17] These metabotypes showed only minor influence from sex, BMI, age, medication, or fasting, but possible associations with physical impairment and disease severity. In the present study, we applied these metabotypes to explore links between serum proteomic and metabolomic alterations in ME/CFS.

Comprehensive differences between the ME/CFS group and the HC group

The dataset comprised 7,326 aptamers that collectively recognized 6,493 protein *targets* listed by "TargetFullName," with some aptamers binding to the same target (Supplemental Data S1). These corresponded to 6,408 *proteins* identified by their "EntrezGeneSymbol," because several targets represented different isoforms, fragments, or other proteoforms of the same gene product (Supplemental Data S1). For the 786 proteins recognized by two or more aptamers, we examined both signal consistency (Pearson correlation) and group-level directional concordance (Supplemental Data S1; Document S1: Methods S1B, Table S2). Overall, 41.6% of proteins showed moderate to strong correlations ($|R| > 0.3$), while 58.4% showed weak or none ($|R| < 0.3$), consistent with proteoform- or domain-specific binding effects. Despite these molecular variations, 97.8% of the aptamer pairs showing statistical significance ($p < 0.05$) displayed directional concordance for the differences between ME/CFS and HC groups, supporting the robustness of the group-level findings.

Although the PCA (principal-component [PC] analysis) plot showed overlap between the ME/CFS and HC groups, PERMANOVA confirmed a global group-level separation ($F = 14.0$, $R^2 = 0.15$, $p = 0.001$), which was primarily due to differences along the PC2 dimension (*y* axis, 9.6% explained variance) (Figures 1B and 1C; Supplemental Data S2). Thus, the overlap along the PC1 dimension (*x* axis; 14.5% explained variance) primarily reflected intragroup protein variation shared between ME/CFS and control samples. This makes sense, as PC1 was more influenced by the covariate factors, age, sex, BMI, and fasting state, as indicated by the respective loadings, compared to PC2 (Figure 1C; variable, PC1, PC2, *p* of the correlation; BMI, 0.943, −0.333, 0.106; age, −0.981, −0.195, 0.381; sex, −0.925, −0.380, 0.091; fasting, −0.830, −0.557, 0.046). Therefore, we concluded that group separation along PC2 was not primarily driven by the covariates age, sex, BMI, and fasting state, but rather may reflect disease-specific changes. Accordingly, the major PC2-driving proteins (i.e., with high loadings) showed more statistically significant differences between the two groups, compared with major PC1-driving proteins. Additional analyses aimed at distinguishing disease-specific effects from covariate influences were included in the subsequent investigations.

A total of 1,823 aptamers differed significantly between ME/CFS and HC groups ($p < 0.05$), with 845 remaining significant after false discovery rate (FDR) correction ($q < 0.05$) (Supplemental Data S3). After merging duplicated targets according to a consistent rule set (Document S1: Methods S1B), 1,723 unique proteins were identified as altered, including 811 significant after FDR correction (Document S1: Data S1A, Table S3). More than 60% of the altered aptamers showed lower abundance of their protein targets in ME/CFS (61.8% at $p < 0.05$;

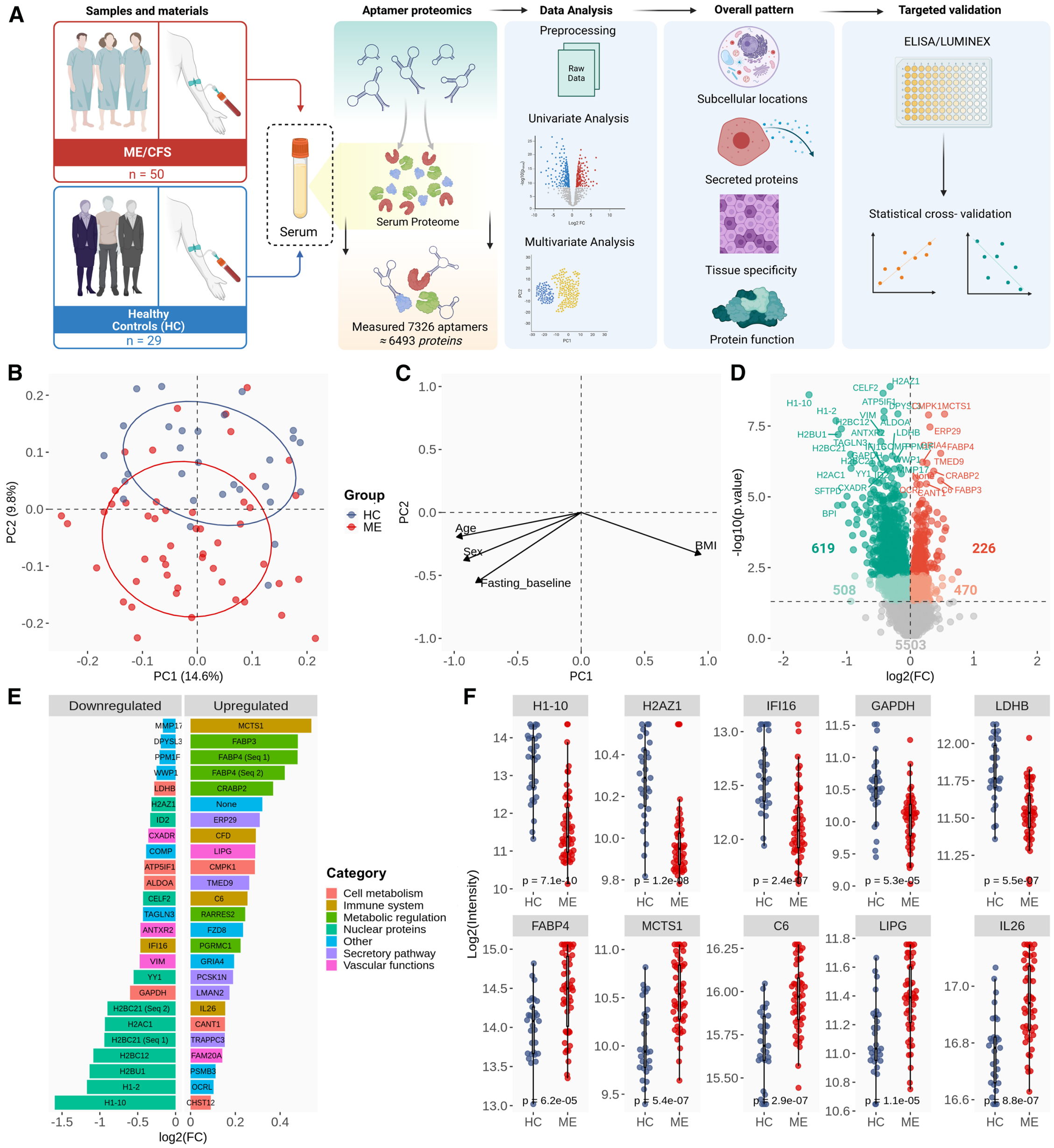

Figure 1. Serum proteomics comparing ME/CFS (ME) patients with HC

(A) Study approach: Serum protein concentrations for 50 ME/CFS patients and 29 HCs were measured using aptamer-based technology (SomaScan v.4.1 7 k). Cross-system profiling was conducted to characterize cellular and tissue-specific impacts, effects on the secretome, and the associated molecular and biological functions involved. Antibody-based measurements were used to validate and expand on findings, with a focus on the immune system and energy metabolism.

(B and C) (B) PCA with overlay of the ME/CFS and HC groups; (C) projections of associated eigenvalues reflecting the influence of age, sex, BMI, and fasting on the PC1 and PC2 dimensions.

(D) Volcano plot displaying all the *p* and q significant features. The dotted line indicates −log10(0.05), i.e., the *p* value significance threshold.

(E) Aptamers were grouped by regulation direction (logFC >0 or <0). The 25 most significant aptamers per group (ranked by *p* value) are shown, ordered by log_2(FC) and color coded by the established biological roles of their protein targets. Seq1 and Seq2 refer to two different aptamers targeting the same protein (FABP4 in this case). “None” means that the protein has not yet been assigned with a gene symbol in the NCBI Entrez Gene database.

(F) Group comparison of selected highly affected serum proteins (median ±1.5×IQR [interquartile range]). Nominal *p* values are displayed for each comparison (Welch’s *t* test). The statistical outcomes of the study included both uncorrected (*p* value) and multiple comparison-corrected (FDR, q value) univariate analyses, adjusted for sex, age, BMI, and overnight fasting.

73.3% at q < 0.05), while the remainder exhibited higher levels (38.2% using $p < 0.05$; 26.7% at q < 0.05) (Figure 1D). To avoid the risk of missing biologically meaningful patterns, given the small effect sizes and intragroup heterogeneity, *p* values were used for exploratory hypothesis-generating investigations, whereas q values were used to indicate robust findings.

Initial key observations related to the possible pathomechanism

The 25 most altered proteins in the ME/CFS group revealed some patterns that were further supported by the subsequent analyses: intracellular proteins, including histones and metabolic enzymes, were predominantly reduced, whereas secretory proteins involved in immune regulation, systemic metabolism, and vascular function were frequently elevated (Figure 1E). Individual examples of highly affected proteins are shown in Figure 1F to illustrate both the magnitude of heterogeneity and the degree of overlap between groups.

Among the proteins showing the lowest levels in ME/CFS were nuclear histones (H1x, H2.1, and several H2B and H2A types) (Figures 1E and 1F), which can be released by activated neutrophils, e.g., as part of neutrophil extracellular traps.[33] Other proteins showing pronounced lower levels included enzymes related to cellular energy metabolism, such as glyceraldehyde-3-phosphate dehydrogenase (GAPDH), fructose-bisphosphate aldolase A (ALDOA), and L-lactate dehydrogenase B chain (LDHB), and proteins linked to vascular function and hypoxia responses, including vimentin (VIM),[34] anthrax toxin receptor 2 ([ANTXR2], also known as capillary morphogenesis gene 2),[35] coxsackievirus and adenovirus receptor (CXADR),[36] and ATP synthase inhibitory factor subunit 1 (ATP5IF1).[37] Additional decreases were observed for the neuronal protein transgelin-3 (TAGLN3) and cartilage oligomeric matrix protein (COMP).

Conversely, several immune-related proteins were among the most highly elevated, including malignant T-cell-amplified sequence 1 (MCTS1),[38,39] complement factor D (CFD), complement component C6 (C6), and the cytokine interleukin (IL)-26 (Figures 1E and 1F). There were also increases in metabolic stress-associated proteins, such as fatty acid-binding proteins 3 and 4 (FABP3 and FABP4).[40] Furthermore, increases were seen for cellular retinoic acid-binding protein 2 (CRABP2), a member of the fatty acid-binding protein family; the adipokine and immune cell chemoattractant retinoic acid responder protein 2 ([RARRES2]; chemerin)[41]; and endothelial cell-derived lipase (LIPG), which is involved in lipoprotein metabolism and vascular biology[42] as well as immune activation.[43] There were also higher levels of endoplasmic reticulum resident protein 29 (ERP29); transmembrane emp24 domain-containing protein 9 (TMED9); pseudokinase FAM20A, which may function in hematopoiesis; and soluble calcium-activated nucleotidase 1 (CANT1).

Univariate logistic regression analyses highlighted aptamers such as ALDOA, DPYSL3, H1-10, and ATP5IF1 with areas under the curve around 0.87 and balanced sensitivity (~0.78–0.88) and specificity (~0.79–0.90) (Supplemental Data S3), although these remain exploratory without external validation. Although the proteomic profiles of the 21 cyclophosphamide responders versus 14 non-responders overlapped by PCA (Supplemental Data S3; Document S1: Data S1B, Figure S2; Tables S4 and S5), several immune-related proteins (e.g., IGHE, KIT, CD80, and IL-34) showed nominally significant differences ($p < 0.05$), none of which remained significant after FDR correction.

Influence by covariates, metabotype, and physical function/activity

We assessed the influence of sex, age, BMI, and fasting on protein levels by comparing the variance of each covariate explained relative to ME/CFS diagnosis (Supplemental Data S2). Figure 2A displays the 30 aptamers showing the highest variance for each covariate. Overall, the aptamers highly influenced by ME/CFS showed relatively small additional influence from the tested covariates, suggesting that these changes largely reflect disease-specific alterations. Several of the aforementioned affected proteins showed such specificity to ME/CFS, such as histones (e.g., H2AZ1 and H1-10), VIM, MCTS1, and LDHB. By contrast, sex, age, BMI, and fasting associated with other, partially overlapping, sets of proteins, e.g., metabolic hormones such as leptin, FABP3, and FABP4, which showed mixed influence patterns.

To further assess potential associations with the metabolic context and physical function, we correlated ME/CFS diagnosis, SF-36 Physical Function (SF-36PF), and metabotype (using metabotype M1 as reference[17]), adjusting for sex, age, BMI, and fasting (Figures 2B–2D; Supplemental Data S2). Of 924 aptamers associated with ME/CFS, 775 were unique to the diagnosis and enriched for intracellular transport/translation. Metabotype and SF-36PF influenced 1,015 and 726 aptamers, respectively, with distinct enrichments: metabotype-linked proteins involved hormone secretion and smooth-muscle cell regulation, while SF-36PF-linked proteins related to tissue homeostasis and nervous system development. A subset of 149 aptamers overlapped with both ME/CFS and either metabotype or SF-36PF, highlighting shared immune-metabolic and extracellular matrix pathways, which may link disease status to metabolic context and clinical severity. Only five proteins showed joint influence by ME/CFS status, SF-36PF, and metabotype (IL-22, HTRA1, OXCT1, ENPP5, and FAS).

Furthermore, a large fraction of aptamers within the SF-36PF cluster also correlated with mean 24-h steps count, a direct measure of physical activity (Supplemental Data S2; Document S1: Data S1C, Figure S3; Table S6). In contrast, very few aptamers within the cluster distinguishing ME/CFS from controls showed such correlations, suggesting that these proteomic patterns are not primarily driven by the lack of activity due to impaired physical function.

Overall, these results indicate that a substantial proportion of the broad proteomic alterations in ME/CFS cannot be explained by demographic factors or deconditioning, but instead may point to intrinsic disease-associated molecular changes.

Cellular proteins in circulation: Release patterns

Cellular proteins enter the bloodstream through both active secretion and passive release from cell turnover or damage, making biological context essential for interpreting altered protein levels. We applied resources such as the Human

CellPress
OPEN ACCESS

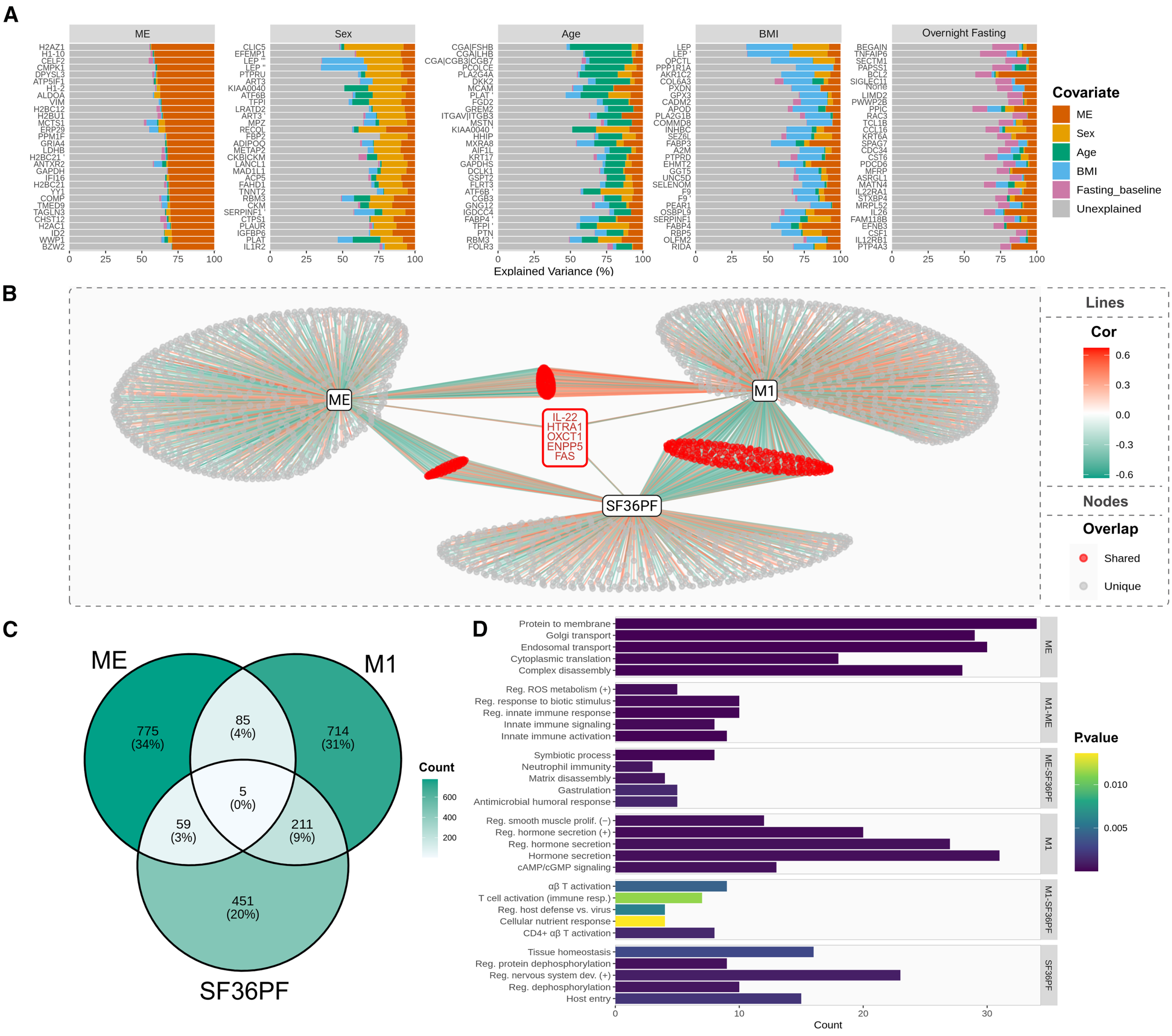

Figure 2. Analysis of influence by covariate factors

(A) The proportion of variance explained by each covariate (ME, sex, age, BMI, and fasting state), across the top 30 most explained features per covariate (different colors).

(B) Semi-partial Pearson correlation network displaying associations between aptamers and ME/CFS diagnosis (ME), metabotype (represented by metabotype M1), and SF-36PF, adjusted for sex, BMI, overnight fasting, and age. Color of lines (Cor) indicates direction (red, positive; green, negative), and intensity shows the strength of the correlation.

(C) Venn diagram summarizing the data in (B).

(D) Gene Ontology enrichment analysis of biological processes within network-derived clusters. The *x* axis indicates aptamer counts within category, and bare color shows significance level (*p* value).

Protein Atlas (HPA) and a curated human secretome database[44] to contextualize the circulating proteome patterns in terms of tissue and cell origin, secretion status, and biological function.

Based on subcellular compartment annotations for all 7,326 aptamers, 53.4% (n = 3,911) targeted intracellular proteins, 25.9% (n = 1,900) membrane-associated proteins (membrane + membrane & secreted), and 17.6% (n = 1,292) secreted proteins, while 3.0% (n = 223) were unannotated (Figure 3A, Supplemental Data S3). Among the 1,823 aptamers showing significant group differences ($p < 0.05$), 1,146 targeted intracellular proteins (Figure 3B), of which 74.9% showed lower serum concentration in ME/CFS, indicating a disproportionate effect on this protein class. Conversely, only 40.8% of altered membrane proteins and 33.5% of altered secreted proteins were reduced, with the remainder showing increased levels. Together, these data indicate a distinct ME/CFS serum pattern characterized by reduced abundance of intracellular proteins (Kolmogorov-Smirnov [KS] test: D = 0.080, $p = 1.6 \times 10^{-14}$;

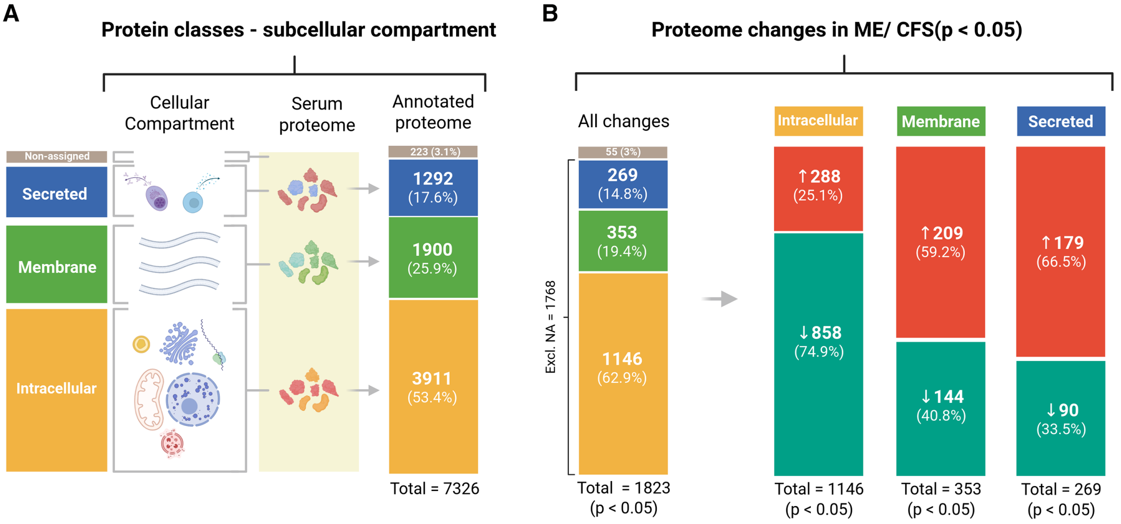

Figure 3. The subcellular origin of the affected proteins
The aptamers were classified into four categories based on the annotated subcellular localization of the targeted proteins (HPA): "intracellular," "membrane" (membrane + membrane-associated), "secreted" proteins, and the remaining unannotated ("non-assigned" [NA], gray bars). The numbers indicate aptamer counts and corresponding percentages of (A) all the aptamers and (B) only the altered aptamers. The percentages in the three subcellular categories (shown on the right) are relative to the total number of assigned aptamers in each class. The red and green bars illustrate the proportions of increases and decreases, respectively.

Hodges-Lehmann estimator [HL] = −0.18 [−0.22 to −0.14]) and increased levels of membrane (KS test: D = 0.089, p = 5.3×10^{-10}, HL = +0.14 [0.10–0.19]) and secreted proteins (KS test: D = 0.123, p = 7.7×10^{-15}; HL = +0.27 [0.22–0.33]), suggesting altered cellular turnover and secretory processes. These relationships were consistent when the comparison was aggregated at the protein level rather than the aptamer level (Document S1: Data S1A, Table S3).

Gene set enrichment analysis of ranked univariate statistics (ME/CFS vs. HC) using GO Biological Process and Reactome terms indicated a broad downregulation of intracellular pathways (Supplemental Data S3). Although this pattern is consistent with the widespread reduction of intracellular protein levels observed in ME/CFS serum, such interpretation may be limited, as these proteins lose their original cellular context once released into the circulation.

Using the MitoCarta 3.0 library,[45] we identified 425 mitochondrial proteins targeted by 445 aptamers in the dataset (Supplemental Data S3). Of these, 20.7% (92 aptamers) showed lower blood concentrations in ME/CFS compared to HC, whereas only 6.7% (30 aptamers) showed higher levels. This indicates a change toward reduced release of mitochondrial proteins into the blood, mirroring the overall pattern observed for intracellular proteins.

Organ and immune cell signatures in the serum proteome

To assess organ contributions to the circulating proteome (Figure 4A), we applied tissue-associated protein panels (HPA), covering 1,670 aptamers targeting 1,428 proteins identified by their "UniProt," of which 20.9% (n = 299; p < 0.05) differed between ME/CFS and HC groups (Supplemental Data S4). Although these panels are not strictly tissue specific, they may still provide meaningful insights into organ-level contributions to the altered serum proteome. Because some proteins were represented in multiple panels, and some were recognized by more than one aptamer, the total number of panel entries exceeded the number of unique proteins. Figure 4B illustrates changes across the protein panels for brain, liver, intestine, skeletal muscle, lymphoid tissue, and bone marrow, which were the most prominent contributors to the altered serum proteome (additional tissues are shown in Supplemental Data S4). In the brain panel, slightly more proteins showed higher rather than lower serum levels in ME/CFS, driven mainly by membrane and secreted proteins, whereas intracellular proteins tended to be reduced. In the liver and intestine panels, a similar pattern was observed, but with less reduction of intracellular proteins. In the skeletal muscle panel, intracellular proteins showed marked reductions, membrane proteins displayed mixed effects, and all affected secreted proteins were increased. In the lymphoid tissue panel, intracellular proteins were reduced, while membrane and secreted proteins were mainly elevated. The bone marrow panel showed a distinct profile, with few increases and widespread reductions, particularly in intracellular and secreted proteins. For the most affected individual proteins, reductions were most pronounced in the panels for brain, skeletal muscle, and bone marrow, whereas liver, intestine, and lymphoid tissues showed comparatively modest, bidirectional alterations (Figure 4C). Overall, these tissue-specific profiles indicate a pattern of reduced intracellular protein release, particularly from skeletal muscle, brain, and bone marrow, accompanied by broadly increased secretion across tissues, suggesting coordinated multi-organ involvement in the pathophysiology of ME/CFS.

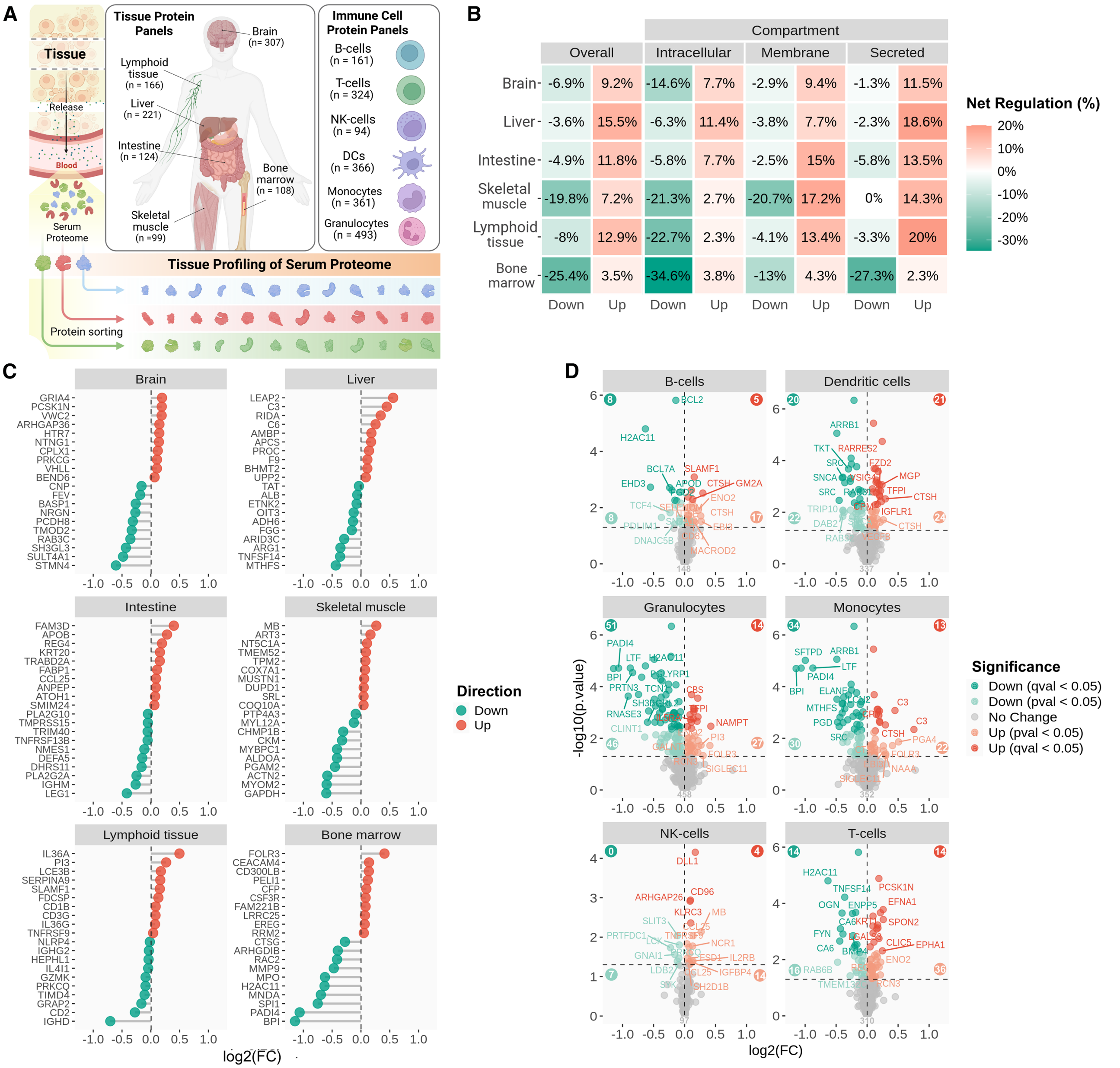

Figure 4. Tissue- and immune cell-associated footprints

(A) Schematic illustration showing the approach of using panels of tissue- and immune cell-associated proteins (HPA) to characterize organ-level contributions to the altered serum proteome. The number of proteins in each panel is shown.

(B) For each protein panel, the proportion of affected proteins (up/down) was calculated relative (%) to the total number of proteins in the panel, and after further division into cellular compartments.

(C) Plots showing the most affected proteins per tissue type, ranked by fold change (log2(FC)).

(D) Volcano plots of immune cell type-associated protein panels. The dotted line indicates −log10(0.05), i.e., the p value significance threshold.

Furthermore, to evaluate effects across immune cell types, six immune-cell-associated protein panels were used (HPA: B cells, dendritic cells, granulocytes, monocytes, natural killer [NK] cells, and T cells). The panels included a total of 1,334 aptamers targeting 1,107 proteins identified by "UniProt" (Figure 4A), and also here, some proteins were represented in more than one panel (Figure 4D; Supplemental Data S4). Across the six panels, the overall fraction of affected aptamers ranged from 20.4% to 23.2% ($p < 0.05$; Document S1: Data S1D, Table S7). Notably, granulocytes and monocytes showed the highest proportion of lowered aptamers (70.3% and 64.6% of changes, respectively), whereas NK cells and T cells showed a predominance of elevated aptamers (72.0% and 62.5% of changes, respectively). These findings suggest a primarily innate-immune-skewed reduction

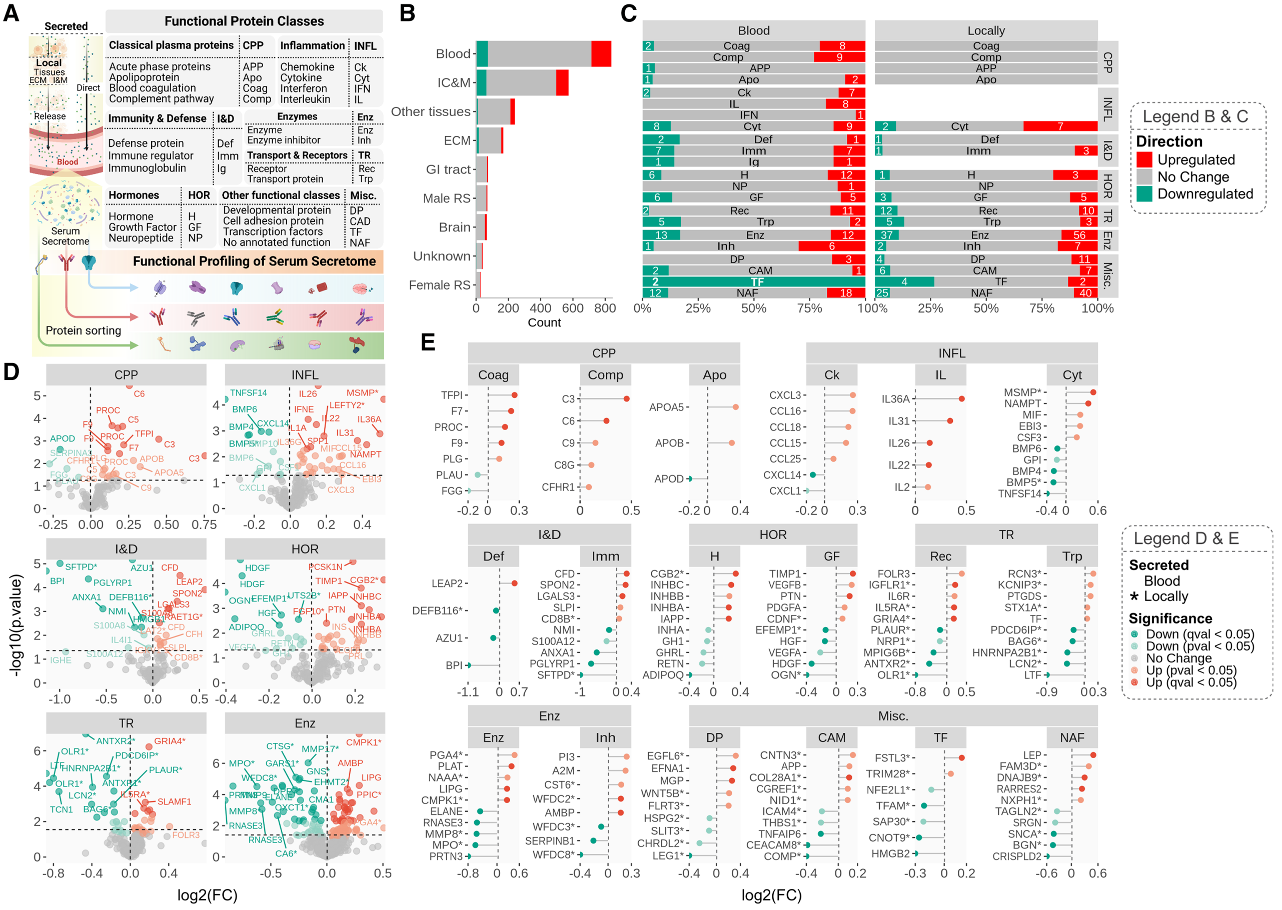

Figure 5. Comparison between the ME/CFS and HC secretome and the related biological processes

(A) The framework for secretome analyses. Secreted proteins were classified according to secretion site (blood vs. locally, tissue-specific) and biological function (abbreviations are shown).[44]

(B) The total and significantly affected aptamer counts across proteins secreted to blood or locally within eight tissue compartments (intracellular and membrane proteins, IC&M; extracellular matrix, ECM; male reproductive system, Male RS; female reproductive system, Female RS; unknown origin, Unknown).

(C) Distribution and direction of change (up, down, unchanged) across functional protein classes, shown as the percentage of all measured proteins within each class (*x* axis).

(D and E) (D) Volcano plots showing all secreted proteins grouped by biological function; colors indicate up- or downregulation and statistical significance. The dotted line indicates $-\log10(0.05)$, i.e., the *p* value significance threshold. (E) Plots highlighting the most affected proteins within each biological function, ranked by fold change ($\log_2$FC).

in granulocyte- and monocyte-associated signatures, contrasting with more mixed or upward changes in lymphoid cell panels.

To further characterize the observed granulocyte/neutrophil-associated signature, we compared our dataset with a published list of proteins released by phorbol 12-myristate 13-acetate (PMA)-activated neutrophils[33] (Supplemental Data S3). Of the 254 listed proteins, 146 were detected in our dataset, represented by 172 aptamers, of which 52.3% showed differences between ME/CFS and HC groups (90 aptamers at $p < 0.05$; 62 aptamers, 36.0% at q < 0.05). Notably, more than 85% of the affected aptamers (79 aptamers at $p < 0.05$; 57 aptamers at q < 0.05) showed lower levels in ME/CFS, indicating a broad and pronounced reduction in circulating neutrophil-derived proteins, including several hallmark granule components such as BPI, PADI4, MMP9, ELANE, PRTN3, AZU1, and LTF. This suppressed neutrophil signature was not accompanied by abnormal granulocyte/neutrophil counts in patients (3.38 ± 1.03 × 10^9/L; normal range 1.5–7.3 × 10^9/L), and other blood cell counts were also within normal limits (Document S1: Data S1E, Table S8).

Secretory processes and functional changes

To further investigate the apparent stimulation of secretory processes in ME/CFS, we utilized an optimized protein-centric database of the predicted human secretome,[44] which annotates both tissue-specific expression and functional roles of secreted proteins (Figure 5A; Supplemental Data S5). Based on 2,100 aptamers recognizing 1,657 proteins ("UniProt") of the secretome, 21.6% of the aptamers showed difference between ME/CFS and HC groups (n = 454 at $p < 0.05$; n = 185, 8.8% at q < 0.05), with the majority showing increased levels

(61.2% of changes at $p < 0.05$; 54.6% of changes at $q < 0.05$). The annotation categorized secreted proteins into nine functional groups, including blood proteins (Blood) and proteins secreted locally in eight compartments: intracellular and membrane proteins (IC&M), other tissues, extracellular matrix (ECM), gastrointestinal tract (GI tract), male reproductive system (Male RS), brain, unknown origin (Unknown), and female reproductive system (Female RS). Figure 5B shows a widespread pattern of increased levels across these secretome categories, supporting elevated activity involving multiple organ systems and physiological processes.

Within the largest class, blood proteins, 197 of the 838 aptamers showed significant changes, with 63% ($n = 124$) at higher levels and 37% ($n = 73$) at lower levels. Many of these circulating proteins originate from the liver, while others are produced by blood cells or more broadly across tissues.[44] Functional annotation indicated mixed impacts across classical plasma proteins (CPP), enzymes (Enz), hormones (HOR), immunity and defense (I&D), inflammation (INFL), and transport and receptors (TR), as well as their subcategories, which reflects distinct biological processes (Figure 5C). Figure 5D shows all the affected aptamers across secretome categories according to function, whereas Figure 5E provides the most affected proteins per functional (sub)category. These data show a pattern of increased secretion of proteins involved in coagulation, complement pathways, and inflammation (chemokines and ILs). In contrast, the enzyme class predominantly showed lower serum levels, which partly relate to the overall decreased release of intracellular proteins in general (Figure 3) and the observed neutrophil signature (Figure 4). The other secretome classes, including hormones, immunity and defense, transport and receptors, and others, expressed mixed effects.

We performed a ligand-receptor interaction analysis to explore potential signaling relationships reflected in the proteomic data (Document S1: Data S1F, Figure S4). This approach identifies pairs of ligands and receptors whose levels change in a coordinated manner, which may indicate altered intercellular communication. The analysis revealed an over-representation of interactions within the cell adhesion and cytokine-cytokine receptor categories. Among these, four members of the Ephrin subfamily A receptors (EPHA1, EPHA2, EPHA4, and EPHA7) and their ligands (EFNA proteins) showed concordant changes, consistent with previously suggested involvement of EPHA-EFNA signaling in ME/CFS.[29] Additional examples included concordance of FGFR3 with EFNA ligands, IL6R with CNTF and MPZ, and FLRT3 with UNC5B/UNC5D, whereas FAP and PAM showed negative concordance. While these findings do not establish causality, they highlight signaling pathways that may contribute to metabolic regulation, tissue development and repair, inflammation, or angiogenesis in ME/CFS.

Validation and expansion of serum proteome findings using antibody-based methods

To validate and extend the aptamer-based findings, we measured a panel of serum proteins related to immunity, inflammation, coagulation, and energy-stress metabolism using antibody-based assays (ELISA and Luminex). Samples from 83 ME/CFS patients and 29 HCs (Document S1: Data S1G, Table S9) were analyzed for 77 proteins using the Luminex platform. After excluding 23 proteins with low abundance (defined as >50% missing data), 54 proteins were retained for statistical analysis (Supplemental Data S6). Group-level concordance analysis compared the ME/CFS-associated changes in these 54 Luminex proteins with the 68 corresponding aptamers on the SomaScan platform (Figure 6A; Supplemental Data S6; Document S1, Data S1H). The majority of proteins (70.4%) showed concordant effects across the two platforms; 7.4% showed mixed results because different aptamers targeting the same protein reported divergent changes (notably BDNF, COL1A1, OSM, and osteoactivin) and 22.2% showed discordant results. For signal-consistency analysis, 69.1% of platform comparisons demonstrated a significant positive correlation ($p < 0.05$) and none showed negative correlation (Figure 6B; Supplemental Data S6). In summary, most significant group-level differences between ME/CFS and HC groups were reproduced using antibody-based measurements, thereby supporting the overall findings obtained with aptamer-based detection.

A selection of significant differences between ME/CFS and HC groups based on the Luminex data is shown in the heatmap in Figure 6C, which also compares the serum protein levels across metabotypes. Corresponding data for all the measured proteins are provided in Supplemental Data S6. Metabotype-specific differences were shown particularly for metabolic hormones, such as the high FABP4 level seen in both metabotypes 1 and 2 and the high insulin and leptin levels in metabotype 2. The group-level effects on immune system-related proteins generally remained relatively similar between the metabotype subgroups, such as reduced MPO and TGFα. Two exceptions were CCL24, which expressed a specific increase in the metabotype 1 subgroup, and IL-8, which specifically showed a lower level in the metabotype 3 subgroup.

We performed additional conventional ELISA measurements of two metabolic stress hormones, fibroblast growth factor (FGF)-21 and growth differentiation factor (GDF)-15, in addition to C-peptide, a marker of insulin production, using an extended cohort ($n = 212$ ME/CFS, $n = 66$ HC) (Figures 6D–6F). FGF-21 and C-peptide were not included in the previous aptamer-based analysis. For FGF-21, the serum concentration was higher in the ME/CFS group compared to HC (Figure 6D). When stratified by sex, this difference was statistically significant in women but not in men. The FGF-21 level differed across the three ME/CFS metabotype subsets, with metabotype 2 showing the highest concentration. For GDF-15 and C-peptide, the ELISA analysis showed no overall difference between ME/CFS and HC groups, irrespective of sex (Figures 6E and 6F); however, both markers were higher in metabotype 2 compared to metabotypes 1 and 3.

DISCUSSION

This serum proteomics study identified contextual differences in circulatory protein concentrations between ME/CFS patients and healthy individuals. Through the investigations, we aimed to position the observed patterns within the mechanistic landscape, as summarized in Figure 7.

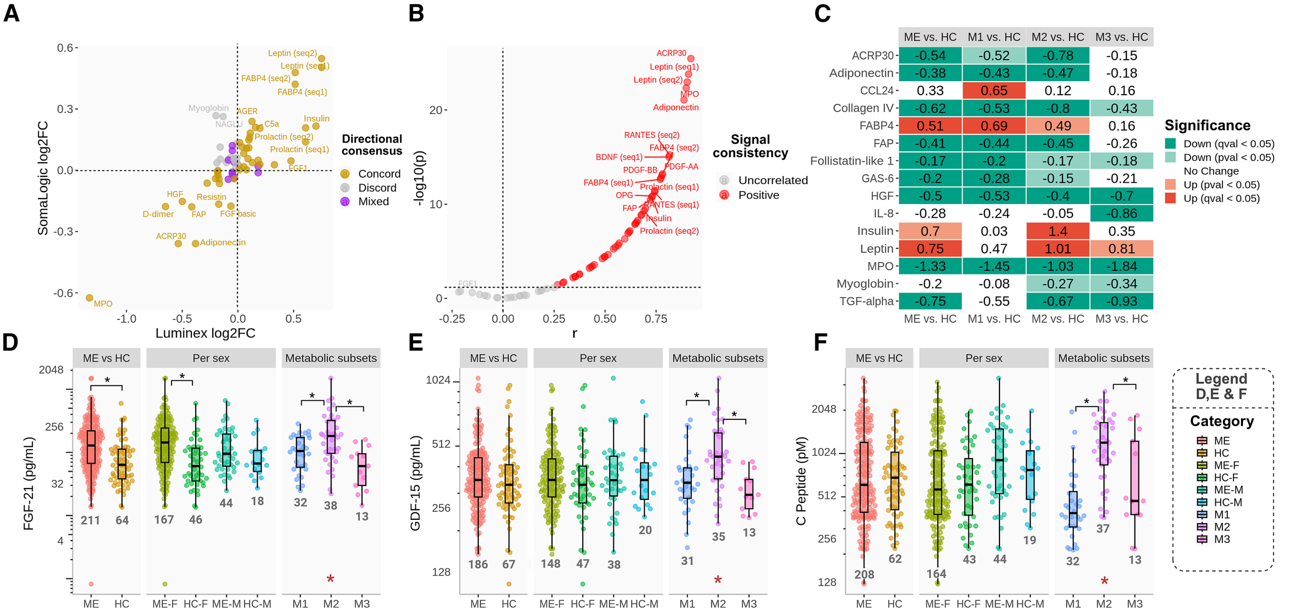

Figure 6. Targeted validation and expansion of key findings

Antibody-based measurements (Luminex and ELISA) were used to validate and expand on key outcomes from aptamer-based serum proteomics. Luminex data are shown for a panel of 54 serum proteins related to immunity and metabolism, in 83 ME/CFS patients and 29 HC subjects. Samples were analyzed in single assay wells, with intra- and inter-plate technical replicates included for designated samples.

(A and B) (A) The directional concordance, and (B) signal consistency (Pearson correlation), between antibody-based and the aptamer-based measurements, per protein.

(C–F) (C) The heatmap displays selected affected proteins, including comparison between the three ME/CFS metabotypes (M1, M2, and M3). Expanded ELISA analysis of (D) FGF-21, (E) GDF-15, and (F) C-peptide was performed in larger cohorts (n = 212 ME/CFS, n = 66 HC). Each sample was assayed in technical duplicate. Boxplot indicates median and IQR (25th–75th percentiles), and the whiskers indicate ±1.5×IQR. Statistical comparisons were performed between groups (Welch's t test with FDR correction, q < 0.05, black asterisks) and stratified by sex and metabotype (one-way ANOVA, $p < 0.05$, red asterisks, followed by post hoc Welch's t test with FDR correction, q < 0.05, black asterisks).

We applied two complementary strategies to gain multiscale insights into ME/CFS pathology. First, we interrogated the full serum proteome to reveal systemic, tissue-associated, and immune-cell-related alterations. Second, we focused specifically on secreted proteins to examine the effects on functional and regulatory physiological processes. Analyses confirmed that sex, age, BMI, and fasting status did not account for the broad proteomic differences between ME/CFS and HC groups. Moreover, the extensive changes found to be unrelated to physical function and activity (SF-36PF, mean steps) argue against deconditioning as the primary driver of the ME/CFS proteome. Rather, it may be relevant to elucidate proteins linking exertion-triggered effects to immunological and metabolic dysregulation, which may potentially track with hallmark symptoms of ME/CFS, such as fatigue and PEM.[46]

Comparison with other proteomic studies

To compare our results with previous work using the same SomaScan platform, we reanalyzed data from Germain et al.[29], and Walitt et al.[29,30] using a similar analytical pipeline adapted to the available data (STAR Methods; Supplemental Data S7; Document S1: Data S1I, Figures S5 and S6; Tables S10–S13). In the Germain dataset, 391 aptamers differed between ME/CFS and controls ($p < 0.05$), of which 122 overlapped with our findings. In the Walitt dataset, 41 aptamers were altered, with seven overlapping with our data. While neither dataset demonstrated the broad reduction in intracellular proteins observed here, the Germain data showed a relative increase in membrane and secreted proteins, consistent with our results. Despite some inter-study variation, likely reflecting differences in ME/CFS case definitions and cohort size, all three studies consistently pointed to immune-related dysregulation. Our results also align with other independent proteomic investigations showing immune-vascular dysregulation and metabolic involvement, including coagulation and complement pathway changes,[47] immune-related differences in plasma and extracellular vesicles,[48] mitochondrial and metabolic pathway changes in immune cells,[49,50] and aberrant innate/adaptive immune regulation revealed by single-cell and multi-omics analyses.[51] Together, these cross-platform findings converge on immune, vascular, and metabolic dysregulation in ME/CFS and strengthen our interpretation of the serum proteome results.

Reduced protein release from muscle

The reduced release of intracellular skeletal muscle proteins appears to represent a distinct ME/CFS phenotype that is supported by our data. This is unlikely to reflect potential dilution effects from altered blood volume,[52] given the tissue- and pathway-specific nature of the overall changes. Although decreased leakage of muscle proteins could theoretically result

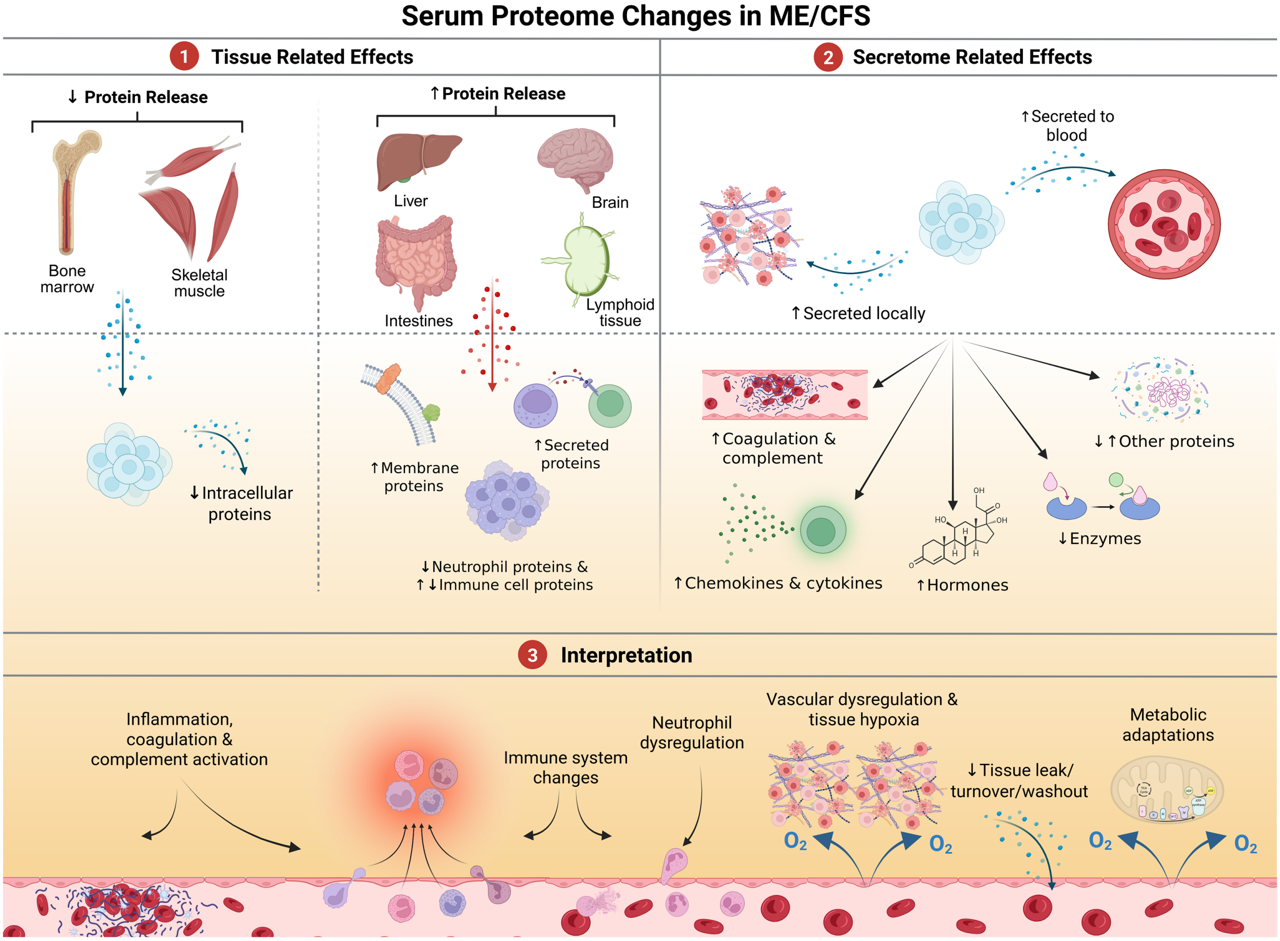

Figure 7. Impacts of serum proteome changes in ME/CFS

This schematic summarizes key findings: (1) *Tissue-related effects*: ME/CFS patients showed reduced blood levels of intracellular proteins, most pronounced for skeletal muscle and bone marrow, alongside increased levels of membrane-associated and secreted proteins from brain, intestine, liver, skeletal muscle, and lymphoid tissues. A substantial reduction of proteins released by activated neutrophils contrasted with more variable or elevated lymphoid-cell patterns (2) *Secretome effects*: Overall secretory activity was elevated, with increased levels of coagulation proteins, complement factors, chemokines, cytokines, and hormones, while enzyme levels were reduced. (3) *Interpretation*: These patterns suggest underlying immune dysregulation, possibly involving autoimmune mechanisms, with downstream roles of low-grade inflammation, tissue hypoperfusion, and metabolic disturbance.

from low physical activity or reduced muscle mass, this explanation is not well supported, as the analyses were adjusted for age, BMI, and sex, which partly account for such effects. While the underlying mechanisms remain unclear, it seems likely that muscle tissue homeostasis and turnover are affected, potentially linked to previously reported muscle abnormalities and contributing to hallmark ME/CFS symptoms such as fatigue and PEM.[18,53–55]

Secretome effects and functional impact

We observed an overall increase of secretory activity in ME/CFS patients, with 62% of affected secreted proteins showing elevated levels. In particular, the affected proteins indicate changes related to coagulation, complement activation, chemokines, ILs, hormones, enzyme inhibitors, and receptors, consistent with previous reports of elevated inflammatory cytokines in ME/CFS, although some variation exists across studies.[22,56,57] We also noted increases in tissue-derived stress messengers such as FABP types, which function intracellularly as fatty acid transporters but act in serum as markers of metabolic stress.[58] Taken together, the pattern of heightened systemic secretory activity aligns with a chronic state of immune, vascular, and metabolic dysregulation.

Immune system changes and impaired neutrophil function

Our findings indicate a primarily innate-immune-skewed reduction in granulocyte- and monocyte-associated signatures, contrasting with more variable or elevated lymphoid-cell patterns. This imbalance may reflect a disturbed immune homeostasis that could contribute to or parallel autoimmune-like mechanisms, although the present data do not directly demonstrate autoimmunity. Increased pro-inflammatory cytokines associated with T cell dysregulation, together with mixed changes reflecting disturbed immune balance, are hallmarks of many

autoimmune conditions.[59] The increased inflammatory, coagulation, and complement factors align with previous observations in ME/CFS and long COVID.[1,60,61] The substantial reduction of neutrophil-associated proteins may indicate impaired neutrophil maturation or activation.[62,63] Elevated complement factors CFD and C6 indicate alternative complement pathway activation, implicated in vasculitis, lupus, dermatomyositis, and autoimmune nephritis.[64] Reduced MPO and extracellular histones, which are autoantigens in antineutrophil cytoplasmic antibodies (ANCA)-associated vasculitis, further suggest aberrant neutrophil-complement interplay, although ANCA is not typically reported in ME/CFS.[65] Additional evidence includes the presence of autoantibodies and promising studies of antibody-targeting therapies such as immune adsorption and plasma-cell-directed treatments.[16,66] Collectively, these findings support a possible autoimmune contribution to ME/CFS pathology.

Vascular dysregulation and hypoxia response

Several proteins linked to microcirculatory and hypoxia responses were altered in ME/CFS. Reduced levels of VIM, ANTXR2, CXADR, and ATP5IF1 may indicate impaired endothelial regulation and mitochondrial stress protection during oxygen deprivation.[34–37] In contrast, the endothelial lipase LIPG, known to be induced by immune activation,[42,43] and the hematopoietic signaling factor FAM20A[67] were increased. Endothelial dysfunction, repeatedly reported in ME/CFS,[68–70] can impair vascular tone, oxygen delivery, immune interactions, and metabolism through disrupted microcirculatory signaling.[71–73] Similar capillary alterations and hypoxia-related changes are described in long COVID,[74–76] and ME/CFS patients showed increased WASF3 expression, a target of the hypoxia-regulated transcription factor HIF1A.[77] Together, these findings support a model in which endothelial dysfunction, impaired vascular flow, and tissue hypoxia contribute to the pathophysiology of ME/CFS.

Metabolic stress and energy metabolism

Our results support prior evidence of metabolic stress signaling in context of ME/CFS, including elevated GDF-15,[78] FGF-21, resistin, leptin, and hepatocyte growth factor (HGF).[22,56] FGF-21 and GDF-15, which are stress hormones also elevated in mitochondrial myopathies, type 2 diabetes, and hypoxia, were particularly increased in the previously defined metabotype 2 subgroup, alongside higher C-peptide and leptin, while FABP4 was elevated in both metabotypes 1 and 2.[17] These so-called exerkines (e.g., FGF-21 and GDF-15) normally rise transiently during exercise to coordinate systemic energy adaptation.[79] Their chronic elevation in ME/CFS suggests persistent energy strain, which could reflect or exacerbate impaired oxygen delivery and utilization,[80] as supported by findings in ME/CFS,[81] long COVID,[82] and metabolic myopathies,[83] and may contribute to PEM and reduced exercise tolerance.

Perspectives

There are several ways in which we believe this work could support new research directions. First, it identifies specific cells, organs, and processes affected in ME/CFS, which may guide the development of diagnostic markers and therapeutic strategies. These insights may also be relevant to related syndromes, such as long COVID and fibromyalgia. Second, it provides a mechanistic landscape that can be used to contextualize previous and future findings within a broader (patho)physiological framework, e.g., in relation to potential risk factors identified through multi-omics analyses.[84,85] Third, it may inform the development of relevant laboratory models for ME/CFS, including through systemic comparisons[86] and *in vitro* recapitulation of key mechanistic features.[87]

In conclusion, the serum proteome in ME/CFS reveals a distinct pattern characterized by reduced release of intracellular proteins and increased secretion of extracellular proteins, indicating widespread tissue involvement and disturbed systemic homeostasis. These changes point to altered metabolic and inflammatory regulation across multiple organs, including skeletal muscle, brain, and bone marrow. The accompanying immune dysregulation and attenuated neutrophil function, further supports the notion of a sustained, multisystem imbalance involving immune, vascular, and metabolic processes. Together, these findings offer molecular insight into ME/CFS pathophysiology and a foundation for future biomarker and therapeutic development.

Limitations of the study

Aptamer-based proteomics offers broad, sensitive, and highly multiplex profiling that is relatively robust to protein degradation and pre-analytical variation,[88] although it remains semi-quantitative and provides lower resolution than mass spectrometry or antibody-based platforms for detecting isoforms, proteoforms, or post-translational modifications.[89] Therefore, findings of interest should be verified using additional high-confidence methods. The sample size was primarily limited by the availability of well-characterized biobanked serum samples from two clinical trials and the high cost of large-scale SomaScan 7k profiling. While modest in size, this study is comparable in size to other studies in the field, providing a basis for interpretation. Future studies in larger, independent cohorts will be important to replicate and extend these findings. When using protein annotations to track functions and tissue locations, it is important to be aware that few proteins are exclusively specific to a given process or organ and may play different context-dependent roles. Therefore, alternative interpretations of the findings may be relevant. We have mitigated some of these limitations by primarily focusing our interpretations on patterns of changes related to different tissues and processes, rather than single protein effects.

RESOURCE AVAILABILITY

Lead contact

Further information and requests for resources and reagents should be directed to and will be fulfilled by the lead contact, Karl Johan Tronstad (karl.tronstad@uib.no).

Materials availability

This study did not generate new unique reagents.

Data and code availability

- The analyzed proteomics data are included as supplemental information. The original dataset is deposited in the PRIDE[90] repository (PRIDE: PAD000026).

- Any additional information required to reanalyze the data reported in this paper is available from the lead contact upon request.
- This paper does not generate original code.

ACKNOWLEDGMENTS

The authors thank the study personnel at each center of the "RituxME" and "CycloME" clinical trials for their efforts in patient follow-up and biobank sampling. This work received financial support from The Kavli Trust, the Research Council of Norway (KJT, projects 272680 and 343168), the Western Norway Regional Health Authority (Helse Vest), and the University of Bergen (PhD grants to F.H. and A.H.). The clinical trials that recruited patients for the biobank and the laboratory analyses in this study were financially supported by the Research Council of Norway, the Norwegian Regional Health Trusts, the MEandYou Foundation, the Norwegian ME Association, and the legacy of Torstein Hereid. Additionally, we are grateful for the support from private donors and the Norwegian ME Association for laboratory research on ME/CFS.

AUTHOR CONTRIBUTIONS

A.H., F.H., H.C., Ø.F., O.M., and K.J.T. designed the analytical approach. Ø.F., O.M., I.G.R., K.S., K.R., and K.A. included patients in clinical studies and provided biobank samples and data. F.H., S.E.D., and A.H. performed laboratory measurements. K.A.B. and H.-P.M. provided scientific and technical advice. A.H., F.H., H.C., K.J.T., and Ø.F. conducted data analyses. A.H., F.H., H.C., and K.J.T. wrote the first version of manuscript. All authors approved the final version of the manuscript.

DECLARATION OF INTERESTS

The authors have declared that no conflict of interest exists related to this work.

DECLARATION OF GENERATIVE AI AND AI-ASSISTED TECHNOLOGIES IN THE WRITING PROCESS

During the preparation of this manuscript, the authors used Microsoft Copilot (UiB institutional license) and ChatGPT5 to assist with translation and ensure readability. After using this tool/service, the authors reviewed and edited the content as needed and take full responsibility for the content of the published article.

STAR★METHODS

Detailed methods are provided in the online version of this paper and include the following:

- KEY RESOURCES TABLE
- EXPERIMENTAL MODEL AND STUDY PARTICIPANT DETAILS
  - Human participants
  - Ethics statement
- METHOD DETAILS
  - SomaLogic proteomic profiling
  - Alignment of aptamer pairs
  - Subcellular and immune annotation
  - CycloME responder analysis
  - Activity and clinical correlates
  - Luminex analysis
  - ELISA
  - Interstudy comparison
- QUANTIFICATION AND STATISTICAL ANALYSIS
- ADDITIONAL RESOURCES

SUPPLEMENTAL INFORMATION

Supplemental information can be found online at https://doi.org/10.1016/j.xcrm.2026.102647.

Received: May 28, 2025
Revised: October 28, 2025
Accepted: February 2, 2026
Published: March 4, 2026

REFERENCES

1. Choutka, J., Jansari, V., Hornig, M., and Iwasaki, A. (2022). Unexplained post-acute infection syndromes. Nat. Med. *28*, 911–923. https://doi.org/10.1038/s41591-022-01810-6.
2. Komaroff, A.L. (2025). Growing recognition of post-acute infection syndromes. Proc. Natl. Acad. Sci. USA *122*, e2513877122. https://doi.org/10.1073/pnas.2513877122.
3. Davis, H.E., McCorkell, L., Vogel, J.M., and Topol, E.J. (2023). Long COVID: major findings, mechanisms and recommendations. Nat. Rev. Microbiol. *21*, 133–146. https://doi.org/10.1038/s41579-022-00846-2.
4. Jason, L.A., Yoo, S., and Bhatia, S. (2022). Patient perceptions of infectious illnesses preceding Myalgic Encephalomyelitis/Chronic Fatigue Syndrome. Chron. Illness *18*, 901–910. https://doi.org/10.1177/17423953211043106.
5. Rasa, S., Nora-Krukle, Z., Henning, N., Eliassen, E., Shikova, E., Harrer, T., Scheibenbogen, C., Murovska, M., and Prusty, B.K.; European Network on ME/CFS EUROMENE (2018). Chronic viral infections in myalgic encephalomyelitis/chronic fatigue syndrome (ME/CFS). J. Transl. Med. *16*, 268. https://doi.org/10.1186/s12967-018-1644-y.
6. Carruthers, B.M., Van De Sande, M.I., De Meirleir, K.L., Klimas, N.G., Broderick, G., Mitchell, T., Staines, D., Powles, A.C.P., Speight, N., Vallings, R., et al. (2011). Myalgic encephalomyelitis: International Consensus Criteria. J. Intern. Med. *270*, 327–338. https://doi.org/10.1111/j.1365-2796.2011.02428.x.
7. Jason, L.A., Katz, B.Z., Sunnquist, M., Torres, C., Cotler, J., and Bhatia, S. (2020). The Prevalence of Pediatric Myalgic Encephalomyelitis/Chronic Fatigue Syndrome in a Community-Based Sample. Child Youth Care Forum *49*, 563–579. https://doi.org/10.1007/s10566-019-09543-3.
8. Nacul, L.C., Lacerda, E.M., Pheby, D., Campion, P., Molokhia, M., Fayyaz, S., Leite, J.C.D.C., Poland, F., Howe, A., and Drachler, M.L. (2011). Prevalence of myalgic encephalomyelitis/chronic fatigue syndrome (ME/CFS) in three regions of England: a repeated cross-sectional study in primary care. BMC Med. *9*, 91. https://doi.org/10.1186/1741-7015-9-91.
9. Valdez, A.R., Hancock, E.E., Adebayo, S., Kiernicki, D.J., Proskauer, D., Attewell, J.R., Bateman, L., Demaria, A., Lapp, C.W., Rowe, P.C., and Proskauer, C. (2018). Estimating Prevalence, Demographics, and Costs of ME/CFS Using Large Scale Medical Claims Data and Machine Learning. Front. Pediatr. *6*, 412. https://doi.org/10.3389/fped.2018.00412.
10. Rowe, P.C., Fontaine, K.R., Lauver, M., Jasion, S.E., Marden, C.L., Moni, M., Thompson, C.B., and Violand, R.L. (2016). Neuromuscular Strain Increases Symptom Intensity in Chronic Fatigue Syndrome. PLoS One *11*, e0159386. https://doi.org/10.1371/journal.pone.0159386.
11. Carruthers, B.M., Jain, A.K., De Meirleir, K.L., Peterson, D.L., Klimas, N.G., Lerner, A.M., Bested, A.C., Flor-Henry, P., Joshi, P., Powles, A.C.P., et al. (2003). Myalgic Encephalomyelitis/Chronic Fatigue Syndrome. J. Chronic Fatigue Syndrome *11*, 7–115. https://doi.org/10.1300/j092v11n01_02.
12. Falk Hvidberg, M., Brinth, L.S., Olesen, A.V., Petersen, K.D., and Ehlers, L. (2015). The Health-Related Quality of Life for Patients with Myalgic Encephalomyelitis/Chronic Fatigue Syndrome (ME/CFS). PLoS One *10*, e0132421. https://doi.org/10.1371/journal.pone.0132421.
13. Pheby, D.F.H., Araja, D., Berkis, U., Brenna, E., Cullinan, J., De Korwin, J.-D., Gitto, L., Hughes, D.A., Hunter, R.M., Trepel, D., and Wang-Steverding, X. (2020). The Development of a Consistent Europe-Wide Approach to

Investigating the Economic Impact of Myalgic Encephalomyelitis (ME/CFS): A Report from the European Network on ME/CFS (EUROMENE). Healthcare *8*, 88. https://doi.org/10.3390/healthcare8020088.

14. Kielland, A., and Liu, J. (2025). What can wage development before and after a G93.3 diagnosis tell us about prognoses for myalgic encephalomyelitis? Soc. Sci. Humanit. Open *11*, 101206.

15. Komaroff, A.L. (2019). Advances in Understanding the Pathophysiology of Chronic Fatigue Syndrome. JAMA *322*, 499–500. https://doi.org/10.1001/jama.2019.8312.

16. Fluge, Ø., Tronstad, K.J., and Mella, O. (2021). Pathomechanisms and possible interventions in myalgic encephalomyelitis/chronic fatigue syndrome (ME/CFS). J. Clin. Investig. *131*, e150377. https://doi.org/10.1172/jci150377.

17. Hoel, F., Hoel, A., Pettersen, I.K., Rekeland, I.G., Risa, K., Alme, K., Sørland, K., Fosså, A., Lien, K., Herder, I., et al. (2021). A map of metabolic phenotypes in patients with myalgic encephalomyelitis/chronic fatigue syndrome. JCI Insight *6*, e149217. https://doi.org/10.1172/jci.insight.149217.

18. Appelman, B., Charlton, B.T., Goulding, R.P., Kerkhoff, T.J., Breedveld, E.A., Noort, W., Offringa, C., Bloemers, F.W., Van Weeghel, M., Schomakers, B.V., et al. (2024). Muscle abnormalities worsen after post-exertional malaise in long COVID. Nat. Commun. *15*, 17. https://doi.org/10.1038/s41467-023-44432-3.

19. Uhlen, M., Fagerberg, L., Hallstrom, B.M., Lindskog, C., Oksvold, P., Mardinoglu, A., Sivertsson, A., Kampf, C., Sjostedt, E., Asplund, A., et al. (2015). Proteomics. Tissue-based map of the human proteome. Science *347*, 1260419. https://doi.org/10.1126/science.1260419.

20. Jiang, L., Wang, M., Lin, S., Jian, R., Li, X., Chan, J., Dong, G., Fang, H., Robinson, A.E., and GTEx Consortium; and Snyder, M.P. (2020). A Quantitative Proteome Map of the Human Body. Cell *183*, 269–283.e19. https://doi.org/10.1016/j.cell.2020.08.036.

21. Anderson, N.L., and Anderson, N.G. (2002). The Human Plasma Proteome. Mol. Cell. Proteomics *1*, 845–867. https://doi.org/10.1074/mcp.r200007-mcp200.

22. Hornig, M., Montoya, J.G., Klimas, N.G., Levine, S., Felsenstein, D., Bateman, L., Peterson, D.L., Gottschalk, C.G., Schultz, A.F., Che, X., et al. (2015). Distinct plasma immune signatures in ME/CFS are present early in the course of illness. Sci. Adv. *1*, e1400121. https://doi.org/10.1126/sciadv.1400121.

23. Lunde, S., Kristoffersen, E.K., Sapkota, D., Risa, K., Dahl, O., Bruland, O., Mella, O., and Fluge, Ø. (2016). Serum BAFF and APRIL Levels, T-Lymphocyte Subsets, and Immunoglobulins after B-Cell Depletion Using the Monoclonal Anti-CD20 Antibody Rituximab in Myalgic Encephalopathy/Chronic Fatigue Syndrome. PLoS One *11*, e0161226. https://doi.org/10.1371/journal.pone.0161226.

24. Cambras, T., Zerón-Rugerio, M.F., Díez-Noguera, A., Zaragozá, M.C., Domingo, J.C., Sanmartin-Sentañes, R., Alegre-Martin, J., and Castro-Marrero, J. (2023). Skin Temperature Circadian Rhythms and Dysautonomia in Myalgic Encephalomyelitis/Chronic Fatigue Syndrome: The Role of Endothelin-1 in the Vascular Tone Dysregulation. Int. J. Mol. Sci. *24*, 4835. https://doi.org/10.3390/ijms24054835.

25. Baklund, I.H., Dammen, T., Moum, T.Å., Kristiansen, W., Duarte, D.S., Castro-Marrero, J., Helland, I.B., and Strand, E.B. (2021). Evaluating Routine Blood Tests According to Clinical Symptoms and Diagnostic Criteria in Individuals with Myalgic Encephalomyelitis/Chronic Fatigue Syndrome. J. Clin. Med. *10*, 3105. https://doi.org/10.3390/jcm10143105.

26. Gold, L., Ayers, D., Bertino, J., Bock, C., Bock, A., Brody, E.N., Carter, J., Dalby, A.B., Eaton, B.E., Fitzwater, T., et al. (2010). Aptamer-Based Multiplexed Proteomic Technology for Biomarker Discovery. PLoS One *5*, e15004. https://doi.org/10.1371/journal.pone.0015004.

27. Lehallier, B., Gate, D., Schaum, N., Nanasi, T., Lee, S.E., Yousef, H., Moran Losada, P., Berdnik, D., Keller, A., Verghese, J., et al. (2019). Undulating changes in human plasma proteome profiles across the lifespan. Nat. Med. *25*, 1843–1850. https://doi.org/10.1038/s41591-019-0673-2.

28. Niewczas, M.A., Pavkov, M.E., Skupien, J., Smiles, A., Md Dom, Z.I., Wilson, J.M., Park, J., Nair, V., Schlafly, A., Saulnier, P.-J., et al. (2019). A signature of circulating inflammatory proteins and development of end-stage renal disease in diabetes. Nat. Med. *25*, 805–813. https://doi.org/10.1038/s41591-019-0415-5.

29. Germain, A., Levine, S.M., and Hanson, M.R. (2021). In-Depth Analysis of the Plasma Proteome in ME/CFS Exposes Disrupted Ephrin-Eph and Immune System Signaling. Proteomes *9*, 6. https://doi.org/10.3390/proteomes9010006.

30. Walitt, B., Singh, K., Lamunion, S.R., Hallett, M., Jacobson, S., Chen, K., Enose-Akahata, Y., Apps, R., Barb, J.J., Bedard, P., et al. (2024). Deep phenotyping of post-infectious myalgic encephalomyelitis/chronic fatigue syndrome. Nat. Commun. *15*, 907. https://doi.org/10.1038/s41467-024-45107-3.

31. Fluge, Ø., Rekeland, I.G., Lien, K., Thürmer, H., Borchgrevink, P.C., Schäfer, C., Sørland, K., Aßmus, J., Ktoridou-Valen, I., Herder, I., et al. (2019). B-Lymphocyte Depletion in Patients With Myalgic Encephalomyelitis/Chronic Fatigue Syndrome. Ann. Intern. Med. *170*, 585–593. https://doi.org/10.7326/m18-1451.

32. Rekeland, I.G., Fosså, A., Lande, A., Ktoridou-Valen, I., Sørland, K., Holsen, M., Tronstad, K.J., Risa, K., Alme, K., Viken, M.K., et al. (2020). Intravenous Cyclophosphamide in Myalgic Encephalomyelitis/Chronic Fatigue Syndrome. An Open-Label Phase II Study. Front. Med. *7*, 162. https://doi.org/10.3389/fmed.2020.00162.

33. Petretto, A., Bruschi, M., Pratesi, F., Croia, C., Candiano, G., Ghiggeri, G., and Migliorini, P. (2019). Neutrophil extracellular traps (NET) induced by different stimuli: A comparative proteomic analysis. PLoS One *14*, e0218946. https://doi.org/10.1371/journal.pone.0218946.

34. Päll, T., Pink, A., Kasak, L., Turkina, M., Anderson, W., Valkna, A., and Kogerman, P. (2011). Soluble CD44 Interacts with Intermediate Filament Protein Vimentin on Endothelial Cell Surface. PLoS One *6*, e29305. https://doi.org/10.1371/journal.pone.0029305.

35. Cryan, L.M., Tsang, T.-M., Stiles, J., Bazinet, L., Lee, S.L., Garrard, S., Madrian, E., Roberts, C., Payne, J., Jensen, A., et al. (2022). Capillary morphogenesis gene 2 (CMG2) mediates growth factor-induced angiogenesis by regulating endothelial cell chemotaxis. Angiogenesis *25*, 397–410. https://doi.org/10.1007/s10456-022-09833-w.

36. Chung, J., Kim, K.H., An, S.H., Lee, S., Lim, B.-K., Kang, S.W., and Kwon, K. (2019). Coxsackievirus and adenovirus receptor mediates the responses of endothelial cells to fluid shear stress. Exp. Mol. Med. *51*, 1–15. https://doi.org/10.1038/s12276-019-0347-7.

37. Gatto, C., Grandi, M., Solaini, G., Baracca, A., and Giorgio, V. (2022). The F1Fo-ATPase inhibitor protein IF1 in pathophysiology. Front. Physiol. *13*, 917203. https://doi.org/10.3389/fphys.2022.917203.

38. Chen, X., Wang, Y., Li, Y., Liu, G., Liao, K., and Song, F. (2022). Identification of immune-related cells and genes in the breast invasive carcinoma microenvironment. Aging *14*, 1374–1388. https://doi.org/10.18632/aging.203879.

39. Qi, P., Huang, M., and Li, T. (2022). Screening the Potential Biomarkers of COVID-19-Related Thrombosis Through Bioinformatics Analysis. Front. Genet. *13*, 889348. https://doi.org/10.3389/fgene.2022.889348.

40. Hotamisligil, G.S., and Bernlohr, D.A. (2015). Metabolic functions of FABPs—mechanisms and therapeutic implications. Nat. Rev. Endocrinol. *11*, 592–605. https://doi.org/10.1038/nrendo.2015.122.

41. Ernst, M.C., and Sinal, C.J. (2010). Chemerin: at the crossroads of inflammation and obesity. Trends Endocrinol. Metab. *21*, 660–667. https://doi.org/10.1016/j.tem.2010.08.001.

42. Abudureyimu, S., Abulaiti, P., Li, H., Xing, Z., Liu, S., Li, W., and Gao, Y. (2021). Roles of endothelial lipase gene related single nucleotide polymorphisms in patients with coronary artery disease. Gene *788*, 145669. https://doi.org/10.1016/j.gene.2021.145669.

CellPress
OPEN ACCESS

43. Meissner, F., Scheltema, R.A., Mollenkopf, H.-J., and Mann, M. (2013). Direct Proteomic Quantification of the Secretome of Activated Immune Cells. Science *340*, 475–478. https://doi.org/10.1126/science.1232578.

44. Uhlen, M., Karlsson, M.J., Hober, A., Svensson, A.S., Scheffel, J., Kotol, D., Zhong, W., Tebani, A., Strandberg, L., Edfors, F., et al. (2019). The human secretome. Sci. Signal. *12*, eaaz0274. https://doi.org/10.1126/scisignal.aaz0274.

45. Rath, S., Sharma, R., Gupta, R., Ast, T., Chan, C., Durham, T.J., Goodman, R.P., Grabarek, Z., Haas, M.E., Hung, W.H.W., et al. (2021). MitoCarta3.0: an updated mitochondrial proteome now with sub-organelle localization and pathway annotations. Nucleic Acids Res. *49*, D1541–D1547. https://doi.org/10.1093/nar/gkaa1011.

46. Vu, L.T., Ahmed, F., Zhu, H., Iu, D.S.H., Fogarty, E.A., Kwak, Y., Chen, W., Franconi, C.J., Munn, P.R., Tate, A.E., et al. (2024). Single-cell transcriptomics of the immune system in ME/CFS at baseline and following symptom provocation. Cell Rep. Med. *5*, 101373. https://doi.org/10.1016/j.xcrm.2023.101373.

47. Nunes, M., Vlok, M., Proal, A., Kell, D.B., and Pretorius, E. (2024). Data-independent LC-MS/MS analysis of ME/CFS plasma reveals a dysregulated coagulation system, endothelial dysfunction, downregulation of complement machinery. Cardiovasc. Diabetol. *23*, 254. https://doi.org/10.1186/s12933-024-02315-x.

48. Giloteaux, L., Li, J., Hornig, M., Lipkin, W.I., Ruppert, D., and Hanson, M.R. (2023). Proteomics and cytokine analyses distinguish myalgic encephalomyelitis/chronic fatigue syndrome cases from controls. J. Transl. Med. *21*, 322. https://doi.org/10.1186/s12967-023-04179-3.

49. Mandarano, A.H., Maya, J., Giloteaux, L., Peterson, D.L., Maynard, M., Gottschalk, C.G., and Hanson, M.R. (2020). Myalgic encephalomyelitis/chronic fatigue syndrome patients exhibit altered T cell metabolism and cytokine associations. J. Clin. Investig. *130*, 1491–1505. https://doi.org/10.1172/jci132185.

50. Fernandez-Guerra, P., Gonzalez-Ebsen, A.C., Boonen, S.E., Courraud, J., Gregersen, N., Mehlsen, J., Palmfeldt, J., Olsen, R.K.J., and Brinth, L.S. (2021). Bioenergetic and Proteomic Profiling of Immune Cells in Myalgic Encephalomyelitis/Chronic Fatigue Syndrome Patients: An Exploratory Study. Biomolecules *11*, 961. https://doi.org/10.3390/biom11070961.

51. Che, X., Ranjan, A., Guo, C., Zhang, K., Goldsmith, R., Levine, S., Moneghetti, K.J., Zhai, Y., Ge, L., Mishra, N., et al. (2025). Heightened innate immunity may trigger chronic inflammation, fatigue and post-exertional malaise in ME/CFS. NPJ Metab. Health Dis. *3*, 34. https://doi.org/10.1038/s44324-025-00079-w.

52. Campen, C.L.M.C.v., and Visser, F.C. (2018). Blood Volume Status in Patients with Chronic Fatigue Syndrome: Relation to Complaints. Int. J. Clin. Med. *09*, 809–819. https://doi.org/10.4236/ijcm.2018.911067.

53. Jammes, Y., Adjriou, N., Kipson, N., Criado, C., Charpin, C., Rebaudet, S., Stavris, C., Guieu, R., Fenouillet, E., and Retornaz, F. (2020). Altered muscle membrane potential and redox status differentiates two subgroups of patients with chronic fatigue syndrome. J. Transl. Med. *18*, 173. https://doi.org/10.1186/s12967-020-02341-9.

54. Pietrangelo, T., Fulle, S., Coscia, F., Gigliotti, P.V., and Fanò-Illic, G. (2018). Old muscle in young body: an aphorism describing the Chronic Fatigue Syndrome. Eur. J. Transl. Myol. *28*, 7688. https://doi.org/10.4081/ejtm.2018.7688.

55. Petter, E., Scheibenbogen, C., Linz, P., Stehning, C., Wirth, K., Kuehne, T., and Kelm, M. (2022). Muscle sodium content in patients with Myalgic Encephalomyelitis/Chronic Fatigue Syndrome. J. Transl. Med. *20*, 580. https://doi.org/10.1186/s12967-022-03616-z.

56. Montoya, J.G., Holmes, T.H., Anderson, J.N., Maecker, H.T., Rosenberg-Hasson, Y., Valencia, I.J., Chu, L., Younger, J.W., Tato, C.M., and Davis, M.M. (2017). Cytokine signature associated with disease severity in chronic fatigue syndrome patients. Proc. Natl. Acad. Sci. USA *114*, E7150–E7158. https://doi.org/10.1073/pnas.1710519114.

57. Landi, A., Broadhurst, D., Vernon, S.D., Tyrrell, D.L.J., and Houghton, M. (2016). Reductions in circulating levels of IL-16, IL-7 and VEGF-A in myalgic encephalomyelitis/chronic fatigue syndrome. Cytokine *78*, 27–36. https://doi.org/10.1016/j.cyto.2015.11.018.

58. Ishimura, S., Furuhashi, M., Watanabe, Y., Hoshina, K., Fuseya, T., Mita, T., Okazaki, Y., Koyama, M., Tanaka, M., Akasaka, H., et al. (2013). Circulating Levels of Fatty Acid-Binding Protein Family and Metabolic Phenotype in the General Population. PLoS One *8*, e81318. https://doi.org/10.1371/journal.pone.0081318.

59. Song, Y., Li, J., and Wu, Y. (2024). Evolving understanding of autoimmune mechanisms and new therapeutic strategies of autoimmune disorders. Signal Transduct. Targeted Ther. *9*, 263. https://doi.org/10.1038/s41392-024-01952-8.

60. Iwasaki, A., and Putrino, D. (2023). Why we need a deeper understanding of the pathophysiology of long COVID. Lancet Infect. Dis. *23*, 393–395. https://doi.org/10.1016/s1473-3099(23)00053-1.

61. Iu, D.S., Maya, J., Vu, L.T., Fogarty, E.A., McNairn, A.J., Ahmed, F., Franconi, C.J., Munn, P.R., Grenier, J.K., Hanson, M.R., and Grimson, A. (2024). Transcriptional reprogramming primes CD8+ T cells toward exhaustion in Myalgic encephalomyelitis/chronic fatigue syndrome. Proc. Natl. Acad. Sci. USA *121*, e2415119121. https://doi.org/10.1073/pnas.2415119121.

62. Cassatella, M.A., Östberg, N.K., Tamassia, N., and Soehnlein, O. (2019). Biological Roles of Neutrophil-Derived Granule Proteins and Cytokines. Trends Immunol. *40*, 648–664. https://doi.org/10.1016/j.it.2019.05.003.

63. Koenderman, L., Tesselaar, K., and Vrisekoop, N. (2022). Human neutrophil kinetics: a call to revisit old evidence. Trends Immunol. *43*, 868–876. https://doi.org/10.1016/j.it.2022.09.008.

64. Ballanti, E., Perricone, C., Greco, E., Ballanti, M., Di Muzio, G., Chimenti, M.S., and Perricone, R. (2013). Complement and autoimmunity. Immunol. Res. *56*, 477–491. https://doi.org/10.1007/s12026-013-8422-y.

65. van Eeden, C., Mohazab, N., Redmond, D., Yacyshyn, E., Clifford, A., Russell, A.S., Osman, M.S., and Cohen Tervaert, J.W. (2023). Myalgic encephalomyelitis/chronic fatigue syndrome (ME/CFS) and fibromyalgia: PR3-versus MPO-ANCA-associated vasculitis, an exploratory cross-sectional study. Lancet Reg. Health. Am. *20*, 100460. https://doi.org/10.1016/j.lana.2023.100460.

66. Scheibenbogen, C., Loebel, M., Freitag, H., Krueger, A., Bauer, S., Antelmann, M., Doehner, W., Scherbakov, N., Heidecke, H., Reinke, P., et al. (2018). Immunoadsorption to remove ß2 adrenergic receptor antibodies in Chronic Fatigue Syndrome CFS/ME. PLoS One *13*, e0193672. https://doi.org/10.1371/journal.pone.0193672.

67. Nalbant, D., Youn, H., Nalbant, S.I., Sharma, S., Cobos, E., Beale, E.G., Du, Y., and Williams, S.C. (2005). FAM20: an evolutionarily conserved family of secreted proteins expressed in hematopoietic cells. BMC Genom. *6*, 11. https://doi.org/10.1186/1471-2164-6-11.

68. Sandvik, M.K., Sørland, K., Leirgul, E., Rekeland, I.G., Stavland, C.S., Mella, O., and Fluge, Ø. (2023). Endothelial dysfunction in ME/CFS patients. PLoS One *18*, e0280942. https://doi.org/10.1371/journal.pone.0280942.

69. Sørland, K., Sandvik, M.K., Rekeland, I.G., Ribu, L., Småstuen, M.C., Mella, O., and Fluge, Ø. (2021). Reduced Endothelial Function in Myalgic Encephalomyelitis/Chronic Fatigue Syndrome–Results From Open-Label Cyclophosphamide Intervention Study. Front. Med. *8*, 642710. https://doi.org/10.3389/fmed.2021.642710.

70. Scherbakov, N., Szklarski, M., Hartwig, J., Sotzny, F., Lorenz, S., Meyer, A., Grabowski, P., Doehner, W., and Scheibenbogen, C. (2020). Peripheral endothelial dysfunction in myalgic encephalomyelitis/chronic fatigue syndrome. ESC Heart Fail. *7*, 1064–1071. https://doi.org/10.1002/ehf2.12633.

71. Feletou, M. (2011). In: The Endothelium: Part 1: Multiple Functions of the Endothelial Cells-Focus on Endothelium-Derived Vasoactive Mediators. In The Endothelium: Part 1: Multiple Functions of the Endothelial Cells-Focus on Endothelium-Derived Vasoactive Mediators. https://doi.org/10.4199/C00031ED1V01Y201105ISP019.

72. Wilson, C., Lee, M.D., Buckley, C., Zhang, X., and McCarron, J.G. (2023). Mitochondrial ATP Production is Required for Endothelial Cell Control of Vascular Tone. Function (Oxf) *4*, zqac063. https://doi.org/10.1093/function/zqac063.

73. McMahon, T.J. (2019). Red Blood Cell Deformability, Vasoactive Mediators, and Adhesion. Front. Physiol. *10*, 1417. https://doi.org/10.3389/fphys.2019.01417.

74. Aschman, T., Wyler, E., Baum, O., Hentschel, A., Legler, F., Preusse, C., Meyer-Arndt, L., Büttnerova, I., Förster, A., Cengiz, D., et al. (2023). Post-COVID Syndrome Is Associated with Capillary Alterations, Macrophage Infiltration and Distinct Transcriptomic Signatures in Skeletal Muscles (Cold Spring Harbor Laboratory).

75. Iosef, D.C., Knauer, M.J., Nicholson, M., Nynatten, L.R.V., Cepinskas, D.G., Draghici, S., and Han, V.K.M.; Fraser (2023). Plasma Proteome of Long-covid Patients Indicates Hypoxia-mediated Vasculo-proliferative Disease With Impact on Brain and Heart Function (Research Square Platform LLC).

76. Hanson, A.L., Mulè, M.P., Ruffieux, H., Mescia, F., Bergamaschi, L., Pelly, V.S., Turner, L., Kotagiri, P., , et al.; Cambridge Institute of Therapeutic Immunology and Infectious Disease–National Institute for Health Research CITIID–NIHR COVID BioResource Collaboration; and Göttgens, B. (2024). Iron dysregulation and inflammatory stress erythropoiesis associates with long-term outcome of COVID-19. Nat. Immunol. *25*, 471–482. https://doi.org/10.1038/s41590-024-01754-8.

77. Wang, P.-Y., Ma, J., Kim, Y.-C., Son, A.Y., Syed, A.M., Liu, C., Mori, M.P., Huffstutler, R.D., Stolinski, J.L., Talagala, S.L., et al. (2023). WASF3 disrupts mitochondrial respiration and may mediate exercise intolerance in myalgic encephalomyelitis/chronic fatigue syndrome. Proc. Natl. Acad. Sci. USA *120*, e2302738120. https://doi.org/10.1073/pnas.2302738120.

78. Melvin, A., Lacerda, E., Dockrell, H.M., O'Rahilly, S., and Nacul, L. (2019). Circulating levels of GDF15 in patients with myalgic encephalomyelitis/chronic fatigue syndrome. J. Transl. Med. *17*, 409. https://doi.org/10.1186/s12967-019-02153-6.

79. Chow, L.S., Gerszten, R.E., Taylor, J.M., Pedersen, B.K., Van Praag, H., Trappe, S., Febbraio, M.A., Galis, Z.S., Gao, Y., Haus, J.M., et al. (2022). Exerkines in health, resilience and disease. Nat. Rev. Endocrinol. *18*, 273–289. https://doi.org/10.1038/s41574-022-00641-2.

80. Chen, P.-S., Chiu, W.-T., Hsu, P.-L., Lin, S.-C., Peng, I.C., Wang, C.-Y., and Tsai, S.-J. (2020). Pathophysiological implications of hypoxia in human diseases. J. Biomed. Sci. *27*, 63. https://doi.org/10.1186/s12929-020-00658-7.

81. Joseph, P., Arevalo, C., Oliveira, R.K.F., Faria-Urbina, M., Felsenstein, D., Oaklander, A.L., and Systrom, D.M. (2021). Insights From Invasive Cardiopulmonary Exercise Testing of Patients With Myalgic Encephalomyelitis/Chronic Fatigue Syndrome. Chest *160*, 642–651. https://doi.org/10.1016/j.chest.2021.01.082.

82. Singh, I., Joseph, P., Heerdt, P.M., Cullinan, M., Lutchmansingh, D.D., Gulati, M., Possick, J.D., Systrom, D.M., and Waxman, A.B. (2022). Persistent Exertional Intolerance After COVID-19: Insights From Invasive Cardiopulmonary Exercise Testing. Chest *161*, 54–63. https://doi.org/10.1016/j.chest.2021.08.010.

83. Grassi, B., Marzorati, M., Lanfranconi, F., Ferri, A., Longaretti, M., Stucchi, A., Vago, P., Marconi, C., and Morandi, L. (2007). Impaired oxygen extraction in metabolic myopathies: Detection and quantification by near-infrared spectroscopy. Muscle Nerve *35*, 510–520. https://doi.org/10.1002/mus.20708.

84. Zhang, S., Jahanbani, F., Chander, V., Kjellberg, M., Liu, M., Glass, K.A., Iu, D.S., Ahmed, F., Li, H., Maynard, R.D., et al. (2025). Dissecting the Genetic Complexity of Myalgic Encephalomyelitis/chronic Fatigue Syndrome via Deep Learning-Powered Genome Analysis (Cold Spring Harbor Laboratory).

85. Huang, K., Lidbury, B.A., Thomas, N., Gooley, P.R., and Armstrong, C.W. (2025). Machine learning and multi-omics in precision medicine for ME/CFS. J. Transl. Med. *23*, 68. https://doi.org/10.1186/s12967-024-05915-z.

86. Chapola, H., de Bastiani, M.A., Duarte, M.M., Freitas, M.B., Schuster, J.S., de Vargas, D.M., and Klamt, F. (2023). A comparative study of COVID-19 transcriptional signatures between clinical samples and preclinical cell models in the search for disease master regulators and drug repositioning candidates. Virus Res. *326*, 199053. https://doi.org/10.1016/j.virusres.2023.199053.

87. Fluge, Ø., Mella, O., Bruland, O., Risa, K., Dyrstad, S.E., Alme, K., Rekeland, I.G., Sapkota, D., Røsland, G.V., Fosså, A., et al. (2016). Metabolic profiling indicates impaired pyruvate dehydrogenase function in myalgic encephalopathy/chronic fatigue syndrome. JCI Insight *1*, e89376. https://doi.org/10.1172/jci.insight.89376.

88. Candia, J., Fantoni, G., Delgado-Peraza, F., Shehadeh, N., Tanaka, T., Moaddel, R., Walker, K.A., and Ferrucci, L. (2024). Variability of 7K and 11K SomaScan Plasma Proteomics Assays. J. Proteome Res. *23*, 5531–5539. https://doi.org/10.1021/acs.jproteome.4c00667.

89. Katz, D.H., Robbins, J.M., Deng, S., Tahir, U.A., Bick, A.G., Pampana, A., Yu, Z., Ngo, D., Benson, M.D., Chen, Z.Z., et al. (2022). Proteomic profiling platforms head to head: Leveraging genetics and clinical traits to compare aptamer- and antibody-based methods. Sci. Adv. *8*, eabm5164. https://doi.org/10.1126/sciadv.abm5164.

90. Perez-Riverol, Y., Bandla, C., Wang, S., Vizcaíno, J.A., Kundu, D.J., Kamatchinathan, S., Bai, J., Hewapathirana, S., John, N.S., Prakash, A., and Walzer, M. (2025). The PRIDE database at 20 years: 2025 update. Nucleic Acids Res. *53*, D543–D553. https://doi.org/10.1093/nar/gkae1011.

91. Thul, P.J., and Lindskog, C. (2018). The human protein atlas: A spatial map of the human proteome. Protein Sci. *27*, 233–244. https://doi.org/10.1002/pro.3307.

92. Ritchie, M.E., Phipson, B., Wu, D., Hu, Y., Law, C.W., Shi, W., and Smyth, G.K. (2015). limma powers differential expression analyses for RNA-sequencing and microarray studies. Nucleic Acids Res. *43*, e47. https://doi.org/10.1093/nar/gkv007.

93. Kim, S. (2015). ppcor: An R Package for a Fast Calculation to Semi-partial Correlation Coefficients. Commun. Stat. Appl. Methods *22*, 665–674. https://doi.org/10.5351/csam.2015.22.6.665.

94. The Gene Ontology Consortium (2019). The Gene Ontology Resource: 20 years and still GOing strong. Nucleic Acids Res. *47*, D330–D338. https://doi.org/10.1093/nar/gky1055.

95. Zhang, Y., Liu, T., Wang, J., Zou, B., Li, L., Yao, L., Chen, K., Ning, L., Wu, B., Zhao, X., and Wang, D. (2021). Cellinker: a platform of ligand–receptor interactions for intercellular communication analysis. Bioinformatics *37*, btab036–2032. https://doi.org/10.1093/bioinformatics/btab036.

96. R-Core-Team (2019). R: A Language and Environment for Statistical Computing (R Foundation for Statistical Computing).

97. RStudio-Team (2015). RStudio: Integrated Development for R (RStudio, Inc.).

STAR★METHODS

KEY RESOURCES TABLE

REAGENT or RESOURCE	SOURCE	IDENTIFIER
Critical commerical assays		
Custom Luminex Human Discovery assay	R&D Systems	Cat# LXSAHM; RRID:AB_2924693
Quantikine Ready-To-Use ELISA kit (FGF-21)	R&D Systems	Cat# DF2100; RRID:AB_2783729
Quantikine Ready-To-Use ELISA kit (GDF-15)	R&D Systems	Cat# DGD150; RRID:AB_2877710
Quantikine Ready-To-Use ELISA kit (C-peptide)	R&D Systems	Cat# DICP00
SomaScan Discovery, 7 K	SomaLogic Inc	v. 4.1
Biological samples		
Human serum samples	ME/CFS Biobank, Haukeland University Hospital, Norway	RituxME: NCT02229942 CycloME: NCT02444091
Deposited data		
Proteomics dataset	Deposited in the PRIDE[90] repository (PRIDE: PAD000026)	N/A
Metabolomics data	Hoel et al.[17]; DOI: https://doi.org/10.1172/jci.insight.149217	N/A
Software and algorithms		
GraphPad Prism 9	GraphPad Software	N/A
R (4.5.1)	https://www.r-project.org	N/A
Rstudio (2025.09.1 + 401)	Posit Software	N/A
R package: Vegan (2.7.0)	DOI: https://doi.org/10.32614/CRAN.package.vegan	N/A
R package: limma (3.64.3)	DOI: https://doi.org/10.18129/B9.bioc.limma	N/A
R package: variancePartition (1.38.1)	DOI: https://doi.org/10.18129/B9.bioc.variancePartition	N/A
R package: ppcor (1.1)	DOI: https://doi.org/10.32614/CRAN.package.ppcor	N/A
R package: clusterProfiler (4.16.0)	DOI: https://doi.org/10.18129/B9.bioc.clusterProfiler	N/A
R package: ComplexHeatmap (2.24.1)	DOI: https://doi.org/10.18129/B9.bioc.ComplexHeatmap	N/A
R package: ggplot2 (4.0.0)	DOI: https://doi.org/10.32614/CRAN.package.ggplot2	N/A
R package: tidygraph (1.3.1)	DOI: https://doi.org/10.32614/CRAN.package.tidygraph	N/A
R package: igraph (2.2.0)	DOI: https://doi.org/10.32614/CRAN.package.igraph	N/A
R package: ggpubr (0.6.2)	DOI: https://doi.org/10.32614/CRAN.package.ggpubr	N/A
Cellinker	DOI: https://doi.org/10.1093/bioinformatics/btab036	N/A
BioRender	BioRender	N/A
Microsoft Office Excel	Microsoft	N/A
Other		
Human protein atlas (HPA)	https://www.proteinatlas.org/	N/A

EXPERIMENTAL MODEL AND STUDY PARTICIPANT DETAILS

Human participants

This study analyzed serum samples from participants enrolled in the RituxME[31] and CycloME[32] clinical trials, as well as healthy control volunteers. Participants were allocated to ME/CFS or healthy control (HC) groups based on clinical status (ME/CFS diagnosis using Canadian criteria[6] vs. HC). Healthy controls were age- and sex-matched. Within the eligible pool of samples, individuals were randomly selected to minimize selection bias. Serum samples were stored at −80°C.

In total, 54 ME/CFS and 29 HC participants were analyzed on the aptamer-platform. Age, sex, BMI, and fasting status were recorded for all participants. Sex was included as a covariate in statistical models, and sex-stratified analyses were performed for selected targets where feasible; however, the study was not powered to systematically evaluate sex-specific effects across all proteomic features. Eleven ME/CFS subjects who performed overnight fasting before the sampling were included to facilitate evaluation of the impact of fasting as a covariate factor. After preprocessing and outlier removal, 50 ME/CFS and 29 HC samples remained for analysis (see below, and DocumentS1: Methods S1A, Table S1). For covariate-adjusted univariate testing, 50 ME/CFS and 24 HC samples were retained; for metabotype[17] analyses, 40 ME/CFS subjects, all but one non-fasting, and 24 non-fasting HC subjects were included.

In the validation measurements (Luminex and ELISA), samples were randomly picked from the ME/CFS biobank, including a total of 212 ME/CFS patients and 66 healthy individuals.

Ethics statement

All studies were conducted in accordance with the Declaration of Helsinki and approved by the Regional Committee for Medical and Health Research Ethics, Western Norway (REK Vest; Tromsø, Norway; no. 2010/1318-4, no. 2014/365, and no. 2014/1672). All participants provided written informed consent prior to sample collection and analysis. Data were de-identified prior to proteomic and statistical analysis.

METHOD DETAILS

SomaLogic proteomic profiling

Serum proteins were quantified using the SomaScan v4.1 platform (SomaLogic Inc., Boulder, CO), measuring 7326 aptamer-based targets. Data was received as normalized relative fluorescence units (RFU). SomaLogic normalization and hybridization controls were applied by the vendor, followed by hybridization signal calibration and median signal normalization across runs. The affinity proteomics data have been deposited to the PRIDE[90] repository (PRIDE: PAD000026).

Four samples were identified as outliers using PCA on Mahalanobis distances computed from $\log_{10}$-transformed intensities (see DocumentS1: Methods S1A, Figure S1), with statistical inference based on a chi-square test ($p < 0.1$). High-leverage aptamers were identified using Z-scores (±1.8, corresponding to the 2.5th and 97.5th percentiles) on raw intensities and assigned N/A, after which missing values were imputed using the variable's minimum and maximum post-outlier removal. Further downstream analyses were conducted using log2-transformed raw intensities, as required for linear-model frameworks such as the R packages, *limma* and *variancePartition*.

Alignment of aptamer pairs

To assess intra-assay consistency, we compared directional concordance and signal correlation among multiple aptamers targeting the same protein (n = 786 proteins, 1,683 aptamers). Directional consensus was defined as $\geq$ 80% of aptamers showing the same direction of change (ME/CFS vs. HC). Pearson correlations were categorized as weak ($|r| < 0.3$), moderate (0.3–0.5), strong (0.5–0.8), or very strong ($|r| > 0.8$). Discordant aptamers were resolved by a predefined decision tree prioritizing significance ($p < 0.05$) and directionality (see DocumentS1: Methods S1B, Table S2).

Subcellular and immune annotation

Proteins were annotated according to subcellular localization (intracellular, membrane, secreted, membrane-secreted) and immune-cell association using curated panels from HPA.[44,91] Directional changes per class were summarized at the protein level (DocumentS1: Data S1A, Table S3).

CycloME responder analysis

Within the CycloME trial subset, proteomic profiles of responders (n = 21) and non-responders (n = 14) were compared using *limma* with adjustment for age, sex, BMI, and fasting status (SuppData3: 8_CycloME_vs._RvsNR). The PCA, Volcano plot, and top up- and down-regulated proteins are shown in DocumentS1: Data S1B, Figure S2, Tables S4 and S5.

Activity and clinical correlates

Associations between protein abundance and physical activity were examined using both SF-36 Physical Functioning (SF-36PF) scores and mean daily step counts derived from accelerometer recordings.[31,32] Pearson correlations were computed within aptamer communities ($|r| > 0.3$, $p < 0.05$).

Luminex analysis

Seventy-seven target proteins (Data S6) were quantified using the Luminex Human Discovery Assay (Cat. #LXSAHM; R&D Systems), following the manufacturer's instructions.

Five custom assays were designed to cover the analytical targets, and two 96-well plates for each assay were used. The majority of the samples were analyzed in single reaction wells; however, some samples were analyzed as duplicates or included on multiple

plates for inter-and intra-plate quality control. Each plate included fresh target standard dilutions in duplicates. Plates with identical assays were run on the same day. Measurements were performed using the Luminex 200 instrument (Luminex Corp.) and the accompanying analysis software to generate the standard curves and sample concentrations. Measurements that were out the range of the standard curve were excluded.

ELISA

ELISA was performed using Quantikine Ready-to-Use ELISA kits (R&D Systems) for FGF-21 (Cat#: DF2100), C-peptide (Cat#: DICP00), and GDF-15 (Cat#: DG0150). The serum samples were diluted and measured in duplicates, according to the manufacturer's recommendations. Measurements were performed using the Spark microplate reader (Tecan Trading AG, Switzerland). Standard curve generation was conducted in Excel or GraphPad Prism, with further data analysis done in GraphPad Prism and R programming language.

Interstudy comparison

Findings were compared with two published SomaLogic ME/CFS datasets, Germain et al., 2021,[29] and Walitt et al., 2024.[30] Both datasets were processed and analyzed using comparable quality-control and *limma* pipelines, although an identical linear regression model was not possible due to missing covariates for the Walitt and Germain datasets. Variable outliers were assessed and handled using identical protocol. Shared up- and down-regulated proteins were identified by gene symbol overlap (DocumentS1: Data S1I; Data S7)).

QUANTIFICATION AND STATISTICAL ANALYSIS

For SomaScan data, a principal component analysis (PCA) with the first two principal components used for sample projections, and clinical variables correlated with sample coordinates via the *envfit* function (Vegan v2.7.0). Univariate analysis of SomaScan and Luminex data was performed using linear regression (adjusting for age, sex, BMI, and fasting baseline) with *limma*.[92] Explained variance was further assessed on variables with statistical significance ($p < 0.05$) using the function *fitExtractVarPartModel* in the *VariancePartition* library (GitHub - DiseaseNeuroGenomics/variancePartition). Semi-partial Pearson correlation coefficients were calculated for relevant subsets, adjusted for the same covariates, were calculated using *ppcor*,[93] filtered by a standard cutoff ($p < 0.05$, absolute $r > 0.3$) visualized in a clinical-protein network, where protein communities were defined by shared clinical interactions. Functional enrichment was conducted using Gene Ontology[94] with *ClusterProfiler* (GitHub - YuLab-SMU/clusterProfiler), while ligand-receptor interactions were annotated via *CellLinker*,[95] and functional/spatial annotations were retrieved from the Human Protein Atlas.[44,91] All visualizations were generated using *ggplot2*, *ComplexHeatmap, tidygraph*, *igraph*, *ggnet*, and *ggpubr*. All statistical analyses for aptamer-based proteomics and Luminex were performed using the R programming language[96] within the RStudio environment.[97] BioRender was used for composition of figures. For cross-platform evaluation (SomaScan vs. Luminex), Pearson correlation coefficients were calculated using base R tools, and concordance patterns were compared for shared targets. Significance thresholds were two-sided $p < 0.05$ unless otherwise stated. Comparisons of ELISA data were performed between groups (Welch's *t* test with FDR correction, $q < 0.05$), and stratified by sex and metabotype (one-way ANOVA, $p < 0.05$; followed by post-hoc Welch's *t* test with FDR correction, $q < 0.05$).

ADDITIONAL RESOURCES

The clinical trials providing samples for aptamer-based proteomics were RituxME (ClinicalTrials.gov: NCT02229942) and CycloME (ClinicalTrials.gov: NCT02444091).

The supplemental information and Excel files (Data S1, S2, S3, S4, S5, S6, and S7) provide full SomaScan and Luminex datasets, metadata, and statistical outputs.

Cell Reports Medicine, Volume 7

Supplemental information

Charting the circulating proteome in ME/CFS using cross-system profiling to uncover mechanistic insights

August Hoel, Fredrik Hoel, Sissel Elisabeth Dyrstad, Henrique Chapola, Ingrid Gurvin Rekeland, Kristin Risa, Kine Alme, Kari Sørland, Karl Albert Brokstad, Hans-Peter Marti, Olav Mella, Øystein Fluge, and Karl Johan Tronstad

Document S1: Supplementary information

Content:

DocumentS1.pdf (this file)

Methods S1A:	Data and analytical steps	Table S1, Figure S1
Methods S1B:	Alignment between aptamer pairs	Table S2
Data S1A:	Protein-level summary statistics	Table S3
Data S1B:	CycloME responders vs. non-responders	Tables S4- S5, Figure S2
Data S1C:	Correlation with mean number of steps	Table S6, Figure S3
Data S1D:	Immune cell profiles	Table S7
Data S1E:	Blood cell counts	Table S8
Data S1F:	Ligand - receptor alignment	Figure S4
Data S1G:	Luminex cohort	Table S9
Data S1H:	Cross-platform concordance results (SomaScan vs Luminex)	
Data S1I:	Interstudy comparison	Tables S10-S13, Figures S5-S6

Methods S1A: Data and analytical steps

Cohort size:

Table S1: Summary of subjects included in key analytical steps
Related to cohort described in STAR Methods.

Step	ME/CFS	HC
Initial population	54	29
After preprocessing (Outlier removal)	50	29
For univariate analysis using limma (adjusting for BMI, age, sex and fasting)	50	24
Analysis of metabotypes	40	24

Univariate analysis was performed using a linear regression model including BMI, age, sex, and overnight fasting as covariates, as covariate adjustment was required; consequently, five HC subjects lacking these data were omitted (Table S1).

Removal of outliers:

SomaLogic provides normalized data (RFU) and recommends a log10 transformation, as individual outliers can disproportionately influence analyses. The statistical impact of such outliers is well-documented using this platform, notably Germain et. al. 2021 (PMID: 33572894) addressed this as a major challenge for downstream analysis in their aptamer-based ME/CFS study.

Four samples were identified as outliers using PCA on Mahalanobis distances computed from $\log_{10}$-transformed intensities (Figure S1, STAR Methods)

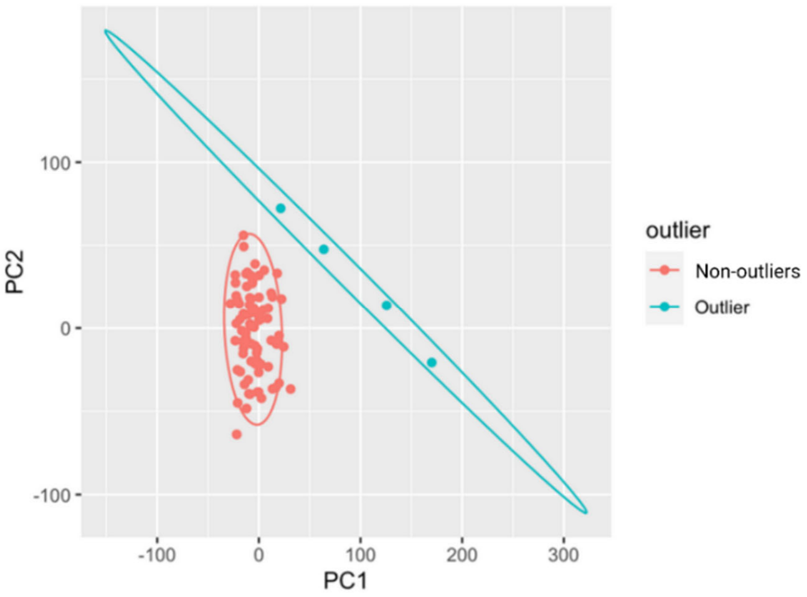

Figure S1. PCA on Mahalanobis distances detecting four outliers.
Related to Somalogic data processing as described in STAR Methods.

Methods S1B: Alignment between aptamer pairs

Aptamer pair alignment analyses

We performed directional concordance and signal consistency analysis between different aptamers targeting the same protein (n = 786 proteins, 1683 aptamers) (SuppData1: 4_Aptamer_pairs_alignment). Overall, moderate to very strong correlation (|R| > 0.3) was shown for 41.6% of the proteins, while 58.4% showed weak correlations (|R| < 0.3) (Table S2). This is most likely explained by isoform- or domain-specific binding effects (e.g., splice variants), since aptamers often recognize different domains of the same protein, and circulating proteins may exist in multiple isoforms, proteolytic fragments, or post-translationally modified forms. Importantly, despite these variations at the molecular level, almost all the significant aptamers reported concordant directional changes (p<0.05) between ME/CFS and controls. As shown by the low numbers in the "Mixed (<80% same dir)" row in Table S2, few proteins reported conflicting effects, supporting the robustness of the group-level findings.

Table S2: Alignment between aptamer pairs. The table shows the number of proteins targeted by multiple aptamers, stratified by directional consensus at group-level (p<0.05 ME/CFS vs HC, rows), across different consistency categories determined by correlation strength (Pearson, |r|) between aptamer-aptamer pairs (columns). Percentages are relative to the total number of proteins targeted by multiple aptamers (786 proteins,1683 aptamers in total). The "Mixed (<80% same dir)" shows the number of proteins targeted by multiple aptamers, where less than 80% of the aptamers reported the same directional change. Related to Figure 1 and SuppData1: 4_Aptamer_pairs_alignment.

Directional Consensus	***Weak (n, %) |R|<0.3***	***Moderate (n, %) |R|=0.3-0.5***	***Strong (n, %) |R|=0.5-0.8***	***Very Strong (n, %) |R|>0.8***	***Total***
(No p<0.05 hits)	*303 (38.8%)*	*54 (6.9%)*	*54 (6.9%)*	*82 (10.5%)*	***493 (62.7%)***
Down (all sig)	*81 (10.4%)*	*17 (2.2%)*	*25 (3.2%)*	*30 (3.8%)*	***153 (19.5%)***
Mixed (<80% same	*7 (0.9%)*	*3 (0.4%)*	–	–	***10 (1.3%)***
Up (all sig)	*68 (8.7%)*	*22 (2.8%)*	*15 (1.9%)*	*25 (3.2%)*	***130 (16.5%)***
Total	***459 (58.4%)***	***96 (12.2%)***	***94 (12.0%)***	***137 (17.4%)***	***786 (100%)***

Method for counting proteins targeted by multiple aptamers

Multiple aptamers can target the same protein; however, these may exhibit discordant statistical significance and opposite directions of change. When counting significant proteins, duplicates are removed. This process, however, depends on the sorted order of the dataset, which may lead to inconsistent results.

To avoid overestimation of significant proteins, duplicated targets were merged using a consistent rule set based on statistical significance and direction of change (logFC):

1. Discordant significance (n = 2):
   If one aptamer had $p < 0.05$ and the other $p \geq 0.05$, the aptamer with $p < 0.05$ was retained.
2. Opposite direction (n = 2):
   If both aptamers had $p < 0.05$ but opposite directions of change, the aptamer with the lowest p value was retained.
3. Multiple aptamers (n > 2) with concordant direction:
   - If the number of significant aptamers (s) < the number of non-significant aptamers (ns), one non-significant aptamer was retained.
   - If $s > ns$, one significant aptamer (the one with the lowest p) was retained.
4. Multiple aptamers (n > 2) with mixed direction:
   The direction (Δ) with the largest number of significant aptamers was retained:
   $s = \max(s_{+1}, s_{-1})$.
5. Equal number of up- and downregulated significant aptamers (n > 2):
   If $s_{+1} = s_{-1}$, the aptamer with the lowest p value was retained, regardless of direction.

Data S1A: Protein-level summary statistics

Table S3: Protein-level changes across subcellular classes. Summary of protein changes within subcellular classes in ME/CFS relative to healthy controls (HC), aggregated at the protein level rather than the aptamer level. The number of proteins and corresponding percentage in each category are shown. "Down" and "Up" indicate lower or higher protein levels in ME/CFS, respectively. To estimate the total number of significant changes, the counts for $p < 0.05$ and $q < 0.05$ can be summed. Related to Figures 1 and 3.

Category	Intracellular	Secreted	Membrane	Membrane & Secreted	All (Excl. NA)	Non-assigned (NA)	All (Incl. NA)
**Down q**	468 (12.7%)	43 (4.1%)	60 (4.2%)	3 (2.1%)	574 (9.1%)	17 (8.7%)	591 (9.1%)
**Down p**	349 (9.5%)	41 (3.9%)	68 (4.8%)	7 (5.0%)	465 (7.4%)	12 (6.1%)	477 (7.3%)
**Total Down**	817 (22.2%)	84 (8.0%)	128 (9.0%)	10 (7.1%)	1,039 (16.5%)	29 (14.8%)	1,068 (16.5%)
**Up q**	92 (2.5%)	65 (6.2%)	53 (3.7%)	5 (3.6%)	215 (3.4%)	5 (2.6%)	220 (3.4%)
**Up p**	190 (5.2%)	99 (9.4%)	124 (8.7%)	8 (5.7%)	421 (6.7%)	14 (7.1%)	435 (6.7%)
**Total Up**	282 (7.7%)	164 (15.5%)	177 (12.4%)	13 (9.3%)	636 (10.1%)	19 (9.7%)	655 (10.1%)
**No Change**	2,573 (70.1%)	808 (76.5%)	1,124 (78.7%)	117 (83.6%)	4,622 (73.4%)	148 (75.5%)	4,770 (73.5%)
**Total excl. NA**	3,672 (100%)	1,056 (100%)	1,429 (100%)	140 (100%)	6,297 (100%)	–	–
**Total incl. NA**	–	–	–	–	–	196 (100%)	6,493 (100%)

Data S1B: CycloME responders vs. non-responders

A) **B)**

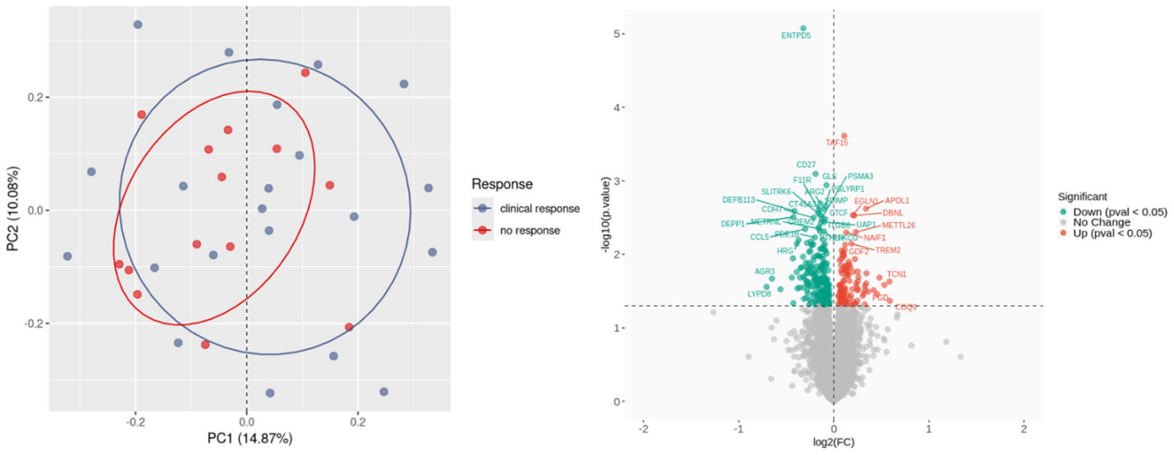

Figure S2. CycloME responders (n=21) and non-responders (n=14). A) PCA, B) Volcano Plot. Related to Figure 1.

Table S4. Univariate Statistics (Limma Regression Model); CycloME responders (n=21) *vs.* non-responders (n=14). Related to Figure 1 and SuppData3: 8_CycloME_vs_RvsNR.

Down(q < 0.05)	Down (p <0.05)	No Change	Up (p < 0.05)	Up (p <0.05)
0	210	7003	113	0

Table S5. Top 25 up- and downregulated proteins, sorted by significance; CycloME responders (n=21) *vs.* non-responders (n=14). Related to Figure 1 SuppData3: 8_CycloME_vs_RvsNR.

Direction	EntrezGene-Symbol	log2FC	P.Value		Direction	EntrezGene-Symbol	log2FC	P.Value
↑Up	TAF15	0.1096	0.00024		↓Down	ENTPD5	-0.3205	0.00001
↑Up	APOL1	0.3381	0.00239		↓Down	CD27	-0.1924	0.00081
↑Up	EGLN1	0.2003	0.00292		↓Down	GLS	-0.0771	0.00115
↑Up	DBNL	0.2139	0.00296		↓Down	ARG2	-0.1409	0.00203
↑Up	METTL26	0.23	0.00496		↓Down	PGLYRP1	-0.0852	0.00221
↑Up	NAIF1	0.1297	0.00502		↓Down	SCIMP	-0.1087	0.00243
↑Up	TREM2	0.1847	0.00715		↓Down	F11R	-0.1583	0.00245
↑Up	GDF2	0.1193	0.00738		↓Down	DEFB113	-0.4139	0.00257
↑Up	ARPC2	0.1135	0.00873		↓Down	SLITRK6	-0.165	0.00279
↑Up	LRTM2	0.0831	0.00887		↓Down	CT45A3	-0.1395	0.00291
↑Up	NDRG4	0.0957	0.00943		↓Down	DEPP1	-0.4238	0.0031
↑Up	LRCH4	0.103	0.00994		↓Down	CDH7	-0.2128	0.00318
↑Up	GK5	0.0908	0.01034		↓Down	PSMA3	-0.1265	0.00324
↑Up	MTHFSD	0.1073	0.01057		↓Down	CTCF	-0.0905	0.00331
↑Up	CDC34	0.085	0.01141		↓Down	UAP1	-0.1028	0.00363
↑Up	MVD	0.2214	0.01157		↓Down	ITGB6	-0.135	0.00365
↑Up	TMEM59L	0.0617	0.012		↓Down	GREM2	-0.1545	0.00422
↑Up	HMGB1	0.1565	0.01254		↓Down	METRNL	-0.1597	0.00447
↑Up	GMPPA	0.1652	0.01383		↓Down	CCL5	-0.3001	0.00453
↑Up	RPS19	0.1305	0.01406		↓Down	PRKCG	-0.1041	0.00486
↑Up	CPD	0.0768	0.01407		↓Down	PTHLH	-0.1436	0.00488
↑Up	SAE1|UBA2	0.144	0.01431		↓Down	PDE1B	-0.1977	0.00588
↑Up	EXTL2	0.1072	0.01486		↓Down	CREBL2	-0.1146	0.00634
↑Up	IGF2R	0.1733	0.01513		↓Down	HRG	-0.3707	0.00638
↑Up	LGALS1	0.1223	0.01596		↓Down	MATN4	-0.2411	0.00667

Data S1C: Correlation with mean number of steps

In our main analysis, we used SF-36 Physical Functioning (SF-36PF) as a proxy for physical activity, reasoning that lower SF-36PF scores would likely reflect reduced activity and thus potential deconditioning. The broad proteomic changes observed in ME/CFS that were unrelated to SF-36PF therefore suggested that these changes were not driven by reduced activity or deconditioning.

To further address this point, we performed an additional analysis using mean daily step count (per 24h), which provides a more direct measure of physical activity. We found that a large fraction of aptamers correlating with SF-36PF also correlated with mean step count, confirming that SF-36PF is a good surrogate for activity level. Importantly, the main proteomic changes distinguishing ME/CFS from controls remained unrelated to either SF-36PF or step count, supporting that these changes are not primarily driven by deconditioning.

We acknowledge that reduced activity can have negative physiological effects; however, our findings suggest that these effects are largely separate from the disease-specific serum proteome alterations observed in ME/CFS.

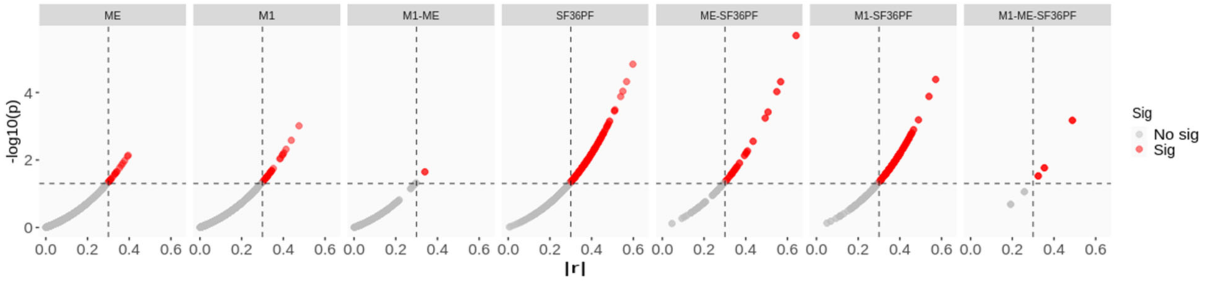

Figure S3. Correlation with mean steps. The figure displays how aptamers correlated to mean steps within each aptamer community. Related to Figure 2 and SuppData2: 5_Correlation_steps.

Table S6. Aptamers correlated to Mean Steps. Threshold for correlation |r| > 3 & p < 0.05. Related to Figure 2 and SuppData2: 5_Correlation_steps.

Community	Correlated	Not Correlated	% Correlated
ME	19	756	2.5%
ME-SF36PF	46	72	39.0%
M1	30	684	4.2%
M1-ME	2	168	1.2%
M1-ME-SF36PF	9	6	60.0%
M1-SF36PF	238	184	56.4%
SF36PF	191	260	42.3%

Data S1D: Immune cell profiles

Table S7. Immune cell profiles. Summary of protein changes within immune cell-associated panels in ME/CFS relative to healthy controls (HC). For each cell type, the number and corresponding percentage of aptamers targeting proteins in each category are shown. “Down” and “Up” indicate lower or higher protein levels in ME/CFS, respectively. To estimate the total number of significant changes, the counts for $p < 0.05$ and $q < 0.05$ can be summed. Related to Figure 4D and SuppData4, 4_ImmuneCells.

**Cell Type**	Down (qval < 0.05)	Down (pval < 0.05)	No Change	Up (pval < 0.05)	Up (qval < 0.05)
**B-cells**	8 (4.3%)	8 (4.3%)	148 (79.6%)	17 (9.1%)	5 (2.7%)
**Dendritic**	20 (4.7%)	22 (5.2%)	337 (79.5%)	24 (5.7%)	21 (5.0%)
**Granulocytes**	51 (8.6%)	46 (7.7%)	458 (76.8%)	27 (4.5%)	14 (2.3%)
**Monocytes**	34 (7.5%)	30 (6.7%)	352 (78.0%)	22 (4.9%)	13 (2.9%)
**NK-cells**	0 (0.0%)	7 (5.7%)	97 (79.5%)	14 (11.5%)	4 (3.3%)
**T-cells**	14 (3.6%)	16 (4.1%)	310 (79.5%)	36 (9.2%)	14 (3.6%)

Data S1E: Blood cell counts

Table S8. Blood cell counts for the ME/CFS group. Summary of blood cell counts in ME/CFS patients participating in the RituxME and CycloME trials. Blood cell data were not available for healthy controls; however, comparison with normal ranges indicated no abnormalities in blood cell counts within the ME/CFS group. Related to Figure 4D and Table S7.

**Measurement**	**Mean**	**SD**	**n**	**Normal**	**Unit**
**Hemoglobin**	14.26	1.16	41	*11.7 - 15.3*	g/dL
**MCV**	88.54	2.78	41	*82 - 98*	fL
**Leucocytes**	6.23	1.35	41	*4 - 10*	10^9/L
**Neutrophile granulocytes**	3.38	1.03	41	*1.5 - 7.3*	10^9/L
**Lymphocytes**	2.20	0.80	41	*1.1 - 3.3*	10^9/L
**Monocytes**	0.45	0.12	41	*0.2 - 0.8*	10^9/L
**Eosinophiles**	0.16	0.11	41	*< 0.4*	10^9/L
**Basophils**	0.05	0.05	41	*< 0.2*	10^9/L
**Thrombocytes**	273.49	64.39	41	*145 - 350*	10^9/L

Data S1F: Ligand – receptor alignment

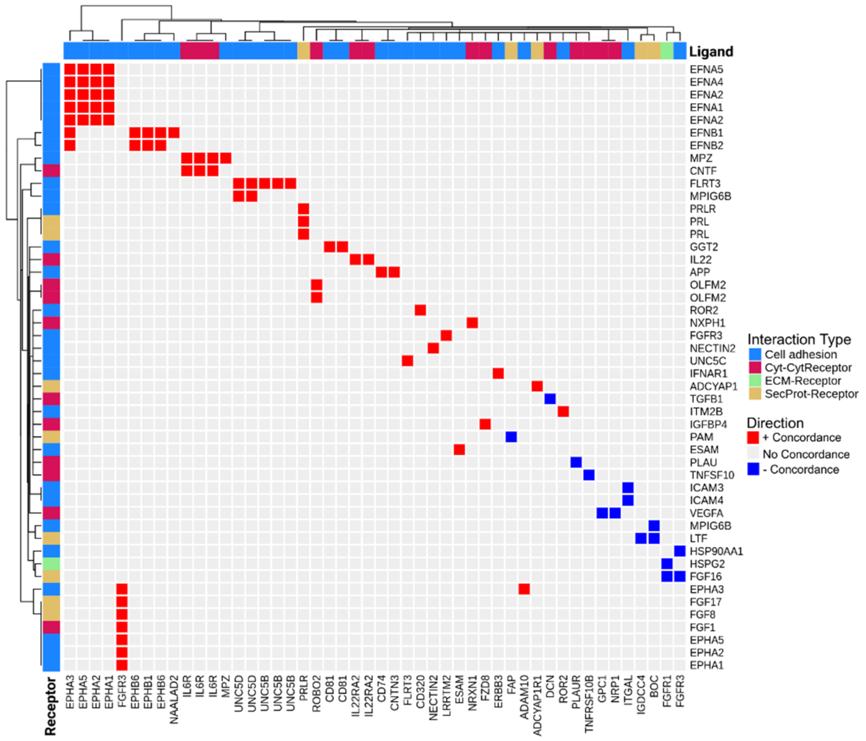

Figure S4: Ligand – receptor alignment. We performed a ligand - receptor alignment analysis to identify pairs of ligands and receptors whose levels change in a coordinated manner. Related to Figure 5.

Data S1G: Luminex cohort

Table S9: Luminex cohort characteristics. The table summarizes group sizes, the percentage (%) of original SomaLogic samples included, and key group characteristics. Related to Figure 6

**Group**	**N (% ovelap *vs.* Somalogic)**	**Age (mean ± SD)**	**Sex (F/M)**	**Female %**	**BMI (kg/m², mean ± SD)**	**Fasting n (%)**
**ME/CFS**	83 (48.2%)	35.0 ± 11.3	64 / 19	77.1%	24.4 ± 4.5	12 (14.5%)
**HC**	29 (20.7%)	36.2 ± 9.6	18 / 11	62.1%	24.0 ± 2.3	0 (0.0%)
**Metabotype 1**	32 (40.62%)	35.2 ± 12.5	24 / 8	75.0%	23.1 ± 4.0	5 (15.6%)
**Metabotype 2**	38 (52.6%)	33.8 ± 11.4	29 / 9	76.3%	25.7 ± 5.0	6 (15.8%)
**Metabotype 3**	13 (53.8%)	37.9 ± 7.8	11 / 2	84.6%	23.8 ± 2.8	1 (7.7%)
**Total**	112	35.3 ± 10.9	82 / 30	73.2%	24.3 ± 4.0	12 10.7%)

Data S1H: Cross-platform concordance results (SomaScan vs Luminex)

Extended description of findings: The examination of directional concordance compared the 54 Luminex proteins with the 68 corresponding aptamers on the SomaScan platform (Fig 6A; SuppData6: 5_Directional_consensus). Here is a summary of the data:

- 38 proteins (70.4 %) / 44 aptamers (64.7 %) showed concordant results (either no significant change or significant change in the same direction).
- 4 proteins (7.4 %) / 10 aptamers (13.5 %) had mixed results because different aptamers targeting the same protein gave differing changes (notably BDNF, COL1A1, OSM, Osteoactivin).
- 12 proteins (22.2 %) / 14 aptamers (18.9 %) were discordant (opposite direction of change between platforms)

Most discordant proteins had low logFC2, except Myoglobin and NAGLU, which showed larger effects on SomaScan than on Luminex.

Signal consistency analysis (Fig. 6B; SuppData6: 6_Signal_correlation), 48 of 68 comparisons (69.1%) showed significant positive correlations ($p < 0.05$), and none showed negative correlations, supporting the robustness of the analysis.

Data S1I: Interstudy comparison

ME/CFS datasets using the aptamer-based platform (SomaLogic):

- Hoel 2025: this study
- Germain 2021: PMID 33572894
- Walitt 2024: PMID 38383456

The compared data are provided in SuppData7.

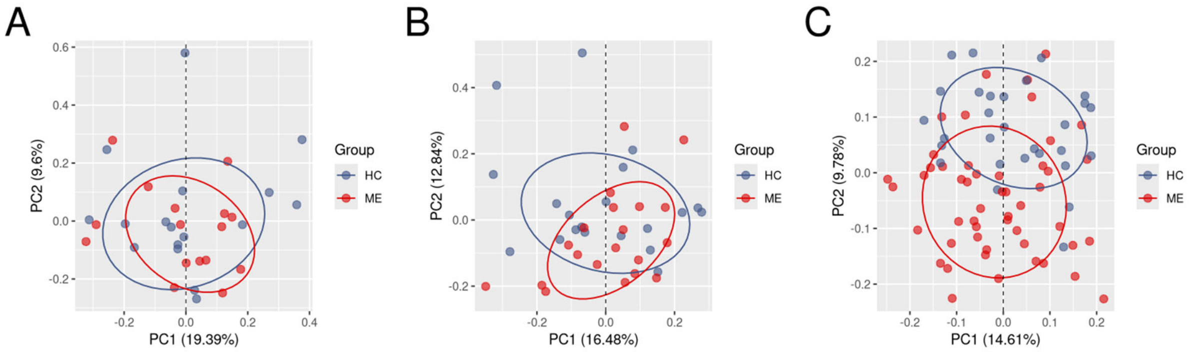

Figure S5. Principal Component Analysis of each Dataset. A) Walitt: PMID 33572894, B) Germain: PMID 38383456, C) Hoel: This study. Related to the Discussion and SuppData7.

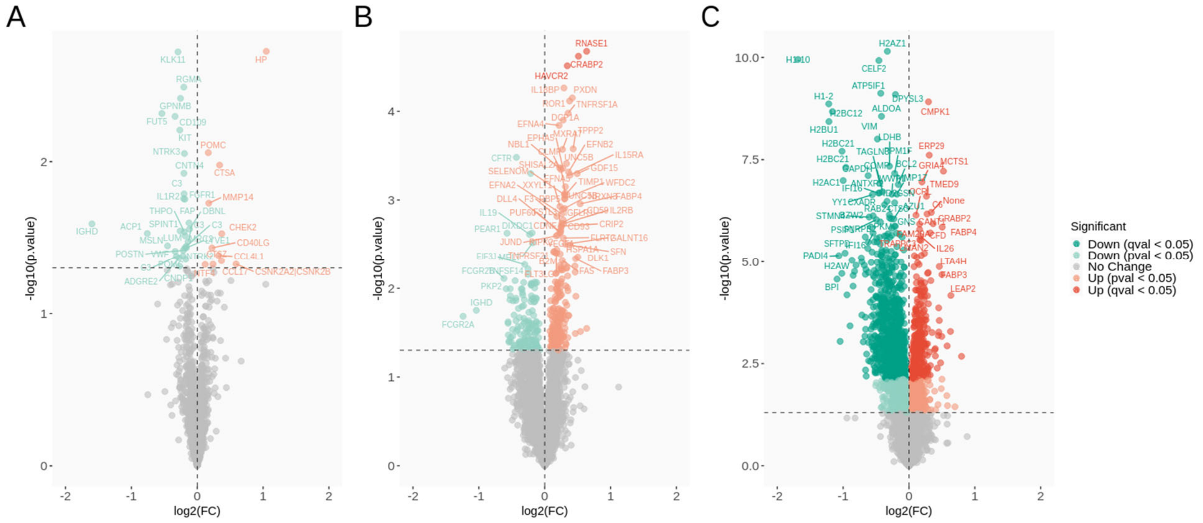

Figure S6. Volcano Plots. A) Walitt: PMID 33572894, B) Germain: PMID 38383456, C) Hoel: This study. Related to the Discussion and SuppData7.

Table S10. Shared Changes. Related to the Discussion and SuppData7.

**Overlaps**	**Up**	**Down**
Germain *vs.* Hoel	*67*	*55*
Germain *vs.* Walitt	*1*	*3*
Hoel *vs.* Walitt	*2*	*5*

Table S11. Changes across compartment | Walitt. Related to the Discussion and SuppData7.

**Compartment**	**Down**	**No change**	**Up**
Intracellular	3 (0.8%)	385 (98.7%)	2 (0.5%)
Membrane	11 (3.7%)	287 (95.7%)	2 (0.7%)
Membrane and secreted isoforms	2 (3.0%)	64 (95.5%)	1 (1.5%)
Secreted	13 (2.9%)	423 (95.9%)	5 (1.1%)
Unannotated	1 (1.4%)	69 (97.2%)	1 (1.4%)

Table S12. Changes across compartments | Germain. Related to the Discussion and SuppData7.

**Compartment**	**Down**	**No change**	**Up**
Intracellular	71 (3.7%)	1757 (92.8%)	66 (3.5%)
Membrane	36 (3.1%)	1062 (90.8%)	71 (6.1%)
Membrane and secreted isoforms	5 (4.0%)	111 (88.1%)	10 (7.9%)
Secreted	15 (1.6%)	854 (91.2%)	67 (7.2%)
Unannotated	13 (9.4%)	120 (87.0%)	5 (3.6%)

Table S13. Changes across compartments | Hoel. Related to the Discussion and SuppData7.

**Compartment**	**Down**	**No change**	**Up**
Intracellular	910 (23.3%)	2669 (68.2%)	332 (8.5%)
Membrane	160 (9.3%)	1339 (78.0%)	217 (12.6%)
Membrane and secreted isoforms	13 (7.1%)	152 (82.6%)	19 (10.3%)
Secreted	104 (8.0%)	986 (76.3%)	202 (15.6%)
Unannotated	35 (15.7%)	165 (74.0%)	23 (10.3%)
